# Selectfluor and alcohol-mediated synthesis of bicyclic oxyfluorination compounds by Wagner–Meerwein rearrangement

**DOI:** 10.3762/bjoc.20.129

**Published:** 2024-07-01

**Authors:** Ziya Dağalan, Muhammed Hanifi Çelikoğlu, Saffet Çelik, Ramazan Koçak, Bilal Nişancı

**Affiliations:** 1 Department of Chemistry, Faculty of Science, Ataturk University, Erzurum, Turkey; 2 Technology Research and Development Application and Research Center, Trakya University, Edirne, Turkey; 3 Department of Chemistry, Faculty of Arts and Sciences, Amasya University, Amasya, Turkey

**Keywords:** alkoxyfluorine compounds, bicyclic alkene, oxyfluorination, selectfluor, Wagner–Meerwein rearrangement

## Abstract

Herein, we report the first environmentally friendly systematic fluoroalkoxylation reactions in bicyclic systems. New oxyfluorination products were obtained with excellent yields (up to 98%) via Wagner–Meerwein rearrangement using benzonorbornadiene and the chiral natural compound (+)-camphene as bicyclic alkenes, selectfluor as an electrophilic fluorine source, and water and various alcohols as nucleophile sources. The structure of bicyclic oxy- and alkoxyfluorine compounds was determined by NMR and QTOF-MS analyses.

## Introduction

**Figure 1:**
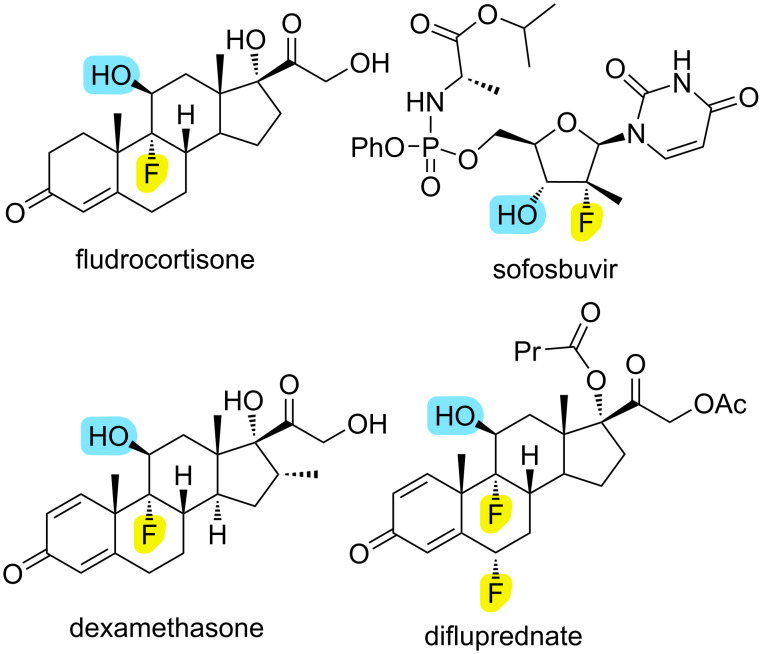
Organofluorine derived drugs.

Organofluorines are of great importance in the pharmaceutical and agrochemical industries, as the presence of fluorine has a serious effect on the biological activities of organic compounds by changing their metabolic stability, hydrogen bonding ability, lipophilicity, solubility, bioavailability, conformation and general structure [1–4]. About 20% of commercially available drugs contain fluorine, and this ratio is estimated to increase further [5–6]. Among organoﬂuorines, oxyﬂuorines are an important subclass used as an active ingredient in many different drugs such as fludrocortisone (the first fluorine-containing commercial drug) [7–8], sofosbuvir (antihepatitis C) [9], dexamethasone (to treat ashma, severe allergies) [10], difluprednate (ocular anti-inﬂammatory) [11–12] and many more (Figure 1). On the other hand, with unusual geometry and high reactivity norbornadiene and benzonorbornadiene derivative bicyclic compounds attract great attention by researchers with their use as building blocks in different application areas such as polymers, solar energy storage materials, supramolecular and bioactive compounds [13–17]. To the best of our knowledge, although the oxyfluorination of various olefins with water and alcohols is known in the literature [18–26], there is no systematic study on the oxyfluorination of bicyclic alkenes. We previously developed a dihomohalogenation method using selectfluor as an oxidant [27]. Herein, we synthesized bicyclic oxy- and alkoxyfluorine compounds using selectflour as an electrophilic fluorination reagent, water and various alcohols as an nucleophile.

## Results and Discussion

In this study, benzonorbornadiene (**1a**) and the chiral natural product (+)-camphene (**1b**) were used as bicyclic alkenes. Safe, easily soluble, easy to use, stable solid, reactive and commercial available selectfluor [18,27–28] was selected for electrophilic fluorination source. Water and various alcohols were used as nucleophiles.

First, optimization experiments were carried out for fluoroalkoxy reactions with benzonorbornadiene (**1a**, Table 1). As a result of experiments conducted in six different solvents at room temperature with 1.0 equivalent of selectflor and 1.0 equivalent of methanol, it was observed that there was a 12% conversion with CH_3_CN and a 10% conversion with nitromethane, while no conversion occurred with the other solvents including, CH_2_Cl_2_, EtOAc, 1,4-dioxane and DMF (Table 1, entries 1–6). To see the effect of reactant ratios on yields, when reactants were gradually increased at room temperature, the best result was obtained with 1.2 equivalents of selectfluor and 2.4 equivalents of methanol with 21% conversion (Table 1, entry 7). There was no significant change at higher equivalents. At 50 °C, 30% conversion was achieved with 1.2 equivalents of selectfluor and 2.4 equivalents of methanol for one hour, while at 90 °C, a 67% conversion was obtained (Table 1, entries 8 and 9). Finally, when the reaction time was increased to two hours at 90 °C, the product was obtained with a 98% conversion (Table 1, entry 10).

**Table 1:** Optimizing the conditions for the oxyfluorination of bicyclic alkenes^a^.

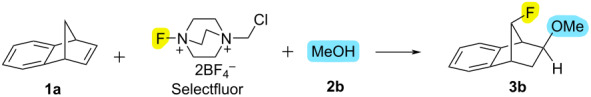						

Entry	Solvent	Selectfluor (equiv)	CH_3_OH (equiv)	Temperature (°C)	Time	Conversion (%)

1	CH_3_CN	1	1	rt	1 h	12

2	CH_2_Cl_2_	1	1	rt	1 h	–

3	EtOAc	1	1	rt	1 h	–

4	1,4-dioxane	1	1	rt	1 h	–

5	DMF	1	1	rt	1 h	–

6	CH_3_NO_2_	1	1	rt	1 h	10

7	CH_3_CN	1.2	2.4	rt	1 h	21

8	CH_3_CN	1.2	2.4	50 °C	1 h	30

9	CH_3_CN	1.2	2.4	90 °C	1 h	67

**10**	**CH** **_3_** **CN**	**1.2**	**2.4**	**90 °C**	**2 h**	**98**

^a^Reaction conditions: Benzonorbornadiene (**1a**, 0.5 mmol), selectfluor (215 mg, 0.61 mmol) and MeOH (1.2 mmol), 2 mL of CH_3_CN, 2 h and 90 °C. Conversions were calculated by ^1^H NMR with 1,3-dinitrobenzene as an internal standard.

**Scheme 1:**
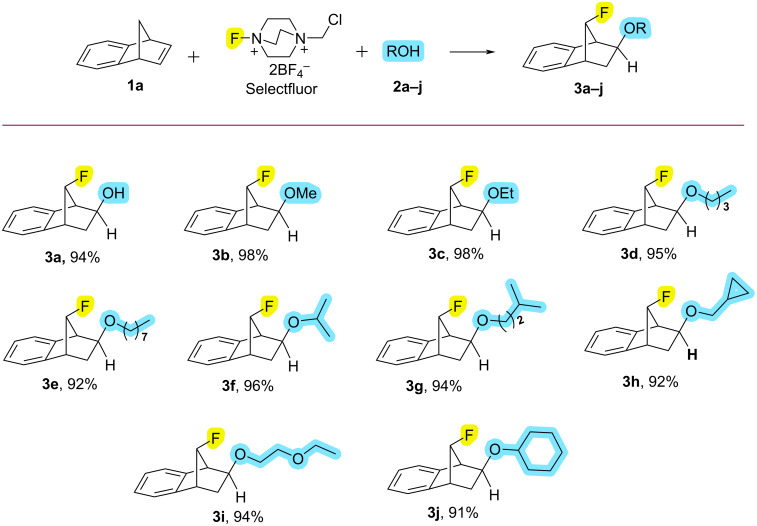
Oxyfluorination of benzonorbornadien (**1a**) with Selectfluor and alcohols. All reactions were carried out using 0.5 mmol of benzonorbornadiene (**1a**), 0.6 mmol of selectfluor, and 1.2 mmol alcohol in 2 mL of CH_3_CN at 90 °C for 2 h. Isolated yields.

**Scheme 2:**
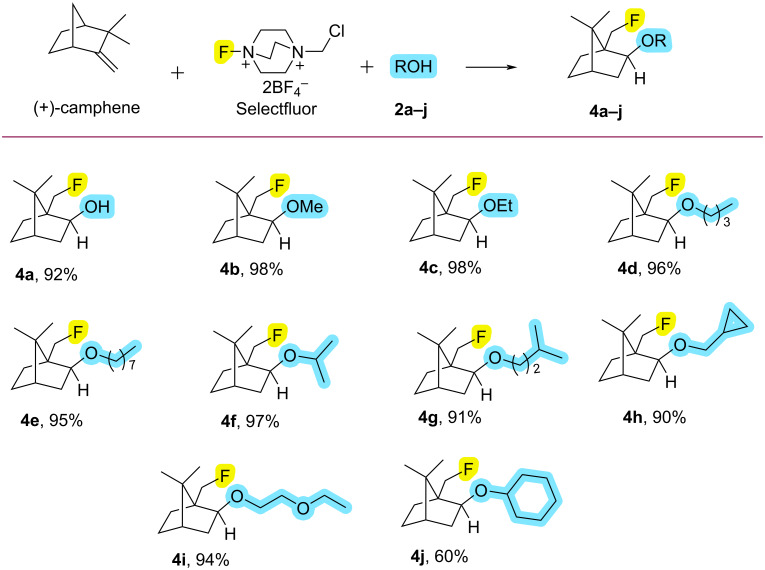
Oxyfluorination of (+)-camphene (**1b**) with selectfluor and alcohols. All reactions were carried out using 0.5 mmol of (+)-camphene, 0.6 mmol of selectfluor, and 1.2 mmol alcohol in 2 mL of CH_3_CN at 90 °C for 2 h. Isolated yields.

After obtaining the optimum fluoroalkoxylation conditions from benzonorbornadiene (**1a**), the reactions of benzonorbornadiene (**1a**) with selectfluor and 10 different alcohol derivatives were examined (Scheme 1). Under optimum conditions, fluoroalkoxy compounds **3a–j** were obtained in excellent yields (91–98%) by the reaction of benzonorbornadiene (**1a**) with selectfluor and alcohols (Scheme 1). The configurations of fluoroalkoxy compounds **3a–j** were confirmed by the COSY 2D-NMR spectrum of compound **3a** (Supporting Information File 1). Additionally, (+)-camphene (**1b**), a chiral natural product, was used as another alkene for fluoroalkoxy reactions. From (+)-camphene (**1b**), fluoroalkoxy compounds **4a–j** were also obtained in very good yields (60–98%, Scheme 2). Since the reaction mechanism proceeding with a Wagner–Meerwein rearrangement does not cause racemization or a diastereomeric mixture and preserves the initial enantiomeric excess in the camphene's fluoroalkoxy derivatives (Scheme 4, below), optical rotations of the fluoroalkoxy derivatives of camphene **4a–j** were also determined (Supporting Information File 1).

**Scheme 3:**
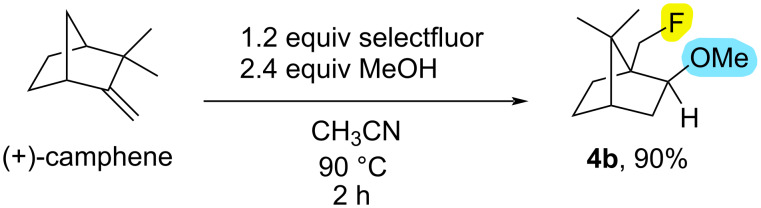
Scale-up experiments. Reaction conditions: (+)-Camphene (**1b**) (1.0 g, 7.34 mmol), selectfluor (3.12 g, 8.81 mmol), MeOH (17.62 mmol), CH_3_CN (20 mL), 90 °C, 2 h.

In order to demonstrate the gram-scale applicability of fluoroalkoxylation reactions in bicyclic systems using optimized reaction conditions with (+)-camphene (**1b**, 1.0 g, 7.34 mmol), scale-up experiments were conducted. The isolated yield of **4b** (1.26 g, 90% yield) is quite satisfactory, as can be seen from Scheme 3.

**Scheme 4:**
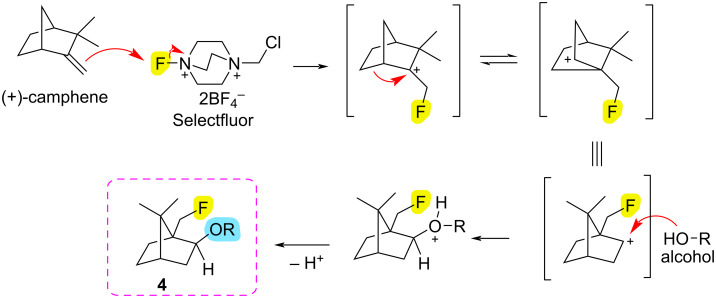
Proposed mechanism for fluoroalkoxylation of (+)-camphene by Wagner–Meerwein rearrangement.

For the fluoroalkoxylation, we propose the mechanism given in Scheme 4. In this mechanism, first the double bond in (+)-camphene attacks the fluorine in the selectfluor and a carbocation is formed by bonding with fluorine. Subsequently, fluoroalkoxy compound **4** is formed by Wagner–Meerwein rearrangement followed by alcohol addition and deprotonation.

## Conclusion

New bicyclic fluoroalkoxy compounds were synthesized by a molecular fluorine and metal-free methodology. An environmentally friendly approach was pursued by using safe, easily soluble, easy to use, stable, solid and reactive selectfluor as an electrophilic fluorination reagent, and water and various alcohols as a nucleophile source. Besides being novel, the presented oxyfluorination protocol provides distinct advantageous such as (i) the methodology does not require the presence of any metal moities, (ii) enables the synthesis of corresponding oxyfluorinated analogues with high yields and selectivity, (iii) allows derivatization of natural chiral molecules, (iv) uses a safe solvent in mild reaction parameters. We hope that these potentially biologically active bicyclic fluoroalkoxy compounds will find a place in various application areas in biological systems.

## Supporting Information

File 1 Experimental procedures, copies of ^1^H NMR, ^13^C NMR, and HRMS(Q-TOF) spectra.

## Data Availability

All data that supports the findings of this study is available in the published article and/or the supporting information to this article.
