# Supplementary material for: Selectfluor and alcohol-mediated synthesis of bicyclic oxyfluorination compounds by Wagner–Meerwein rearrangement
Source: Beilstein J Org Chem. 2024 Jul 1;20:1462–7. doi: 10.3762/bjoc.20.129 (PMC11228819; doi:10.3762/bjoc.20.129)
Supplement: File 1 — Experimental procedures, copies of 1H NMR, 13C NMR, and HRMS(Q-TOF) spectra. [file Beilstein_J_Org_Chem-20-1462-s001.pdf]

BEILSTEIN JOURNAL OF ORGANIC CHEMISTRY

Supporting Information

for

Selectfluor and alcohol-mediated synthesis of bicyclic oxyfluorination compounds by Wagner–Meerwein rearrangement

Ziya Dağalan, Muhammed Hanifi Çelikoğlu, Saffet Çelik, Ramazan Koçak and Bilal Nişancı

Beilstein J. Org. Chem. **2024,** *20,* 1462–1467. doi:10.3762/bjoc.20.129

Experimental procedures, copies of ^{1}H NMR, ^{13}C NMR, and HRMS(Q-TOF) spectra

License and Terms: This is a supporting information file under the terms of the Creative Commons Attribution License (https://creativecommons.org/licenses/by/4.0). Please note that the reuse, redistribution and reproduction in particular requires that the author(s) and source are credited and that individual graphics may be subject to special legal provisions.
The license is subject to the *Beilstein Journal of Organic Chemistry* terms and conditions: (https://www.beilstein-journals.org/bjoc/terms)

Table of contents

Experimental S2

Genral procedure...S2

References..S11

^{1}H NMR, ^{13}C NMR, and HRMS spectra...S12

Experimental

General

All chemicals and solvents, purchased from Sigma-Aldrich, were used without further purification. Reactions that require heating were carried out under oil bath conditions. Reactions were monitored by thin-layer chromatography using Merck TLC Silica gel 60 F254 and the plates were inspected by 254 nm or 365 nm UV-ligth and/or by acquiring ^{1}H-NMR spectra. Column chromatography was performed over Merck Silica gel 60F (70–230 mesh ASTM). The ^{1}H and ^{13}C NMR spectra were recorded on a Varian-400 or a Bruker-400 spectrometer in $CDCl_3$ using tetramethylsilane as the interrnal reference. All spectra were recorded at 25 ^{o}C and coupling constants (*J* values) are given in Hz. Chemical shifts are given in parts per million (ppm). Abbreviations used to define the multiplicities are as follows: s = singlet; d = doublet; dd = doublet of doublets; m = multiplet. Optical rotations were determined with a 589 nm spectropolarimeter at 25 ^{o}C. IR Spectra spectra were recorded with VERTEX 70v FT-IR spectrometer. Mass spectra of unknown compounds were recorded on an AB-Sciex 4600 QTOF-MS.

General procedure

Benzonorbornadiene (**1a**) or (+)-camphene (**1b**) (0.5 mmol), selectfluor (215 mg, 0.61 mmol) and alcohol derivatives (2.4 mmol) were dissolved in 2 mL CH_3CN in an ACE pressure tube. The reaction mixture was stirred at 90 ^{o}C for 2 hours. The mixture was cooled to room temperature and the solvent was evaporated under reduced pressure. The crude reaction mixture was purified by column chromatography on silica gel (*n*-hexane/EtOAc (9:1)).

***(1R(S),4R(S),9R(S))-9-Fluoro-1,2,3,4-tetrahydro-1,4-methanonaphthalen-2-ol* (3a):**

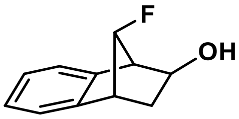

Colorless oil (97 mg, 94%). ^{1}H NMR (400 MHz, $CDCl_3$) δ 7.23 – 7.19 (m, 1H), 7.18 – 7.14 (m, 3H), 4.81 (d, *J* = 57.2 Hz, 1H), 4.00-3.88 (m, 1H), 3.54-3.47 (m, 2H), 2.18 (s, 1H), 2.10 – 2.01 (m, 2H). ^{13}C NMR (101 MHz, $CDCl_3$) δ 142.8 (d, *J* = 8.1 Hz), 138.8 (d, *J* = 10.6 Hz), 127.7, 127.2, 123.1, 122.2, 100.6 (d, *J* = 206.4 Hz), 74.1, 54.0 (d, *J* = 14.9 Hz), 46.4 (d, *J* = 18.4 Hz), 36.7. ^{19}F NMR (376 MHz, $CDCl_3$) δ -177.17 (d, *J* = 58.3 Hz). IR (KBr cm^{-1}): 2932, 2887, 2857, 1732,1452, 1391, 1184, 1082, 991, 980. (TOF MS) m/z (%): $[(M\text{-}H_2O)+H]^+$ calcd for $C_{11}H_9F$ 161.0761; found: 161,0773.

Structure:

The rearrangement mechanisms of addition reactions in norbornene-derived bicyclic systems and the products formed as a result of this mechanism are well known. In these systems, *syn-anti* and *endo-exo* configurations can be easily determined using

^{1}H NMR and 2D NMR (NEO, NEOSY, COSY, etc.) techniques (Daştan et al., 1996; Gültekin et al., 2023; Wakchaure et al., 2024; Zky et al. 1998; Moreno-Dorado et al., 2003).

Although the structures of the products formed as a result of alkene additions in norbornene-derived systems are well known, COSY 2D-NMR experiments of compound **3a** were performed to confirm the configurations of the products. When the COSY spectrum is examined, it is seen that Ha interacts with Hb and Hc, Hb interacts with Ha and Hc, and Hc interacts with Ha and Hc.

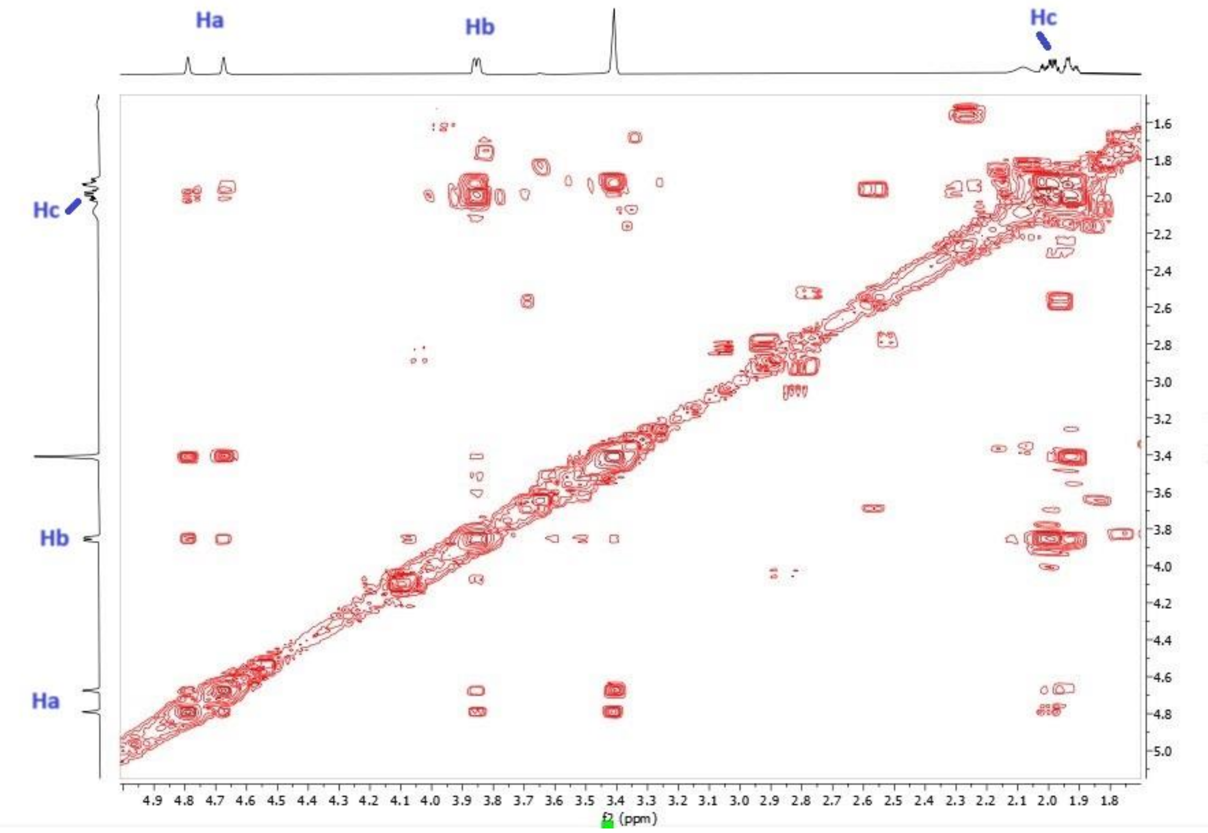

In norbornene-derived bicyclic systems, long-distance interactions of protons through four bonds are observed when they are zigzag (M or W) oriented. Otherwise, no interaction is observed (Balci, 2005). In the light of this information, when the possible structures fo**r 3a** are evaluated together with the COSY spectrum; the only structure in which all Ha, Hb and Hc protons can interact with each other is the **3a** structure. This proves the correctness of the configuration given for structure **3** in the manuscript, with fluorine as anti and OH as exo.

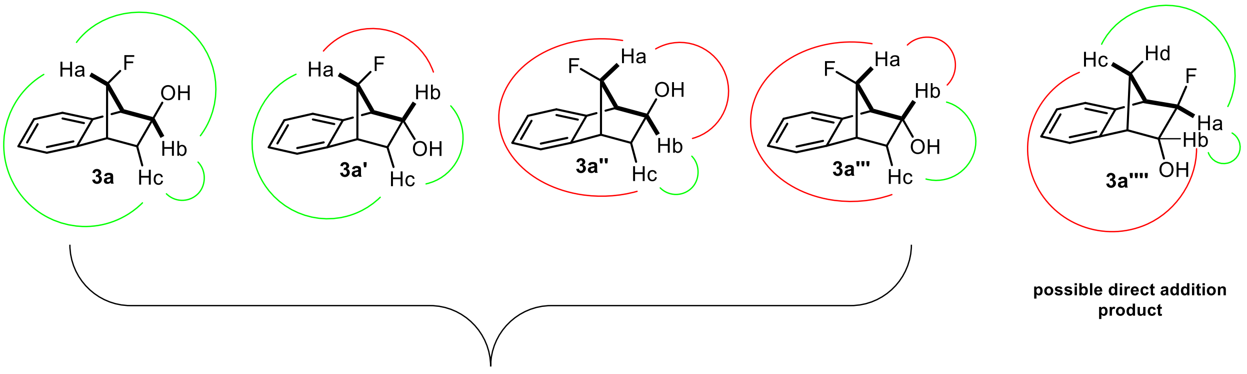

possible direct addition product

possible Wagner-Meerwein rearrangement products

(1R(S),4R(S),9R(S))-9-Fluoro-2-methoxy-1,2,3,4-tetrahydro-1,4-methanonaphthalene **(3b):**

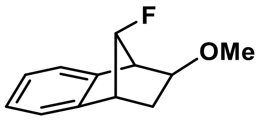

Colorless oil (101 mg, 98%). ^{1}H NMR (400 MHz, $CDCl_3$) δ 7.24-7.20 (m, 1H), 7.19-7.11 (m, 3H), 4.68 (d, *J* = 57.3 Hz, 1H), 3.62-3.55 (m, 1H), 3.53-3.47 (m, 1H), 3.47-3.43 (m, 1H), 3.41 (s, 3H), 2.15-2.06 (m, 1H), 1.98-1.89 (m, 1H). ^{13}C NMR (101 MHz, $CDCl_3$) δ 143.3 (d, *J* = 7.6 Hz), 139.4 (d, *J* = 10.4 Hz), 127.5, 127.1, 122.9, 122.3, 98.7 (d, *J* = 213.4 Hz), 83.0 (d, *J* = 2.3 Hz), 57.7, 50.6 (d, *J* = 16.3 Hz), 46.3 (d, *J* = 18.6 Hz), 32.9. ^{19}F NMR (376 MHz, $CDCl_3$) δ -178.50 (d, *J* = 57.9 Hz). IR (KBr cm^{-1}): 2980, 2943, 2886, 2820, 1734, 1464, 1350, 1242, 1209, 1099, 1047, 1003, 937, 750, 635,505, 422. (TOF MS) m/z (%): $(M+H)^+$ calcd for $C_{12}H_{13}FO$ 193.1023; found: 193.1022

(1R(S),4R(S),9R(S))-2-Ethoxy-9-fluoro-1,2,3,4-tetrahydro-1,4-methanonaphthalene **(3c):**

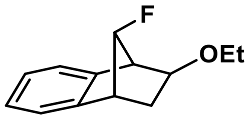

Colorless oil (101 mg, 98%).^{1}H NMR (400 MHz, $CDCl_3$) δ 7.23 – 7.19 (m, 1H), 7.19 – 7.14 (m, 3H), 4.67 (d, *J* = 57.1 Hz, 1H), 3.63 – 3.51 (m, 4H), 3.47 – 3.40 (m, 1H), 2.19-2.10 (m, 1H), 1.96-1.86 (m, 1H), 1.26 (t, *J* = 7.0 Hz, 3H). ^{13}C NMR (101 MHz, $CDCl_3$) δ 143.2 (d, *J* = 7.6 Hz), 139.6 (d, *J* = 10.4 Hz), 127.4, 127.0, 122.7, 122.2, 98.6 (d, *J* = 213.3 Hz), 81.0, 65.3, 51.0 (d, *J* = 16.0 Hz), 46.3 (d, *J* = 18.4 Hz), 33.1, 15.5. ^{19}F NMR (376 MHz, $CDCl_3$) δ -178.31 (d, *J* = 57.7 Hz). IR (KBr cm^{-1}): 2974, 2945, 1734, 1466, 1348, 1240, 1194, 1103, 1051, 1011, 750, 634. (TOF MS) m/z (%): $(M+H)^+$ calcd for $C_{15}H_{19}FO$ 207,1180; found: 207,1180.

***(1R(S),4R(S),9R(S))-2-Butoxy-9-fluoro-1,2,3,4-tetrahydro-1,4-methanonaphthalene* (3d):**

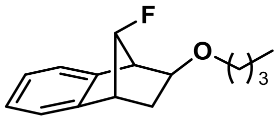

Colorless oil (98 mg, 95%). ^{1}H NMR (400 MHz, $CDCl_3$) δ 7.24 – 7.21 (m, 1H), 7.20 – 7.14 (m, 3H), 4.68 (d, *J* = 57.1 Hz, 1H), 3.65 – 3.42 (m, 5H), 2.21 – 2.09 (m, 1H), 2.00-1.86 (m, 1H), 1.68 – 1.56 (m, 2H), 1.50-1.36 (m, 2H), 0.96 (t, *J* = 7.4 Hz, 3H). ^{13}C NMR (101 MHz, $CDCl_3$) δ 143.2 (d, *J* = 7.7 Hz), 139.6 (d, *J* = 10.3 Hz), 127.3, 127.0, 122.7, 122.1, 98.6 (d, *J* = 213.9 Hz), 81.0, 69.8, 50.9 (d, *J* = 19.2 Hz), 46.2 (d, *J* = 18.3 Hz). 33.0, 32.0, 19.5, 14.0. ^{19}F NMR (376 MHz, $CDCl_3$) δ -178.34 (d, *J* = 57.6 Hz). IR (KBr cm^{-1}): 2955, 2926, 2866, 1724, 1464, 1348, 1194, 1092, 1051, 939, 748, 634, 494, 422. (TOF MS) m/z (%): $(M+H)^+$ calcd for $C_{15}H_{19}FO$ 235,1493; found: 235,1495.

***(1R(S),4R(S),9R(S))-9-Fluoro-2-(octyloxy)-1,2,3,4-tetrahydro-1,4-methanonaphthalene* (3e):**

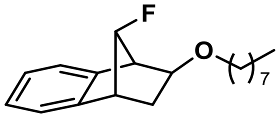

Colorless oil (133 mg, 92%). ^{1}H NMR (400 MHz, $CDCl_3$) δ 7.24 – 7.20 (m, 1H), 7.20 – 7.12 (m, 3H), 4.68 (d, *J* = 57.1 Hz, 1H), 3.61 – 3.43 (m, 5H), 2.18-2.08 (m, 1H), 1.96-1.86 (m, 1H), 1.65 – 1.59 (m, 2H), 1.36 – 1.27 (m, 10H), 0.90 (t, *J* = 6.8 Hz, 3H). ^{13}C NMR (101 MHz, $CDCl_3$) δ 143.3 (d, *J* = 7.7 Hz), 139.6 (d, *J* = 10.5 Hz), 127.4, 127.0, 122.8, 122.2, 98.6 (d, *J* = 214.2 Hz), 81.1, 70.2, 50.9 (d, *J* = 17.2 Hz), 46.3 (d, *J* = 18.1 Hz), 33.0, 31.9, 30.0, 29.6, 29.4, 26.3, 22.8, 14.2. ^{19}F NMR (376 MHz, $CDCl_3$) δ -178.26 (d, *J* = 57.3 Hz). IR (KBr cm^{-1}): 2926, 2855, 1464, 1348, 1105, 1053, 1009, 748, 494, 422**.** (TOF MS) m/z (%): $(M+H)^+$ calcd for $C_{19}H_{27}FO$ 291.2119; found: 291.2116.

***(1R(S),4R(S),9R(S))-9-Fluoro-2-isopropoxy-1,2,3,4-tetrahydro-1,4-methanonaphthalene* (3f):**

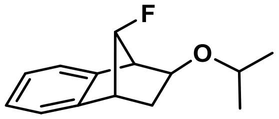

Colorless oil (105 mg, 96%). ^{1}H NMR (400 MHz, $CDCl_3$) δ 7.25 – 7.21 (m, 1H), 7.20 – 7.15 (m, 3H), 4.68 (d, *J* = 57.2 Hz, 1H), 3.77 – 3.66 (m, 2H), 3.54-3.48 (m, 1H),3.47-3.40(m, 1H),2.19-2.08 (m, 1H), 1.97-1.87 (m, 1H), 1.24 (d, *J* = 6.1 Hz, 3H), 1.20 (d, *J* = 6.1 Hz, 3H). ^{13}C NMR (101 MHz, $CDCl_3$) δ 143.2 (d, *J* = 7.6 Hz), 139.8 (d, *J* = 10.4 Hz), 127.3, 126.9, 122.7, 122.2, 98.6 (d, *J* = 213.6 Hz), 78.5, 70.7 (d, *J* = 2.0 Hz). 51.8 (d, *J* = 16.5 Hz), 46.4 (d, *J* = 18.4 Hz), 33.6, 22.6, 22.5. ^{19}F NMR (376 MHz, $CDCl_3$) δ -178.04 (d, *J* = 57.6 Hz). IR (KBr cm^{-1}): 2973, 2947, 2879, 1468, 1370, 1330, 1241, 1151, 1134, 1085, 1053, 1013, 986, 749, 638, 499. (TOF MS) m/z (%): $(M+H)^+$ calcd for $C_{14}H_{17}FO$ 221,1336; found: 221,1338.

***(1R(S),4R(S),9R(S))-9-Fluoro-2-(isopentyloxy)-1,2,3,4-tetrahydro-1,4-methanonaphthalene* (3g):**

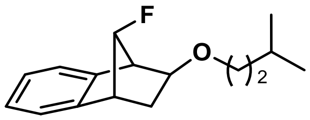

Colorless oil (116 mg, 94%). ^{1}H NMR (400 MHz, $CDCl_3$) δ 7.24 – 7.20 (m, 1H), 7.19 – 7.14 (m, 3H), 4.68 (d, *J* = 57.1 Hz, 1H), 3.64 – 3.40 (m, 5H), 2.20-2.05 (m, 1H), 2.00-1.83 (m, 1H), 1.77 – 1.68 (m, 1H), 1.57 – 1.47 (m, 2H), 0.94 (d, *J* = 2.5 Hz, 3H), 0.92 (d, *J* = 2.5 Hz, 3H). ^{13}C NMR (101 MHz, $CDCl_3$) δ 143.3 (d, *J* = 7.7 Hz), 139.6 (d, *J* = 10.0 Hz), 127.4, 127.0, 122.8, 122.2, 98.6 (d, *J* = 213.7 Hz), 81.1 (d, *J* = 2.1 Hz), 68.5, 50.9 (d, *J* = 16.2 Hz), 46.3 (d, *J* = 18.7 Hz), 38.8, 33.1, 25.2, 22.8. ^{19}F NMR (376 MHz, $CDCl_3$) δ -178.30 (d, *J* = 57.1 Hz). IR (KBr cm^{-1}): 2961, 2933, 2866, 1473, 1354, 1197, 1102, 1055, 1022, 936, 751, 641, 494. (TOF MS) m/z (%): $(M+H)^+$ calcd for $C_{16}H_{21}FO$ 249.1649; found: 249.1647.

***(1R(S),4R(S),9R(S))-2-(Cyclopropylmethoxy)-9-fluoro-1,2,3,4-tetrahydro-1,4-methanonaphthalene* (3h):**

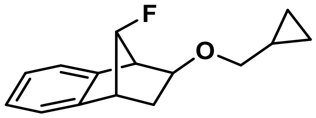

Colorless oil (106 mg, 92%). ^{1}H NMR (400 MHz, $CDCl_3$) δ 7.22-7.19 (m, 1H), 7.18-7.14 (m, 3H), 4.67 (d, *J* = 57.2 Hz, 1H), 3.66-3.60 (m, 1H), 3.60- 3.56 (m, 1H), 3.46-3.42 (m, 1H), 3.35 (d, *J* = 6.8 Hz, 2H), 2.18 – 2.11 (m, 1H), 1.95-1.86 (m, 1H), 1.14-1.06(m, 1H), 0.58-0.54 (m, 2H), 0.25 – 0.21 (m, 2H). ^{13}C NMR (101 MHz, $CDCl_3$) δ 143.3 (d, *J* = 7.7 Hz), 139.6 (d, *J* = 10.0 Hz), 127.4, 127.0, 122.8, 122.2, 98.6 (d, *J* = 213.3 Hz), 80.8 (d, *J* = 2.2 Hz), 74.8, 51.0 (d, *J* = 16.3 Hz), 46.3 (d, *J* = 18.6 Hz), 33.1, 10.9, 3.4. ^{19}F NMR (376 MHz, $CDCl_3$) δ -178.24 (d, *J* = 57.7 Hz). IR (KBr cm^{-1}): 3071, 2984, 2948, 2870, 1726, 1471, 1352, 1193, 1084, 1052, 1016, 943, 751, 642, 500. (TOF MS) m/z (%): $(M+H)^+$ calcd for $C_{15}H_{17}FO$ 233.1336; found: 233.1337.

***(1R(S),4R(S),9R(S))-2-(2-Ethoxyethoxy)-9-fluoro-1,2,3,4-tetrahydro-1,4-methanonaphthalene* (3i):**

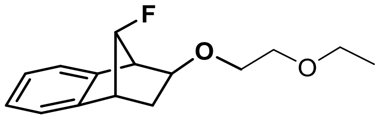

Colorless oil (117 mg, 94%). ^{1}H NMR (400 MHz, $CDCl_3$) δ 7.21 – 7.18 (m, 1H), 7.17 – 7.13 (m, 3H), 4.66 (dd, *J* = 57.0, 1.9 Hz, 1H), 3.69 – 3.59 (m, 6H), 3.55 (q, *J* = 6.8 Hz, 2H), 3.46-3.41 (m, 1H), 2.18-2.11 (m, 1H), 1.95-1.88 (m, 1H), 1.21 (t, *J* = 7.0 Hz, 3H).^{13}C NMR (101 MHz, $CDCl_3$) δ 143.2 (d, *J* = 7.7 Hz), 139.5 (d, *J* = 10.7 Hz), 127.4, 127.1, 122.9, 122.2, 98.6 (d, *J* = 213.4 Hz), 81.7 (d, *J* = 2.2 Hz), 70.0, 69.4, 66.8, 50.9 (d, *J* = 16.6 Hz), 46.3 (d, *J* = 18.4 Hz), 33.1, 15.3. ^{19}F NMR (376 MHz, $CDCl_3$) δ -178.23 (d, *J* = 57.1 Hz). IR (KBr cm^{-1}): 2986, 2944, 2868, 1734, 1469, 1357, 1239, 1173, 1102,

1050, 755, 643, 495. (TOF MS) m/z (%): [(M-$C_4H_9O_2$)+H]$^+$ calcd for $C_{11}H_{10}F$ 161.0767; found: 161.0761

***(1R(S),4R(S),9R(S))-2-(Cyclohexyloxy)-9-fluoro-1,2,3,4-tetrahydro-1,4-methanonaphthalene* (3j):**

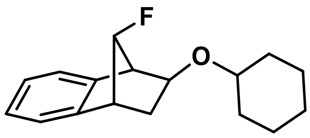

Colorless oil (118 mg, 91%). ^{1}H NMR (400 MHz, $CDCl_3$) δ 7.23-7.18 (m, 1H), 7.18 – 7.09 (m, 3H), 4.65 (d, J = 57.2 Hz, 1H), 3.78-3.66 (m, 1H), 3.52 – 3.39 (m, 2H), 3.37-3.24 (m, 1H), 2.18-2.06 (m, 1H), 2.02 – 1.83 (m, 3H), 1.81 – 1.71 (m, 2H), 1.58 – 1.51 (m, 1H), 1.39 – 1.16 (m, 5H). ^{13}C NMR (101 MHz, $CDCl_3$) δ 143.1 (d, J = 7.6 Hz), 139.7 (d, J = 10.6 Hz), 127.2, 126.8, 122.6, 122.1, 98.6 (d, J = 213.8 Hz), 78.3, 77.2, 51.8 (d, J = 16.8 Hz), 46.3 (d, J = 18.3 Hz), 33.6, 32.9, 32.8, 25.7, 24.5, 24.5. ^{19}F NMR (376 MHz, $CDCl_3$) δ -178.00 (d, J = 57.2 Hz). IR (KBr cm^{-1}): 2983, 2935, 2858, 1470, 1358, 1198, 1087, 1059, 903, 729, 652, 522. (TOF MS) m/z (%): (M+H)$^+$ calcd for $C_{17}H_{21}FO$ 261.1649; found: 261.1648.

***(1R,4S)-1-(Fluoromethyl)-7,7-dimethylbicyclo[2.2.1]heptan-2-ol* (4a):**

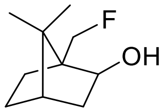

Colorless oil (79 mg, 92%). $[\alpha]_D$ = -5.0 (c = 1.0, EtOH). ^{1}H NMR (400 MHz, $CDCl_3$) δ 4.79 (dd, J = 47.7, 9.3 Hz, 1H), 4.48 (dd, J = 47.7, 9.3 Hz, 1H), 3.95 (dd, J = 7.9, 3.9 Hz, 1H), 1.80 – 1.70 (m, 4H), 1.27 – 1.20 (m, 2H), 1.12 (s, 3H), 1.08 – 1.04 (m, 1H), 0.97-0.93 (m, 1H), 0.89 (s, 3H). ^{13}C NMR (101 MHz, $CDCl_3$) δ 84.8 (d, J = 161.9 Hz), 52.9(d, J = 15.9 Hz), 46.9 (d, J = 7.0 Hz), 46.2, 40.3, 29.4 (d, J = 2.5 Hz), 27.1, 21.1, 20.6 (2C signal overlaps).^{19}F NMR (376 MHz, $CDCl_3$) δ -125.6 (ddd, J = 293.1, $J_{H\text{-}F}$ = 57.3, 55.5 Hz). IR (KBr cm^{-1}): 2966, 2890, 1729, 1453, 1376, 1259, 1083, 1061, 1010, 916, 807, 753, 631, 518. (TOF MS) m/z (%): (M+Na)$^+$ calcd for $C_{10}H_{17}FO$ 195.1156; found: 195.1154.

***(1R,4S)-1-(Fluoromethyl)-2-methoxy-7,7-dimethylbicyclo[2.2.1]heptane* (4b):**

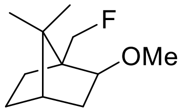

Colorless oil (91mg, 98%). $[\alpha]_D$ = -4.92 (c = 1.42, EtOH). ^{1}H NMR (400 MHz, $CDCl_3$) δ 4.71 (dd, J = 47.3, 9.3 Hz, 1H), 4.42 (dd, J = 48.1, 9.3 Hz, 1H), 3.36 (dd, J = 7.6, 3.5 Hz, 1H), 3.24 (s, 3H), 1.82 – 1.62 (m, 5H), 1.26 – 1.15 (m, 2H), 1.04 (s, 3H), 0.90 (s, 3H). ^{13}C NMR (101 MHz, $CDCl_3$) δ 86.2, 83.9 (d, J = 163.0 Hz), 56.9, 53.0 (d, J = 16.6 Hz), 46.9 (d, J = 6.3 Hz), 46.1, 38.0, 29.4 (d, J = 2.8 Hz), 27.1, 21.2, 20.6. ^{19}F NMR (376 MHz, $CDCl_3$) δ -120.34 (dd, J = 292.6, $J_{H\text{-}F}$ = 55.6 Hz). IR (KBr cm^{-1}): 3312, 2961,

2886, 1653, 1539, 1459, 1369, 1298, 1203, 1099, 981, 914, 729, 649, 592. (TOF MS) m/z (%): [(M-MeOH)+H]$^+$ calcd for $C_{10}H_{15}F$ 155.1231; found: 155.1230.

***(1R,4S)-2-Ethoxy-1-(fluoromethyl)-7,7-dimethylbicyclo[2.2.1]heptane* (4c):**

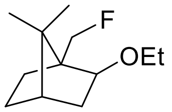

Colorless oil (98 mg, 98%). $[\alpha]_D$ = -10.34 (c = 1.45, EtOH). ^{1}H NMR (400 MHz, $CDCl_3$) δ 4.73 (dd, *J* = 47.3, 9.2 Hz, 1H), 4.42 (dd, *J* = 48.2, 9.2 Hz, 1H), 3.52 – 3.42 (m, 2H), 3.39 – 3.29 (m, 1H), 1.83 – 1.62 (m, 6H), 1.27 – 1.25 (m, 1H), 1.13 (s, 3H), 1.07 (s, 3H), 0.90 (s, 3H). ^{13}C NMR (101 MHz, $CDCl_3$) δ 84.1 (d, *J* = 2.6 Hz), 84.1 (d, *J* = 162.7 Hz), 64.8, 52.9 (d, *J* = 16.4 Hz), 46.9 (d, *J* = 6.2 Hz), 46.2, 38.9, 29.3 (d, *J* = 2.8 Hz), 27.1, 21.3, 20.6, 15.7. ^{19}F NMR (376 MHz, $CDCl_3$) δ -126.99 (ddd, *J* = 290.8, J_{H-F} = 57.8, 54.5 Hz). IR (KBr cm^{-1}): 2962, 2885, 1724, 1684, 1643, 1463, 1377, 1268, 1178, 1110, 1092, 1007, 989, 803. (TOF MS) m/z (%): [(M-EtOH)+H]$^+$ calcd for $C_{10}H_{15}F$ 155.1231; found: 155.1229.

***(1R,4S)-2-Butoxy-1-(fluoromethyl)-7,7-dimethylbicyclo[2.2.1]heptane* (4d):**

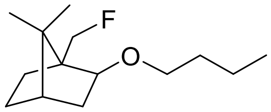

Colorless oil (109 mg, 96%). $[\alpha]_D$ = -7.07 (c = 0.99, EtOH). ^{1}H NMR (400 MHz, Chloroform-*d*) δ 4.73 (dd, *J* = 47.3, 9.2 Hz, 1H), 4.42 (dd, *J* = 48.2, 9.2 Hz, 1H), 3.44 – 3.37 (m, 2H), 3.28-3.23(m, 1H), 1.82 – 1.68 (m, 5H), 1.64 – 1.58 (m, 2H), 1.51 – 1.43 (m, 2H), 1.37 – 1.33 (m, 2H), 1.06 (s, 3H), 0.92 – 0.87 (m, 6H). ^{13}C NMR (101 MHz, $CDCl_3$) δ 84.2, 84.1 (d, *J* = 162.3 Hz), 69.0, 52.9 (d, *J* = 16.4 Hz), 46.9 (d, *J* = 6.1 Hz), 46.1, 38.7, 32.3, 29.2 (d, *J* = 2.8 Hz), 27.1, 21.3, 20.6, 19.7, 14.1. ^{19}F NMR (376 MHz, $CDCl_3$) δ -123.43 (dd, *J* = 290.3, 55.0 Hz), -127.01 (ddd, *J* = 289.8, J_{H-F} = 60.3, 55.0 Hz), -130.59 (dd, *J* = 289.9, 60.3 Hz). IR (KBr cm^{-1}): 2958, 2939, 2874, 1728, 1464, 1377, 1187, 1099, 984, 804. (TOF MS) m/z (%): (M+Na)$^+$ calcd for $C_{14}H_{25}FO$ 251.1782; found: 251.1832.

***(1R,4S)-1-(Fluoromethyl)-7,7-dimethyl-2-(octyloxy)bicyclo[2.2.1]heptane* (4e):**

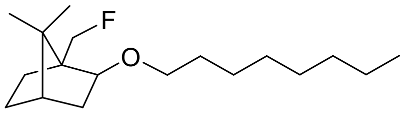

Colorless oil (134 mg, 95%). $[\alpha]_D$ = -6.97 (c = 1.29, EtOH). ^{1}H NMR (400 MHz, Chloroform-*d*) δ 4.73 (dd, *J* = 47.3, 9.2 Hz, 1H), 4.43 (dd, *J* = 48.2, 9.2 Hz, 1H), 3.48 – 3.35 (m, 2H), 3.32 – 3.18 (m, 1H), 1.84 – 1.46 (m, 9H), 1.31 – 1.25 (m, 10H), 1.06 (s, 3H), 0.92 – 0.84 (m, 6H).^{13}C NMR (101 MHz, $CDCl_3$) δ 84.3 (d, *J* = 2.9 Hz), 84.0, (d, *J* = 163.0 Hz), 69.4, 52.9 (d, *J* = 16.5 Hz), 46.9 (d, *J* = 5.9 Hz), 46.2, 38.8, 32.0, 30.2, 29.6, 29.5, 29.3 (d, *J* = 2.9 Hz), 27.1, 26.5, 22.8, 21.3, 20.6, 14.2. ^{19}F NMR (376 MHz, $CDCl_3$) δ -127.00 (ddd, *J* = 291.2, J_{H-F} = 57.6, 54.7 Hz). IR (KBr cm^{-1}): 2925, 2846,

1455, 1371, 1185, 1096, 988. (TOF MS) m/z (%): $(M+Na)^+$ calcd for $C_{18}H_{35}FO$ 309.2564; found: 309.2573.

***(1R,4S)-1-(Fluoromethyl)-2-isopropoxy-7,7-dimethylbicyclo[2.2.1]heptane* (4f):**

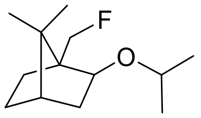

Colorless oil (103 mg, 97%). $[\alpha]_D$ = -10.32 (c = 1.55, EtOH). ^1H NMR (101 MHz, $CDCl_3$) δ 4.72 (dd, *J* = 47.3, 9.1 Hz, 1H), 4.40 (dd, *J* = 48.2, 9.1 Hz, 1H), 3.55 – 3.47 (m, 2H), 1.87 – 1.60 (m, 6H), 1.10 – 1.05 (m, 9H), 0.89 (s, 3H), 0.88 – 0.86 (m, 1H). ^{13}C NMR (101 MHz, $CDCl_3$) δ 84.3 (d, *J* = 162.4 Hz), 81.9 (d, *J* = 2.5 Hz), 70.9, 52.7 (d, *J* = 16.3 Hz), 47.0 (d, *J* = 6.8 Hz), 46.2, 40.2, 29.3 (d, *J* = 2.7 Hz), 27.1, 23.4, 22.3, 21.3, 20.7. ^{19}F NMR (376 MHz, $CDCl_3$) δ -127.31 (ddd, *J* = 288.7, $J_{H\text{-}F}$ = 58.1, 53.7 Hz). IR (KBr cm^{-1}): 3418, 2958, 2932, 2871, 1721, 1454, 1381, 1122, 1075, 978. (TOF MS) m/z (%): $[(M-C_3H_7OH)+H]^+$ calcd for $C_{10}H_{15}F$ 155.1231; found: 155.1229.

***(1R,4S)-1-(Fluoromethyl)-2-(isopentyloxy)-7,7-dimethylbicyclo[2.2.1]heptane* (4g):**

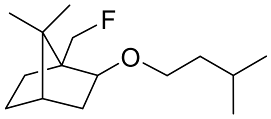

Colorless oil (110 mg, 91%). $[\alpha]_D$ = -7.25 (c = 1.24, EtOH). ^1H NMR (101 MHz, $CDCl_3$) δ 4.72 (dd, *J* = 47.3, 9.1 Hz, 1H), 4.42 (dd, *J* = 48.2, 9.2 Hz, 1H), 3.45 – 3.40 (m, 2H), 3.31 – 3.23 (m, 1H), 1.81 – 1.63 (m, 7H), 1.43 – 1.35 (m, 3H), 1.06 (s, 3H), 0.90 – 0.87 (m, 9H). ^{13}C NMR (101 MHz, $CDCl_3$) δ 84.3 (d, *J* = 3.0 Hz), 84.1 (d, *J* = 162.6 Hz), 67.6, 52.9 (d, *J* = 16.7 Hz), 46.2, 39.1, 38.7, 29.3, 29.2, 27.1, 25.2, 22.8, 22.7, 21.3, 20.6. ^{19}F NMR (376 MHz, $CDCl_3$) δ -127.01 (ddd, *J* = 291.5, $J_{H\text{-}F}$ = 60.7, 57.6 Hz). IR (KBr cm^{-1}): 2960, 2937, 2872, 1731, 1462, 1365, 1259, 1189, 1088, 977, 805. (TOF MS) m/z (%): $[(M-C_5H_{10}OH)+H]^+$ calcd for $C_{10}H_{15}F$ 155.1231; found: 155.1230.

***(1R,4S)-2-(Cyclopropylmethoxy)-1-(fluoromethyl)-7,7-dimethylbicyclo [2.2.1]heptane* (4h):**

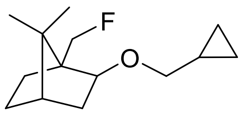

Colorless oil (100 mg, 90%). $[\alpha]_D$ = -5.71 (c = 0.7, EtOH). ^1H NMR (101 MHz, $CDCl_3$) δ 4.73 (dd, *J* = 47.3, 9.2 Hz, 1H), 4.42 (dd, *J* = 48.2, 9.2 Hz, 1H), 3.45 (dd, *J* = 7.7, 3.5 Hz, 1H), 3.21 (dd, *J* = 6.4, 1.5 Hz, 2H), 1.81 – 1.67 (m, 4H), 1.63 – 1.58 (m, 1H), 1.27-1.22 (m, 1H), 1.07 (s, 3H), 1.04 (s, 1H), 0.93 (s, 1H), 0.89 (s, 3H), 0.46 – 0.42 (m, 2H), 0.19 – 0.13 (m, 2H). ^{13}C NMR (101 MHz, $CDCl_3$) δ 84.0 (d, *J* = 162.9 Hz), 83.9 (d, *J* = 2.8 Hz), 73.6, 52.9 (d, *J* = 16.4 Hz), 46.9 (d, *J* = 5.7 Hz), 46.1, 38.8, 29.3 (d, *J* = 2.9 Hz), 27.1, 23.9, 23.1, 21.2, 20.7. ^{19}F NMR (376 MHz, $CDCl_3$) δ -126.98 (ddd, *J* = 292.1, $J_{H\text{-}F}$ = 58.9, 53.5 Hz). IR (KBr cm^{-1}): 3081, 2957, 2884, 1463, 1391, 1189, 1089, 1022, 974,

820. (TOF MS) m/z (%): $[(M-C_4H_7OH)+H]^+$ calcd for $C_{10}H_{15}F$ 155.1231; found: 155.1230.

***(1R,4S)-2-(2-Ethoxyethoxy)-1-(fluoromethyl)-7,7-dimethylbicyclo[2.2.1]heptane* (4i):**

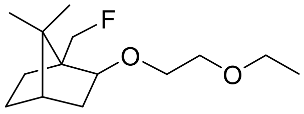

Colorless oil (112 mg, 94%). $[\alpha]_D$ = -2.42 (c = 1.24, EtOH). ^{1}H NMR (101 MHz, $CDCl_3$) δ 4.73 (dd, *J* = 47.2, 9.2 Hz, 1H), 4.41 (dd, *J* = 48.2, 9.2 Hz, 1H), 3.62 – 3.40 (m, 7H), 1.88– 1.56 (m, 6H), 1.17 (t, *J* = 7.0 Hz, 4H), 1.05 (s, 3H), 0.89 (s, 3H). ^{13}C NMR (101 MHz, $CDCl_3$) δ 84.7 (d, *J* = 2.5 Hz), 83.9 (d, *J* = 162.9 Hz), 70.1, 69.0, 66.7, 52.9 (d, *J* = 16.8 Hz), 46.1, 38.6, 29.20, 29,17, 27.0, 21.2, 20.6, 15.4. ^{19}F NMR (376 MHz, $CDCl_3$) δ -126.86 (ddd, *J* = 353.6, 292.7, J_{H-F} = 60.3, 57.4 Hz). IR (KBr cm^{-1}): 2950, 2882, 1463, 1404, 1188, 1105, 977, 843. (TOF MS) m/z (%): $[(M-C_4H_{10}O_2)+H]^+$ calcd for $C_{10}H_{15}F$ 155.1231; found: 155.1231.

***(1R,4S)-2-(Cyclohexyloxy)-1-(fluoromethyl)-7,7-dimethylbicyclo[2.2.1]heptane* (4j):**

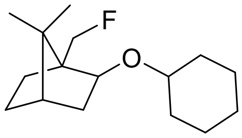

Colorless oil (76 mg, 60%). $[\alpha]_D$ = -3.42 (c = 1.49, EtOH). ^{1}H NMR (101 MHz, $CDCl_3$) δ 4.74 (dd, *J* = 47.3, 9.1 Hz, 1H), 4.41 (dd, *J* = 48.2, 9.1 Hz, 1H), 3.54 (dd, *J* = 7.7, 3.5 Hz, 1H), 3.29-3.20 (m, 1H), 1.83 – 1.65 (m, 10H), 1.31 – 1.21 (m, 7H), 1.09 (s, 3H), 0.90 (s, 3H). ^{13}C NMR (101 MHz, $CDCl_3$) δ 84.3 (d, *J* = 162.5 Hz), 81.9 (d, *J* = 2.9 Hz), 76,6, 52.8 (d, *J* = 16.5 Hz), 47.0 (d, *J* = 6.3 Hz), 46,4, 40.2, 33.6, 32.1, 29.3 (d, *J* = 2.8 Hz), 27.2, 26.1, 24.00, 23.98, 21.3, 20.7. ^{19}F NMR (376 MHz, $CDCl_3$) δ -127.26 (ddd, *J* = 290.5, 60.0, 54.7 Hz). IR (KBr cm^{-1}): 2933, 2891, 2864, 1733, 1457, 1393, 1374, 1186, 1080, 979. (TOF MS) m/z (%): $(M+Na)^+$ calcd for $C_{16}H_{27}FO$ 277.1938; found: 277.1936.

References

Balci, M. (2005). *Basic ^{1}H-and ^{13}C-NMR spectroscopy* (pp. 130-132). Elsevier.

Daştan, A., Taşkesenligil, Y., Tümer, F., & Balci, M. (1996). High temperature bromination VIII: Bromination of homobenzonorbornadiene. *Tetrahedron*, *52*(44), 14005-14020.

Gültekin, D. D., Daştan, A., Taşkesenligil, Y., Kazaz, C., Zorlu, Y., & Balci, M. (2023). Bromination of endo-7-norbornene derivatives revisited: failure of a computational NMR method in elucidating the configuration of an organic structure. *Beilstein Journal of Organic Chemistry*, *19*(1), 764-770.

Moreno-Dorado, F. J., Guerra, F. M., Manzano, F. L., Aladro, F. J., Jorge, Z. D., & Massanet, G. M. (2003). CeCl3/NaClO: a safe and efficient reagent for the allylic chlorination of terminal olefins. *Tetrahedron letters*, *44*(35), 6691-6693.

Wakchaure, V. N., DeSnoo, W., Laconsay, C. J., Leutzsch, M., Tsuji, N., Tantillo, D. J., & List, B. (2024). Catalytic asymmetric cationic shifts of aliphatic hydrocarbons. *Nature*, *625*(7994), 287-292.

Zyk, N. V., Beloglazkina, E. K., Tyurin, V. S., & Zefirov, N. S. (1998). A NEW METHOD FOR MIXED HALOGENATION. N-CHLOROAMINE-PHOSPHORUS BROMIDE SYSTEM AS A SYNTHETIC EQUIVALENT OF THE MIXED HALOGEN Cl+ Br−. *Phosphorus, Sulfur, and Silicon and the Related Elements*, *139*(1), 107-122.

^{1}H NMR, ^{13}C NMR, ^{19}F NMR, and HRMS Spectra

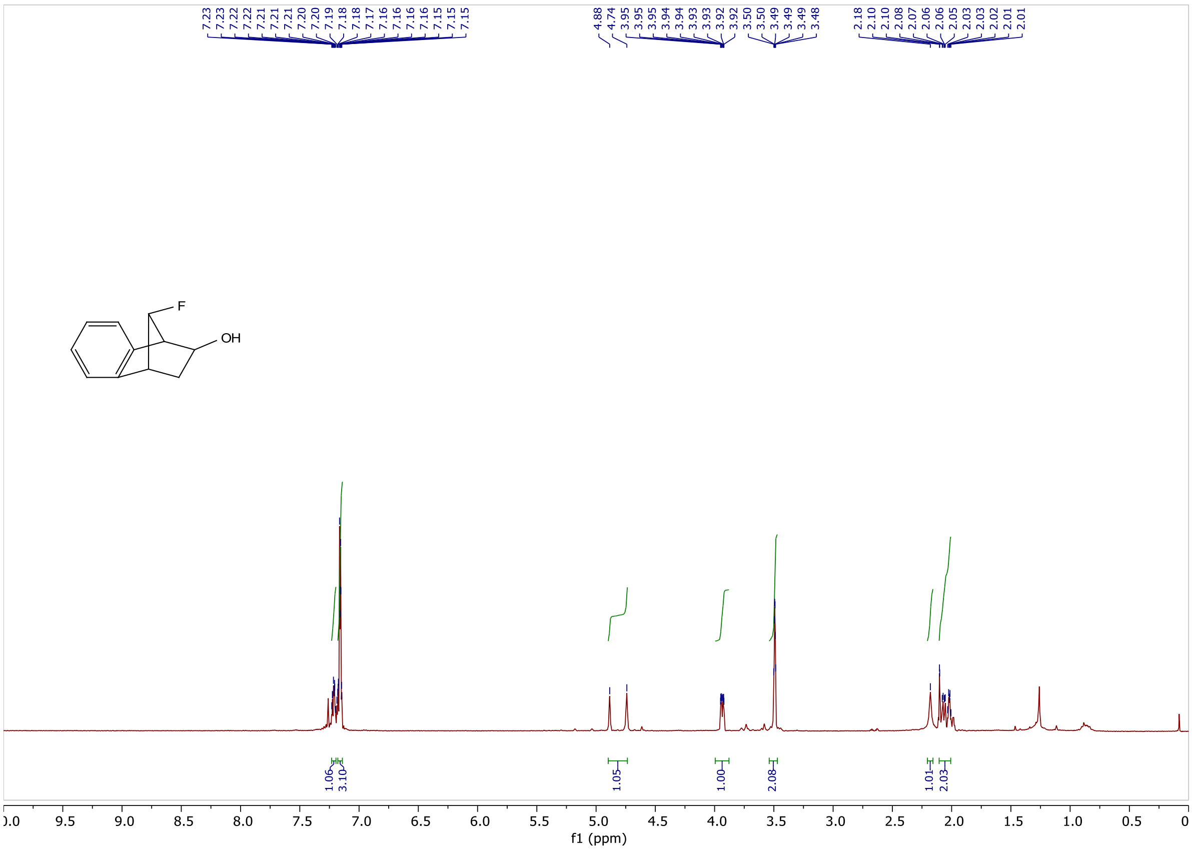

400 MHz ^{1}H-NMR spectrum of **3a** ($CDCl_3$)

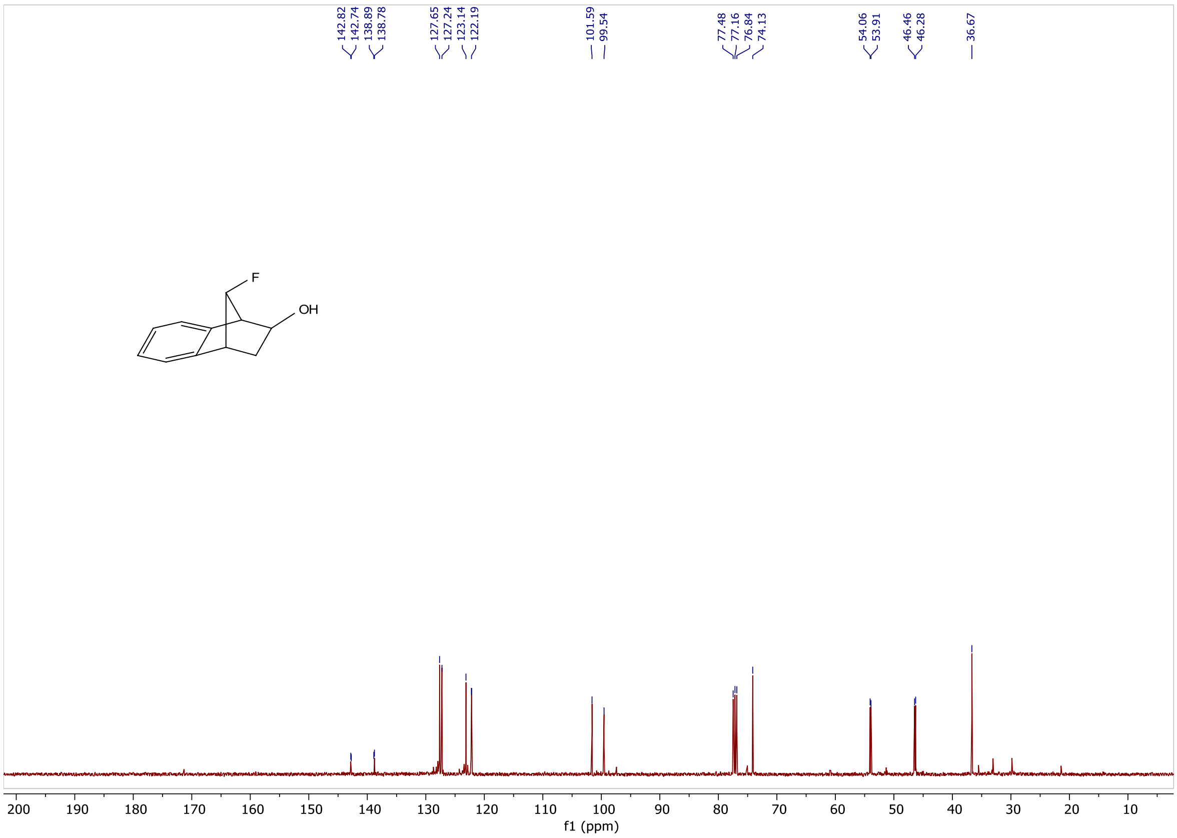

101 MHz ^{13}C-NMR spectrum of **3a** ($CDCl_3$)

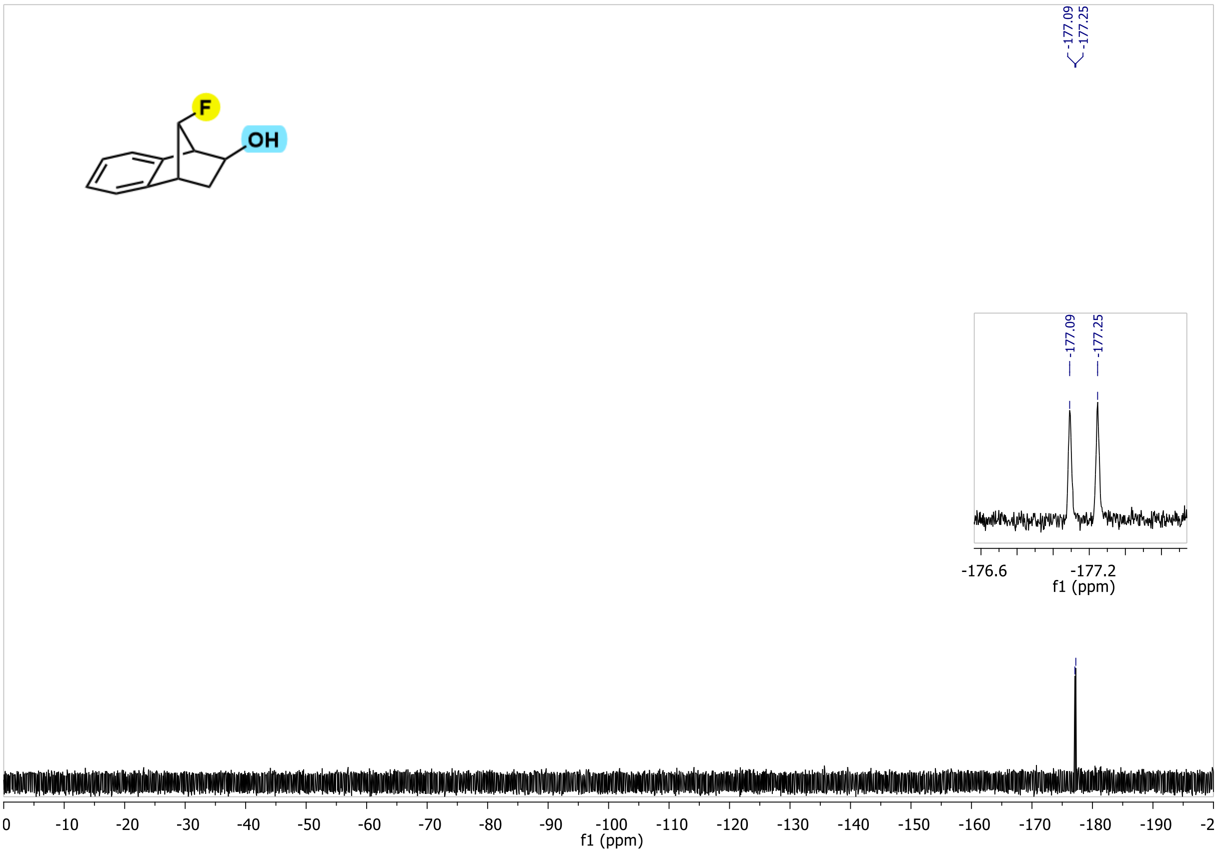

376 MHz ^{19}F-NMR spectrum of **3a** ($CDCl_3$)

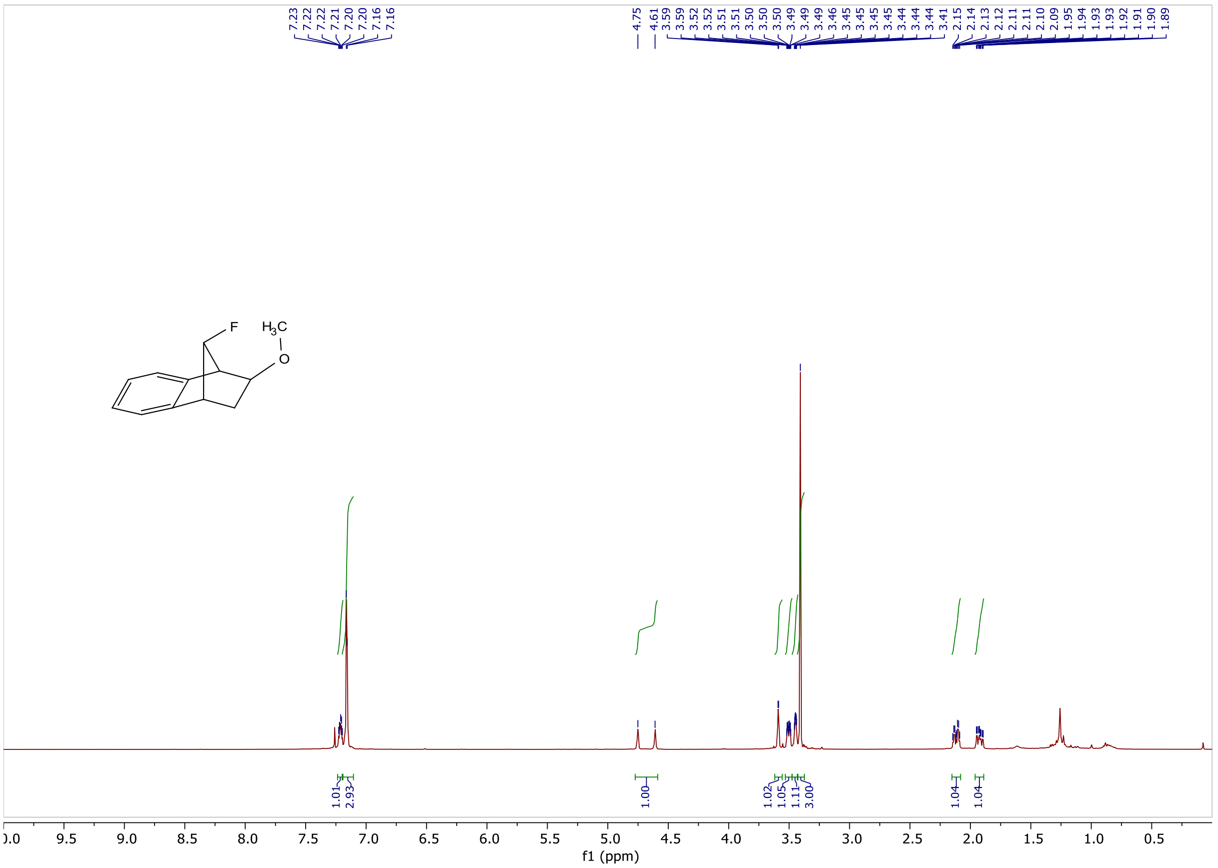

400 MHz ^{1}H-NMR spectrum of **3b** ($CDCl_3$)

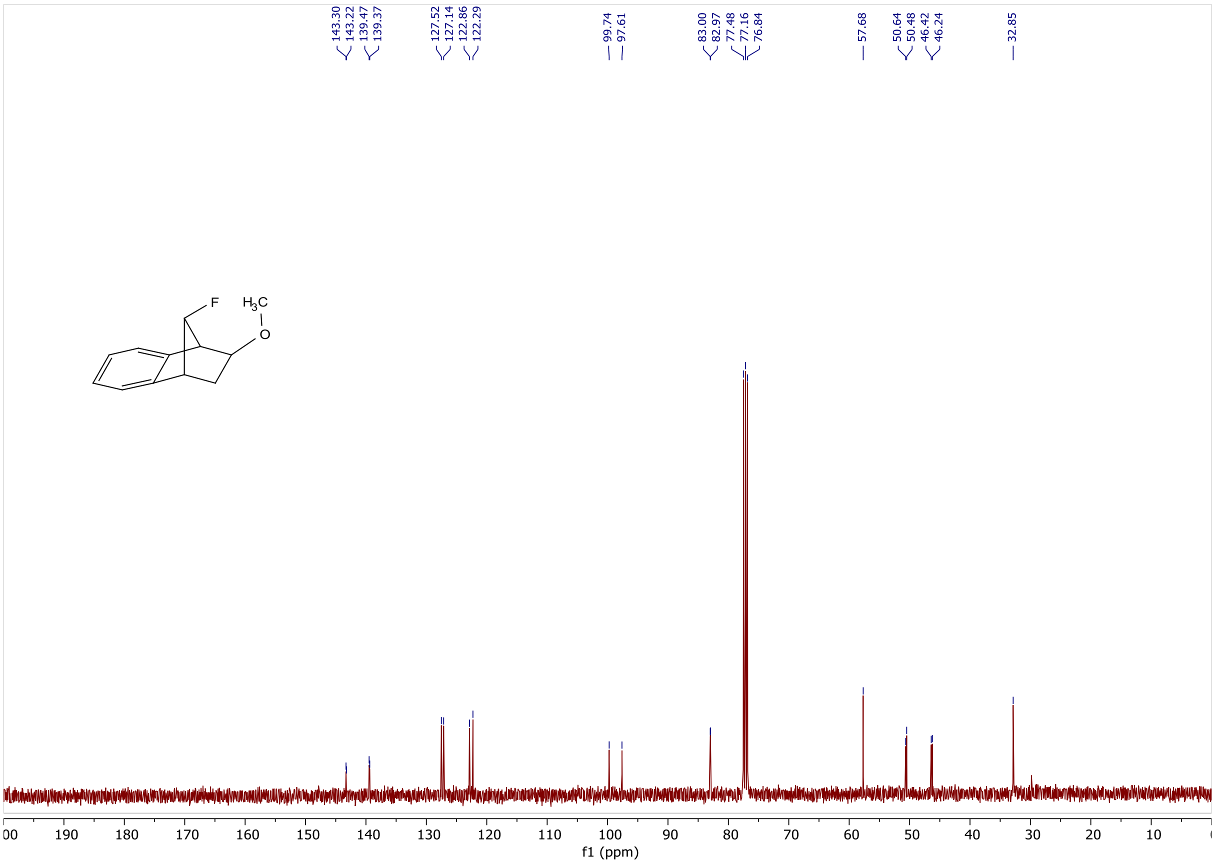

101 MHz ^{13}C-NMR spectrum of **3b** ($CDCl_3$)

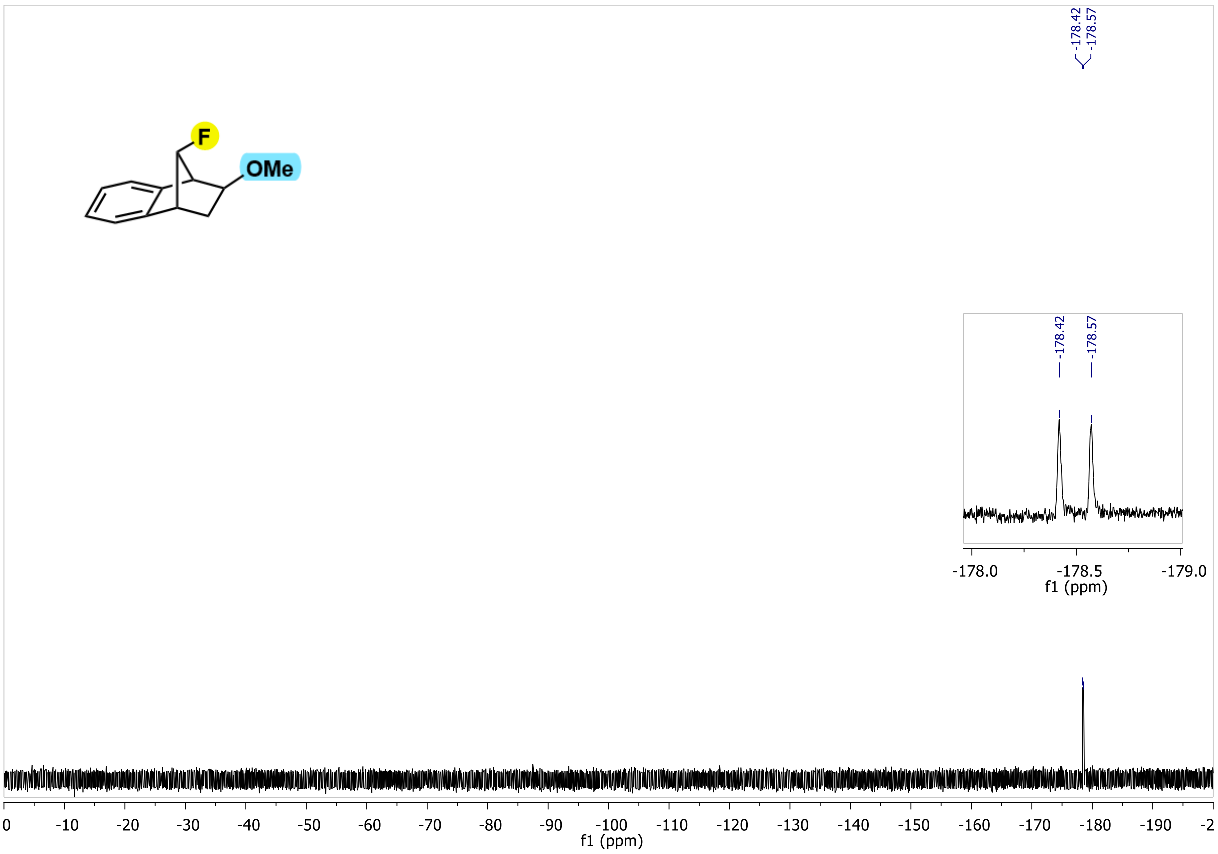

376 MHz ^{19}F-NMR spectrum of **3b** ($CDCl_3$)

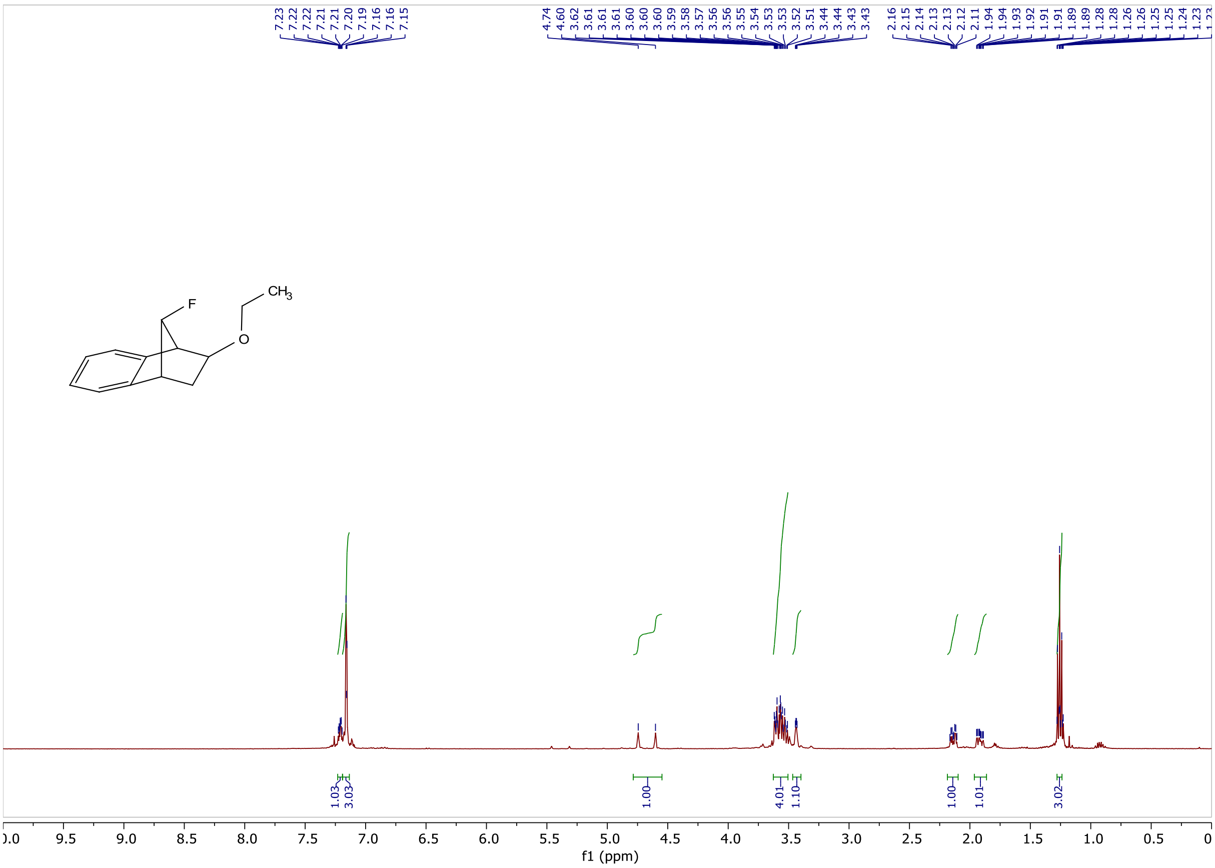

400 MHz ^{1}H-NMR spectrum of **3c** ($CDCl_3$)

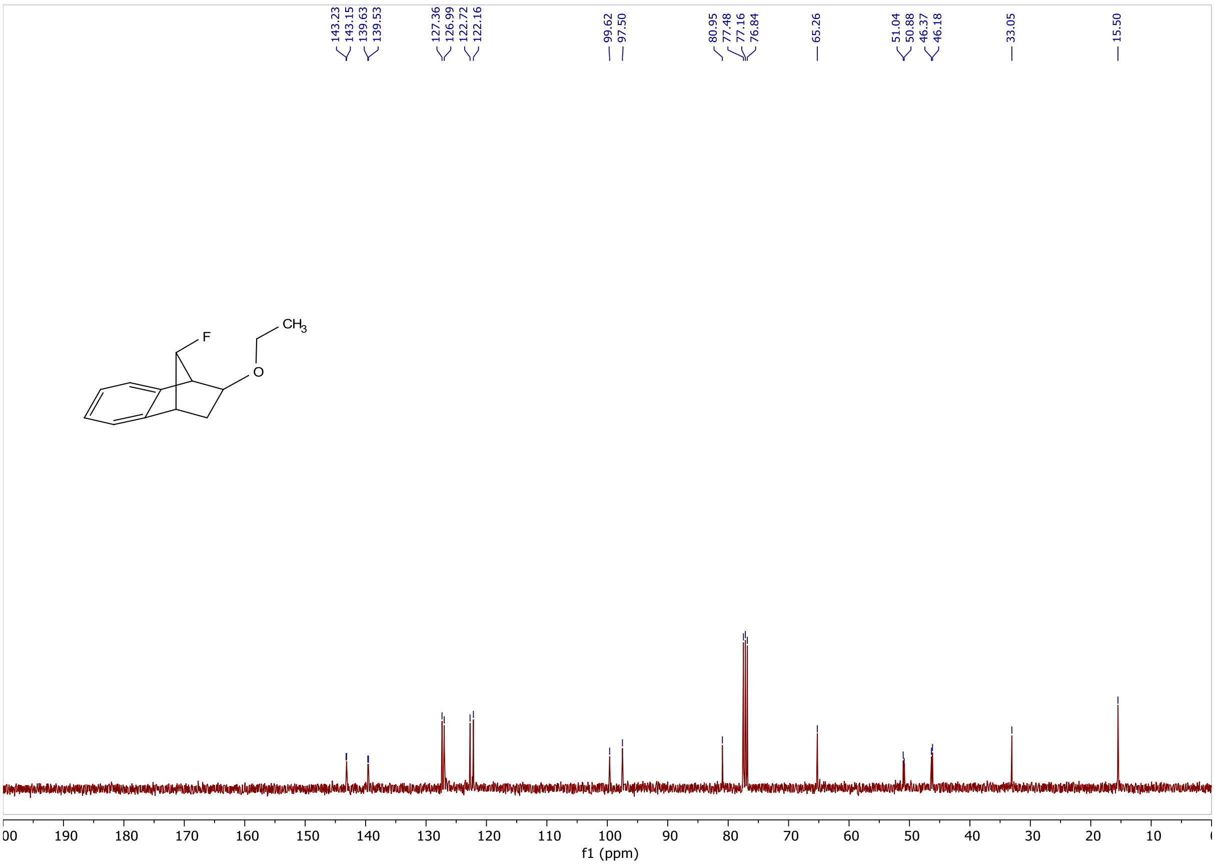

101 MHz ^{13}C-NMR spectrum of **3c** ($CDCl_3$)

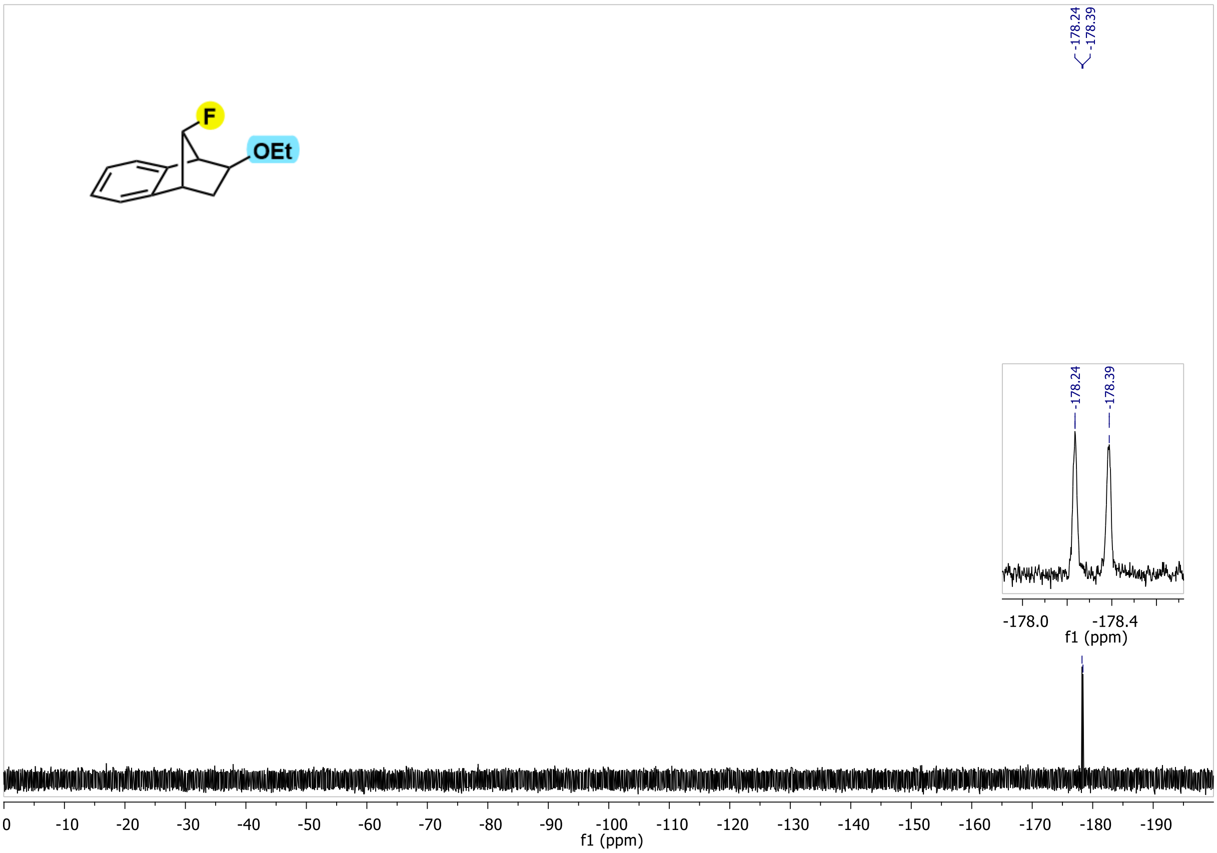

376 MHz ^{19}F-NMR spectrum of **3c** ($CDCl_3$)

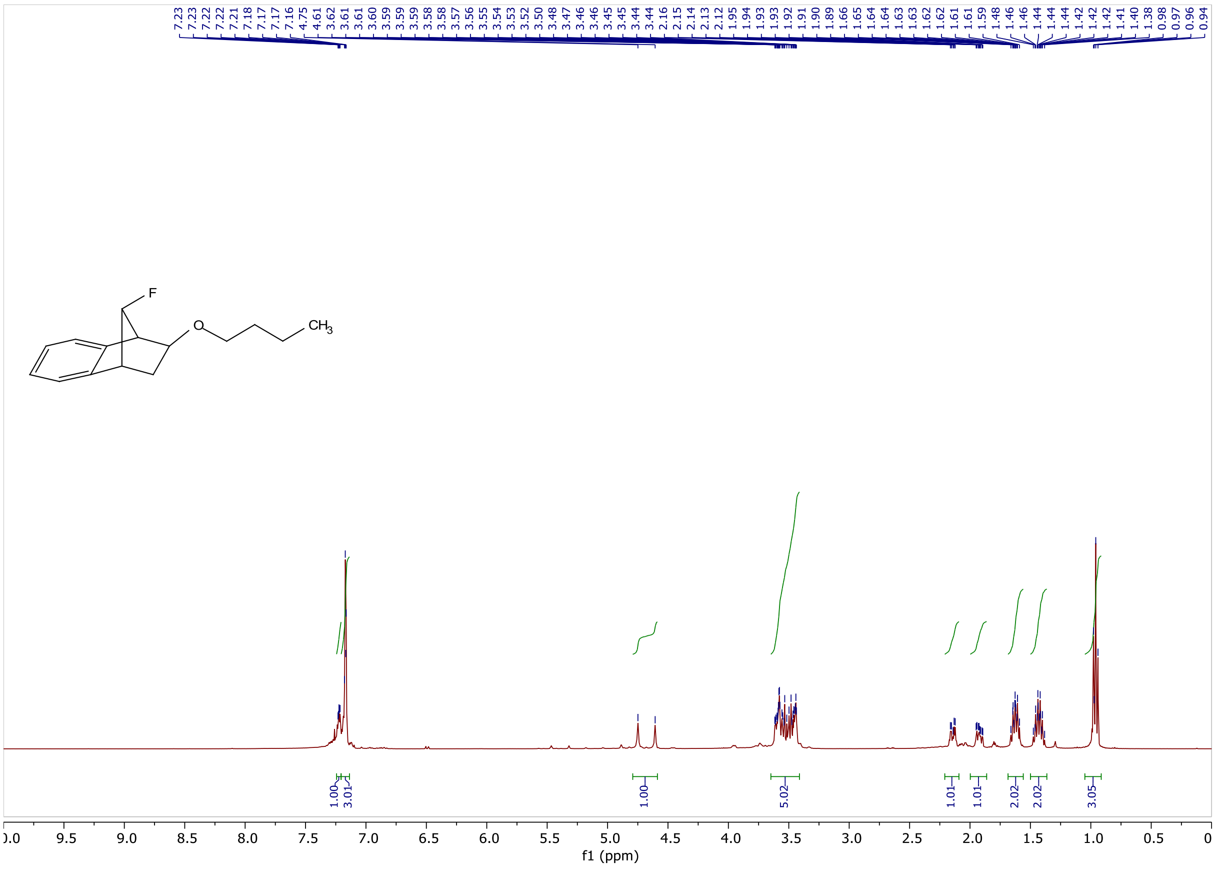

400 MHz ^{1}H-NMR spectrum of **3d** ($CDCl_3$)

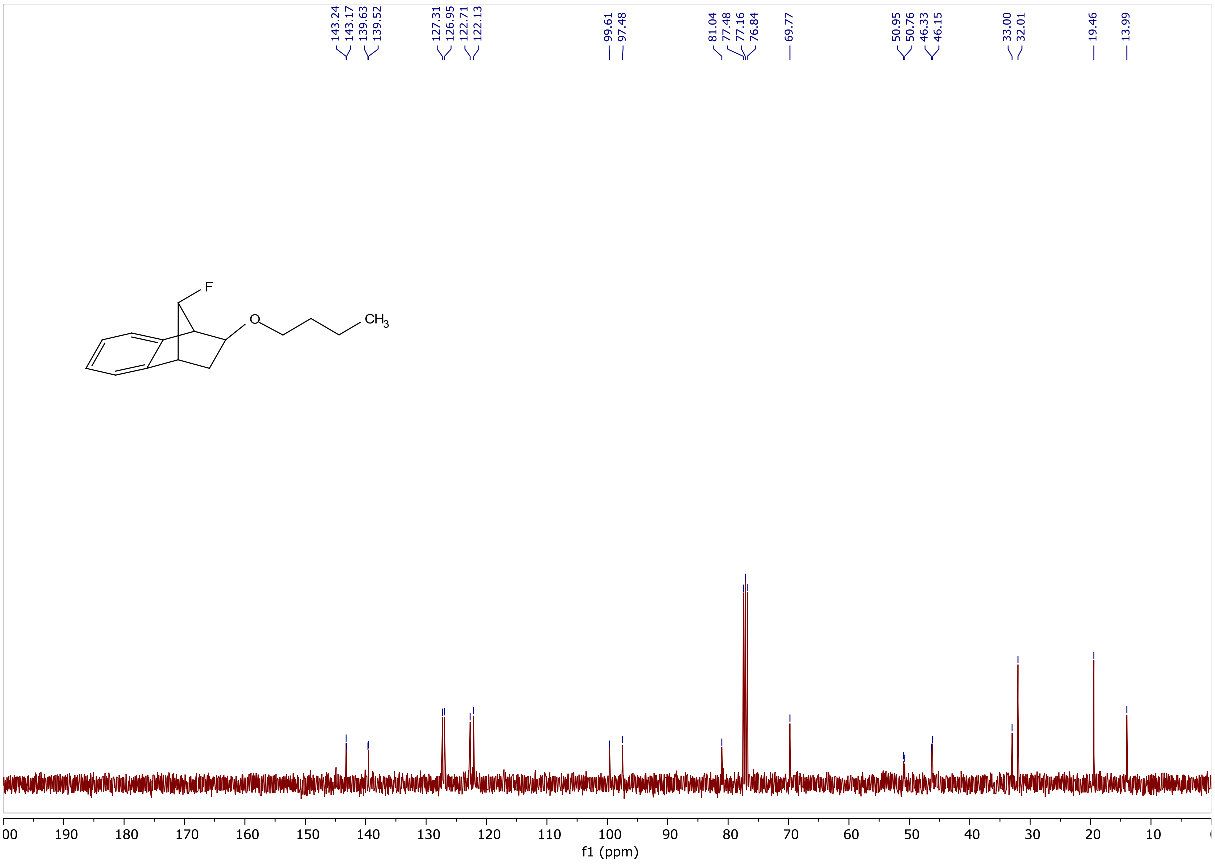

101 MHz ^{13}C-NMR spectrum of **3d** ($CDCl_3$)

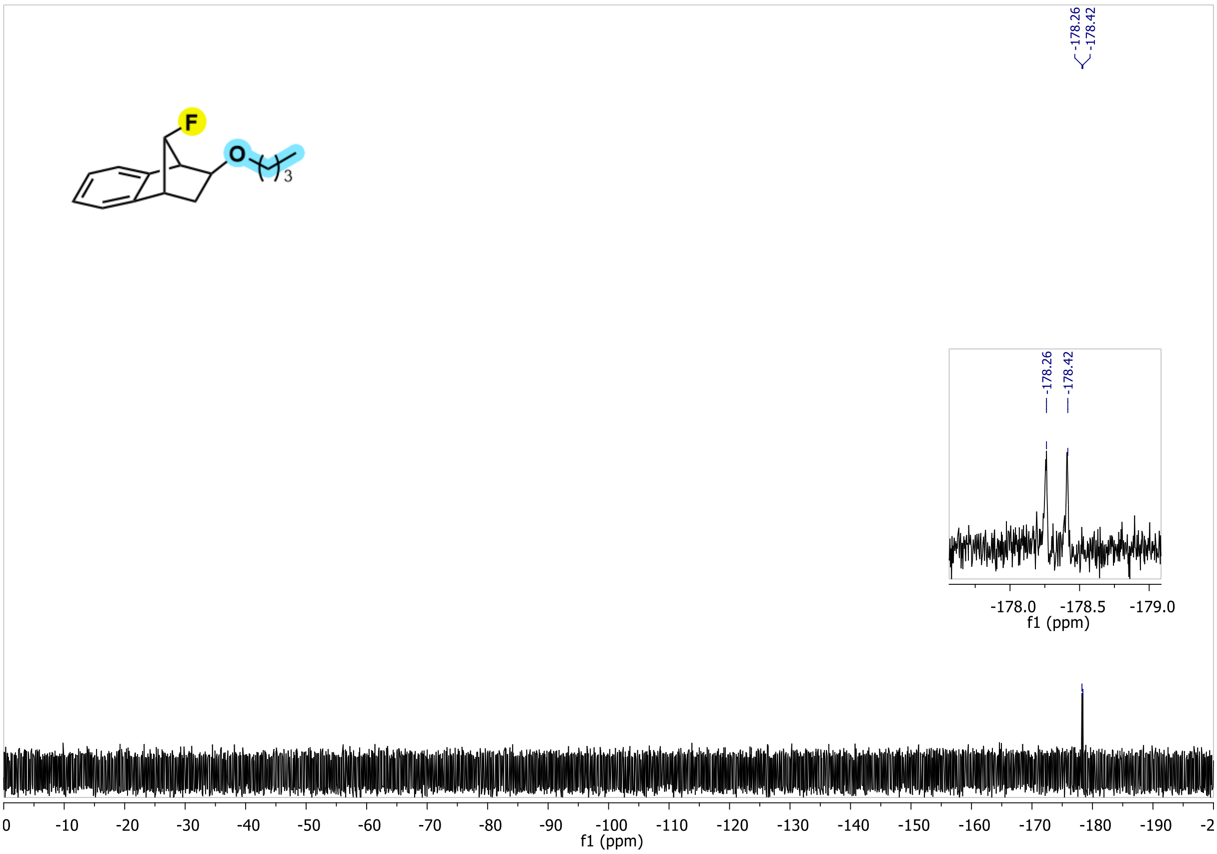

376 MHz ^{19}F-NMR spectrum of **3d** ($CDCl_3$)

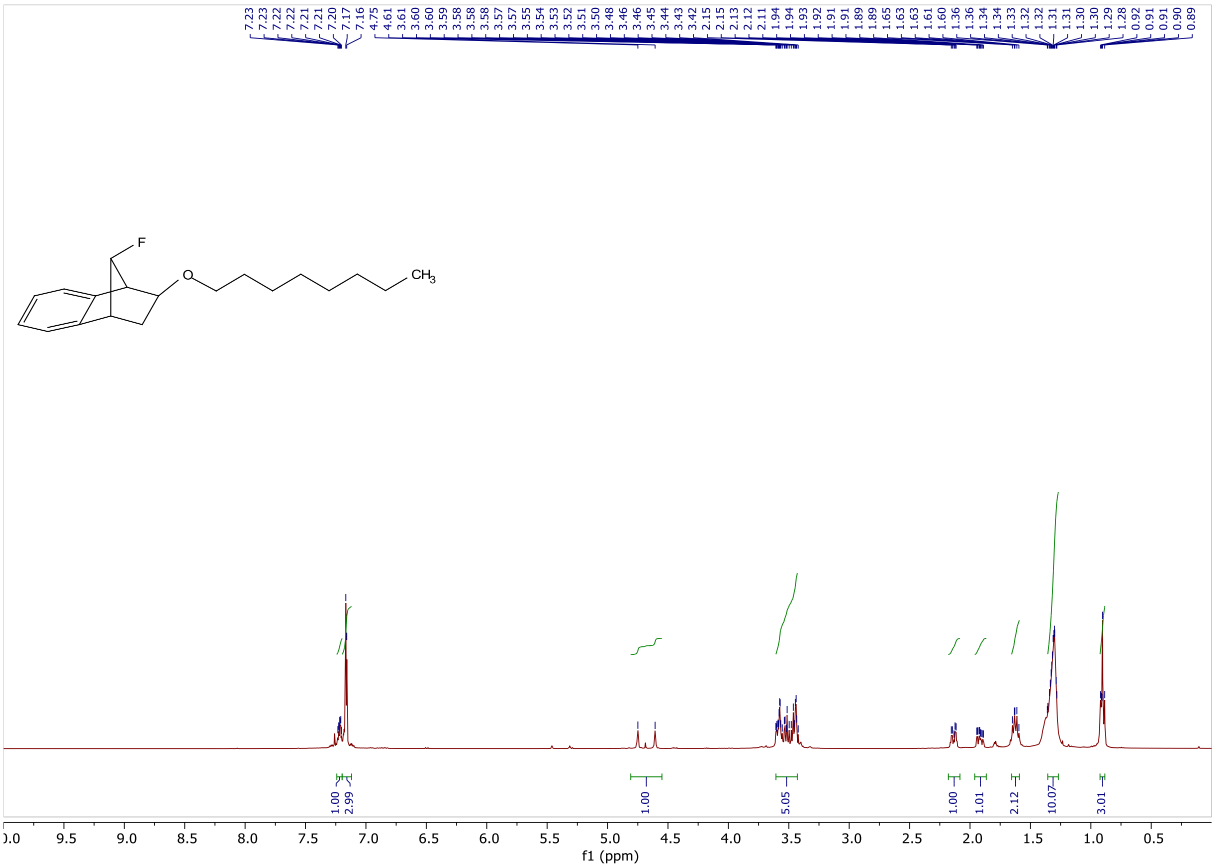

400 MHz ^{1}H-NMR spectrum of **3e** ($CDCl_3$)

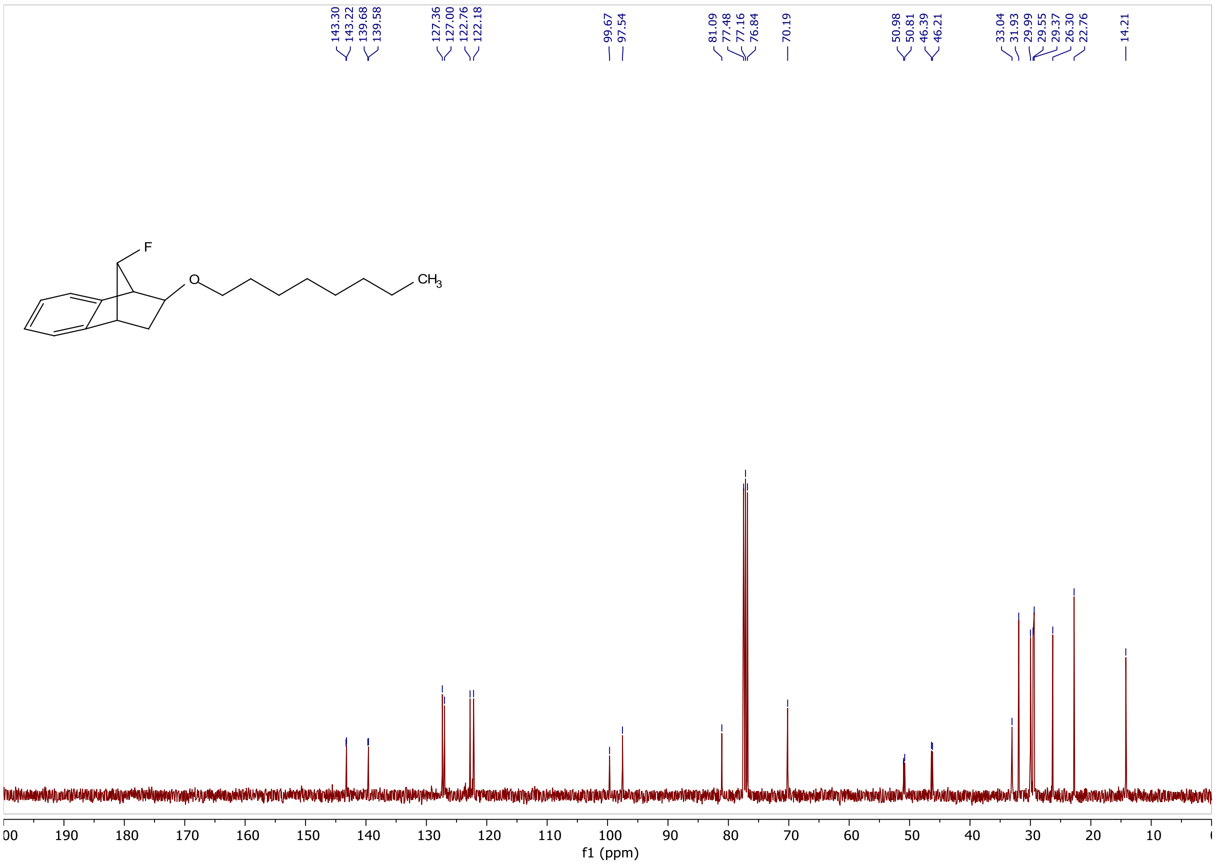

101 MHz ^{13}C-NMR spectrum of **3e** ($CDCl_3$)

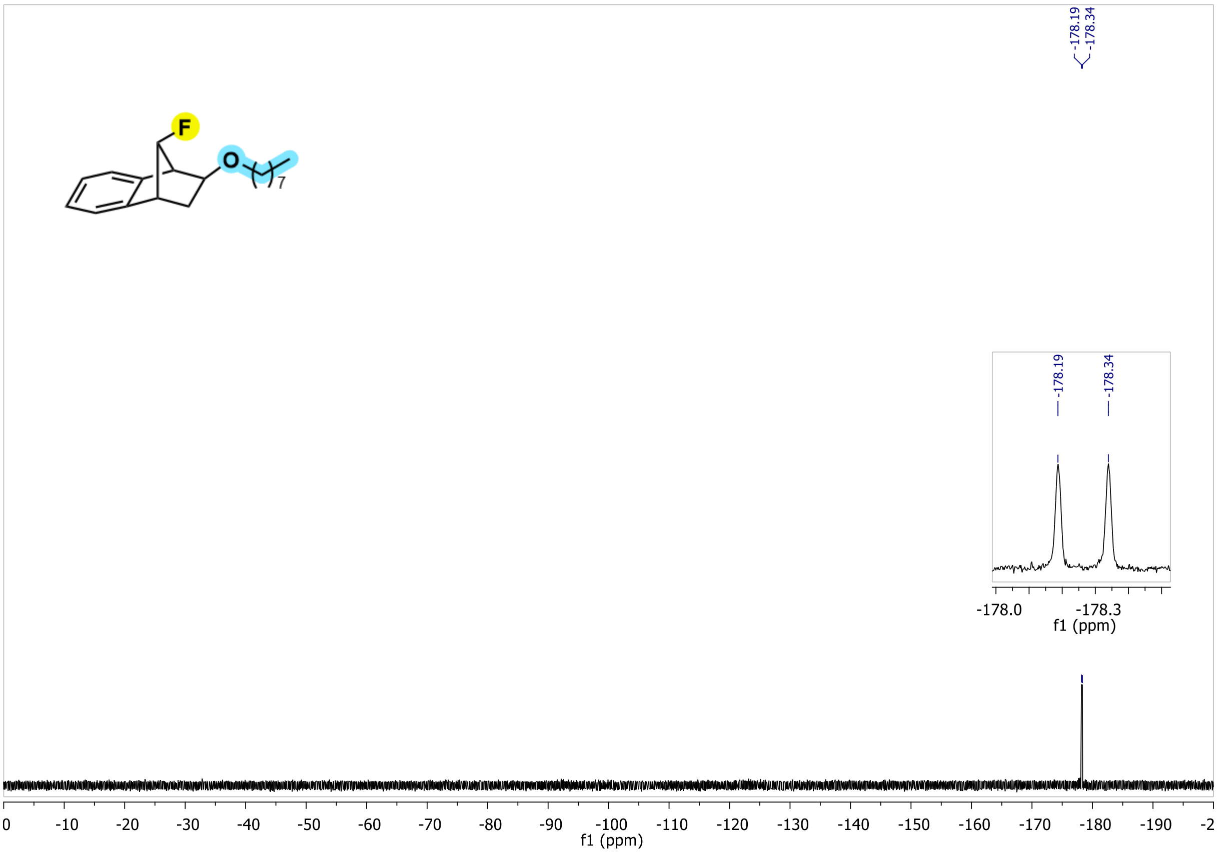

376 MHz ^{19}F-NMR spectrum of **3e** ($CDCl_3$)

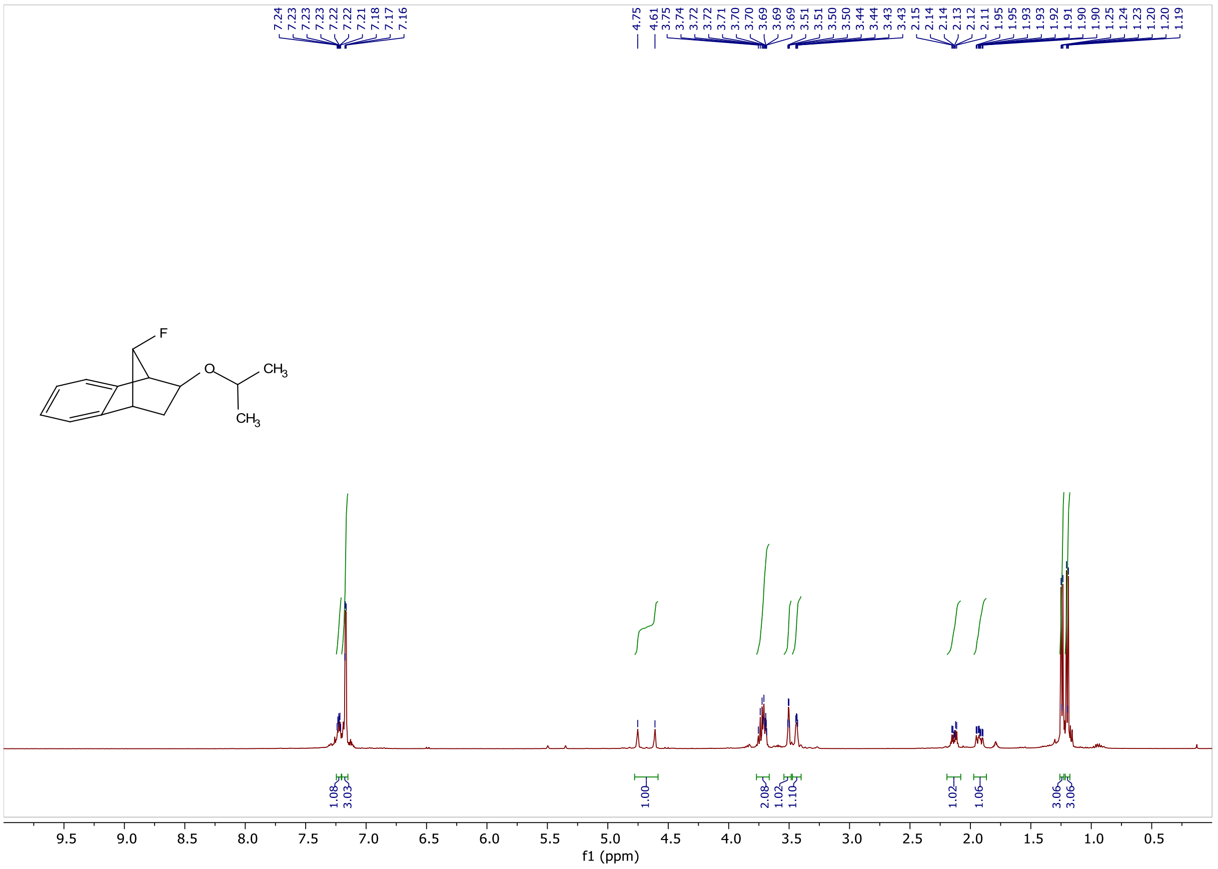

400 MHz ^{1}H-NMR spectrum of **3f** ($CDCl_3$)

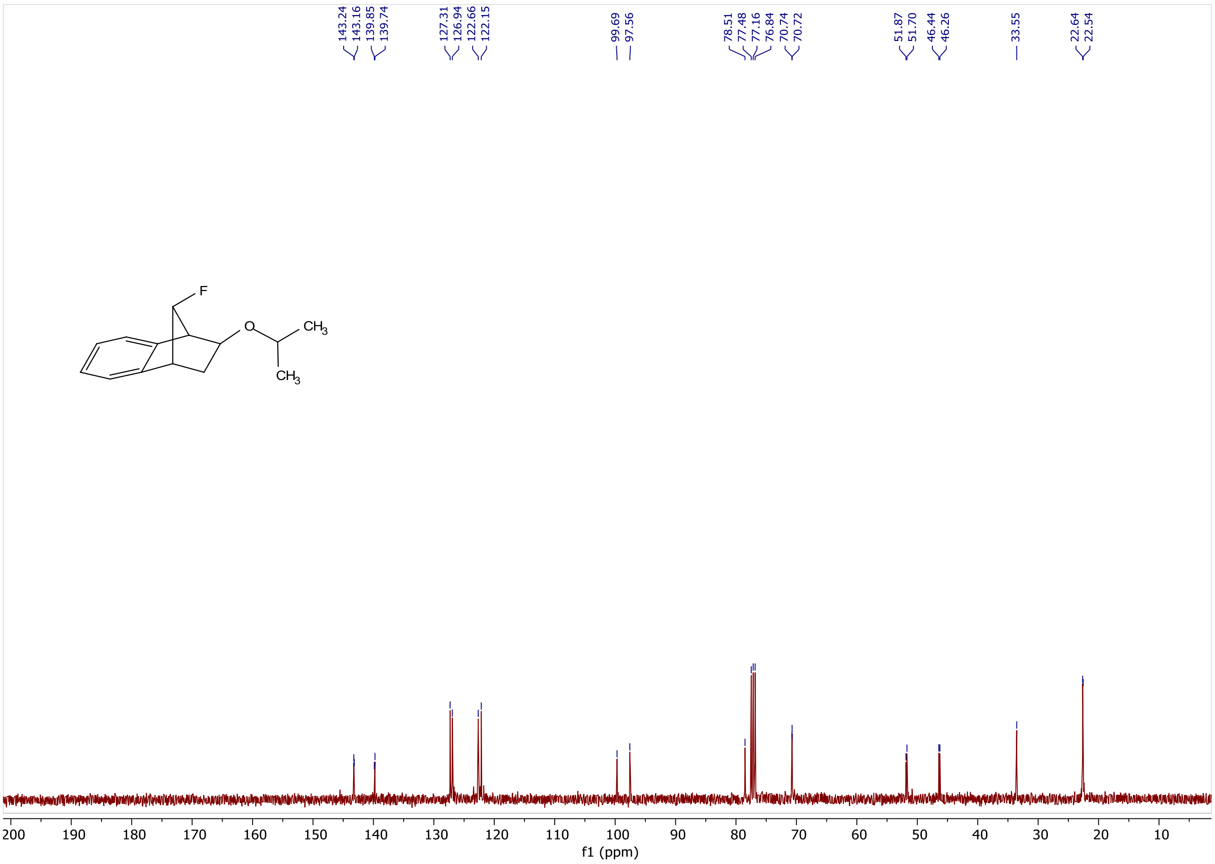

101 MHz ^{13}C-NMR spectrum of **3f** ($CDCl_3$)

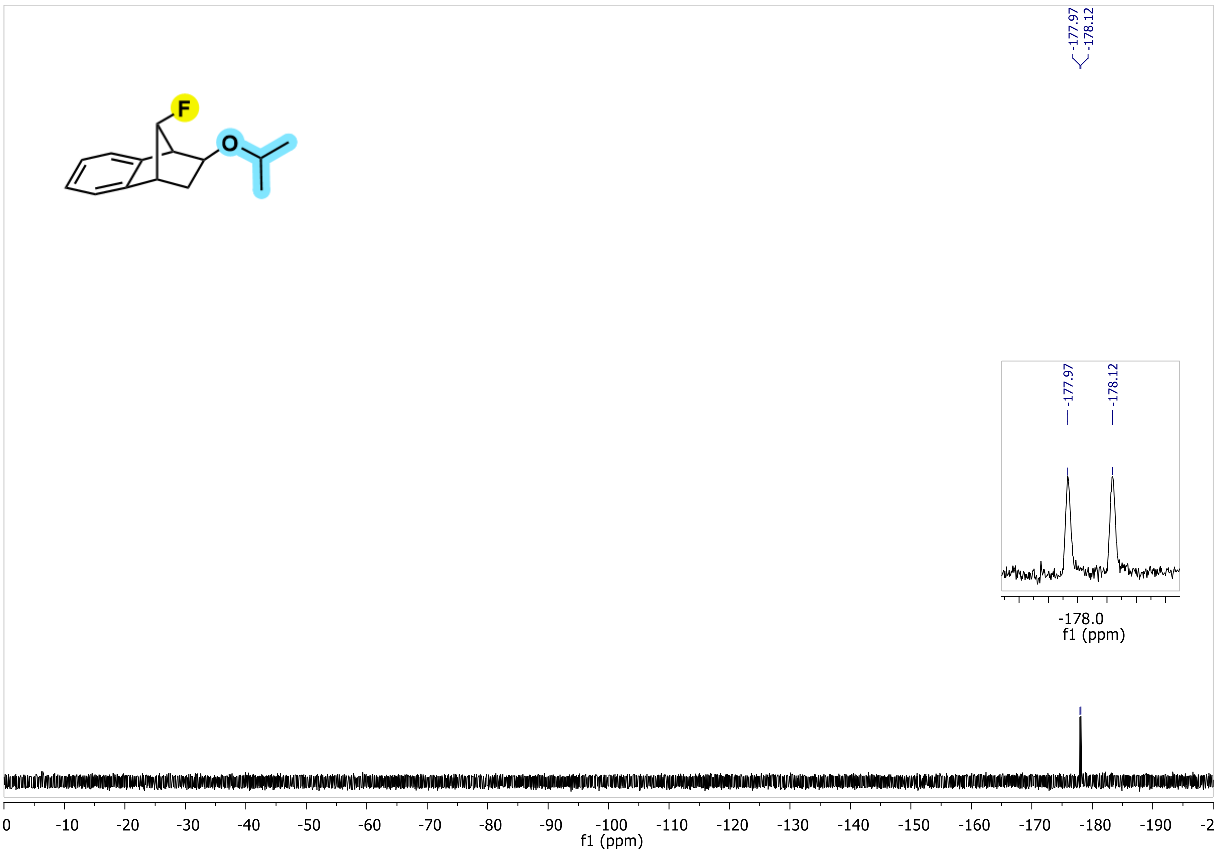

376 MHz ^{19}F-NMR spectrum of **3f** ($CDCl_3$)

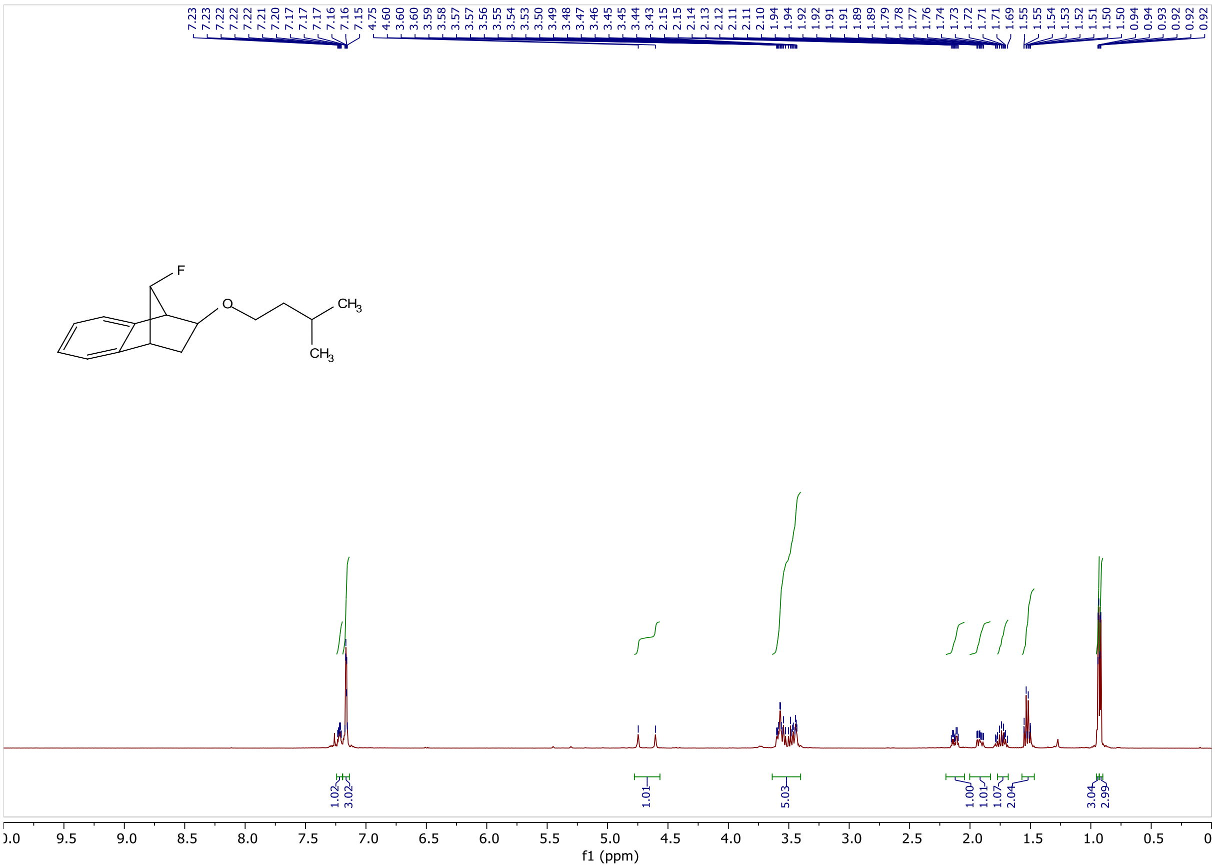

400 MHz ^{1}H-NMR spectrum of **3g** ($CDCl_3$)

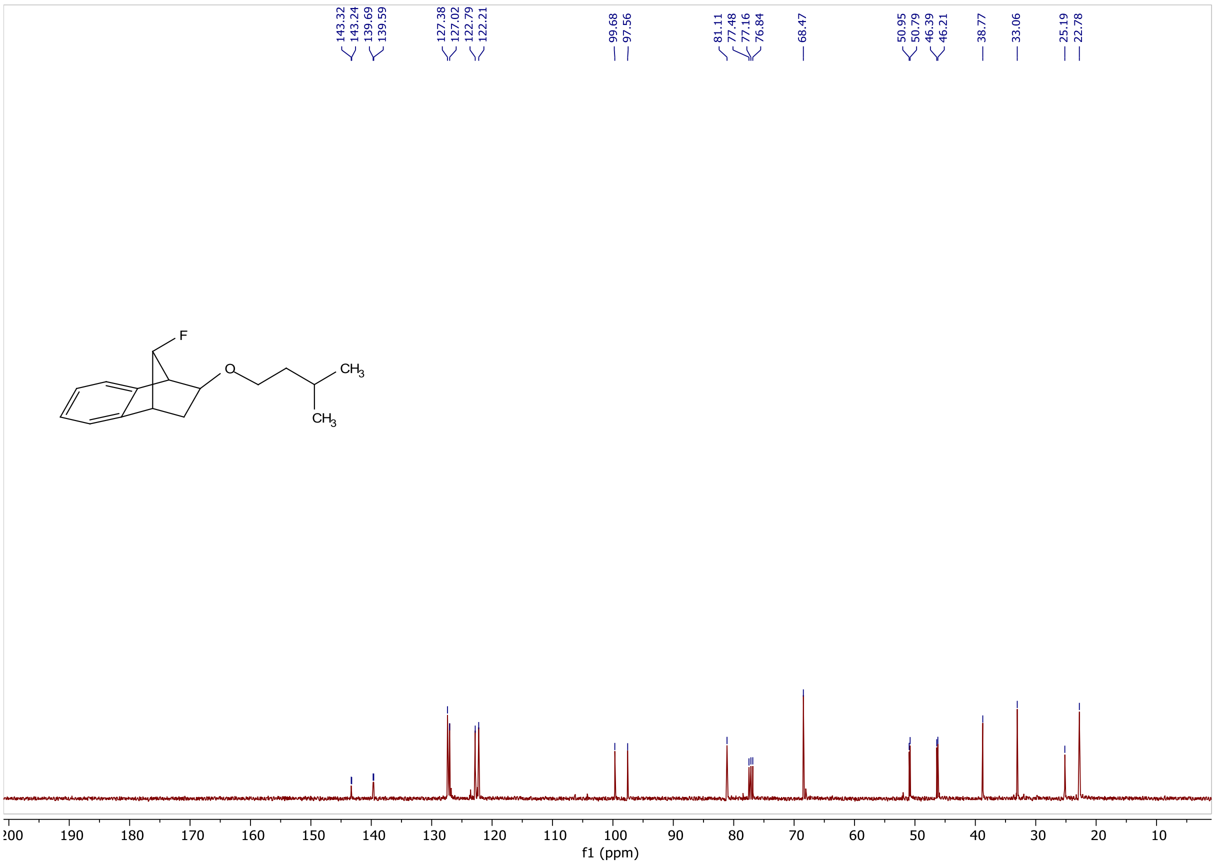

101 MHz ^{13}C-NMR spectrum of **3g** ($CDCl_3$)

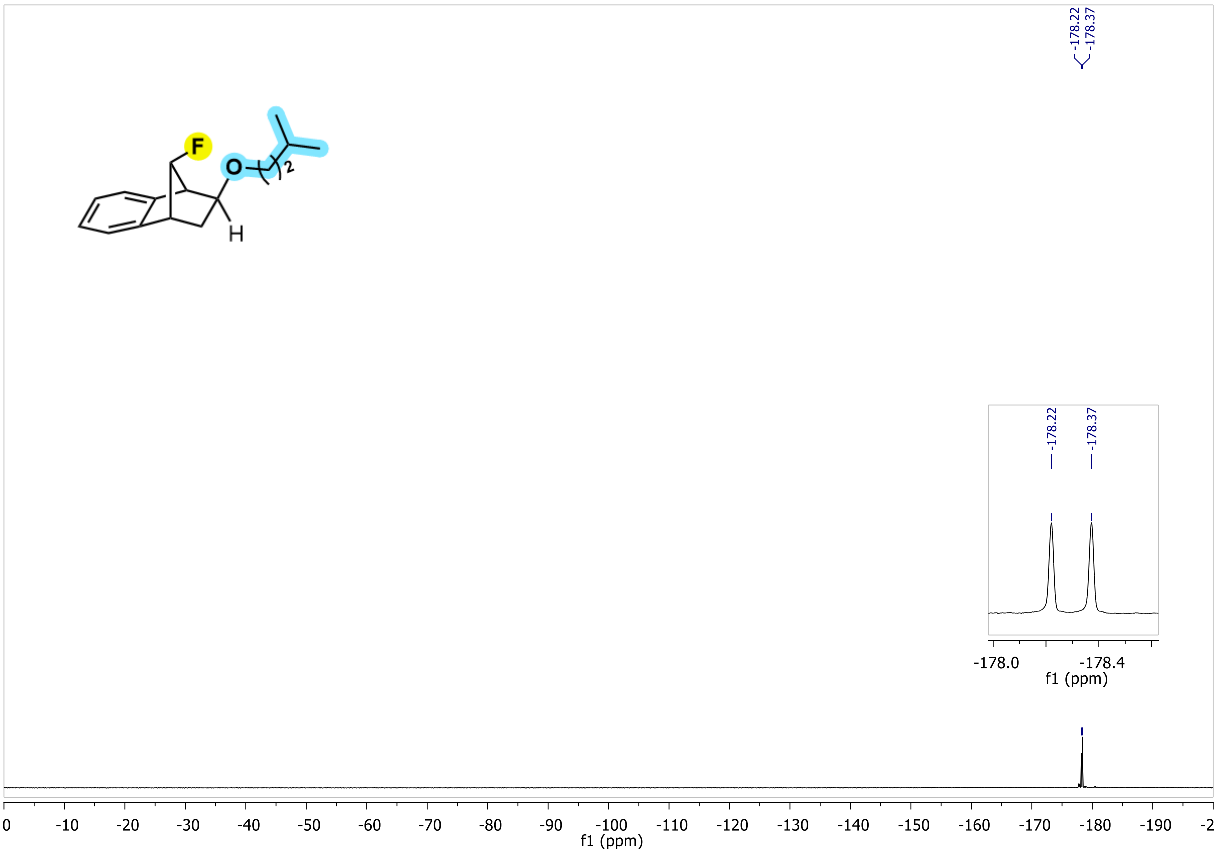

376 MHz ^{19}F-NMR spectrum of **3g** ($CDCl_3$)

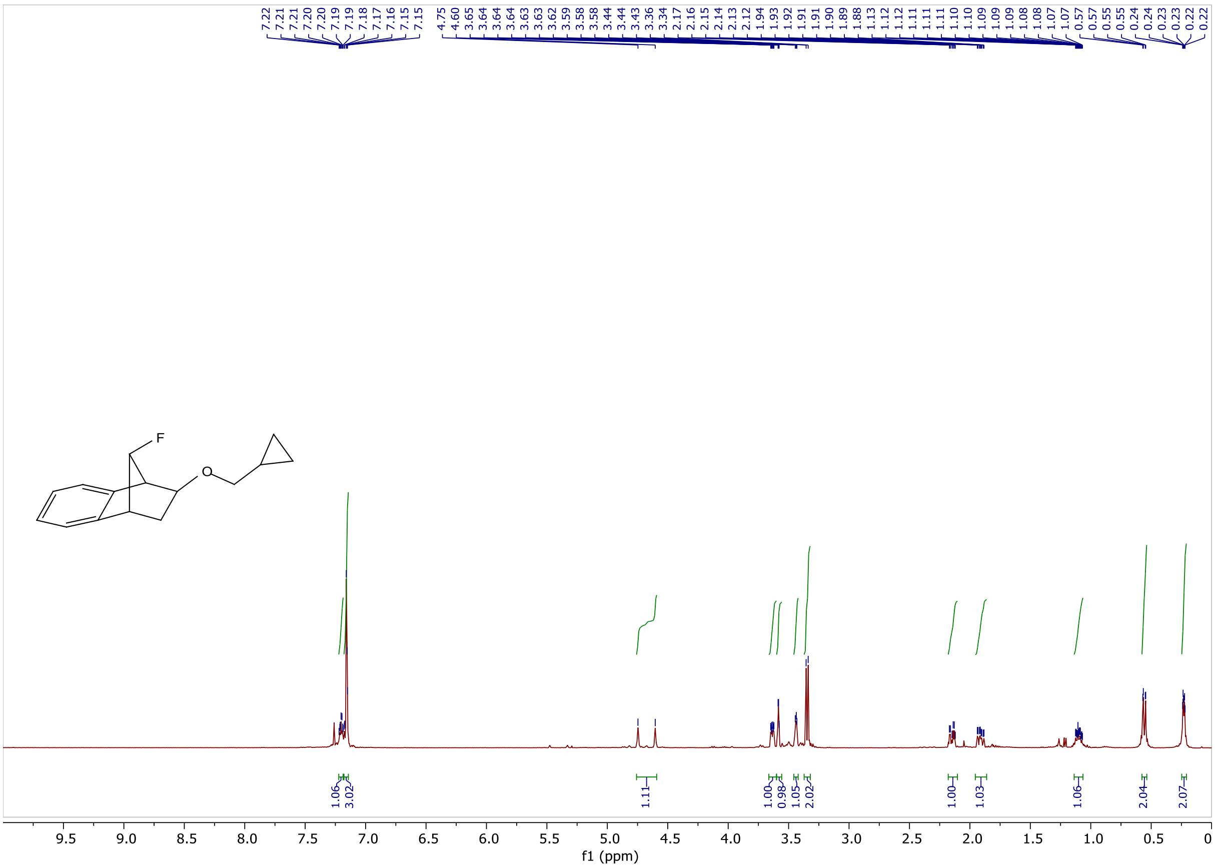

400 MHz ^{1}H-NMR spectrum of **3h** ($CDCl_3$)

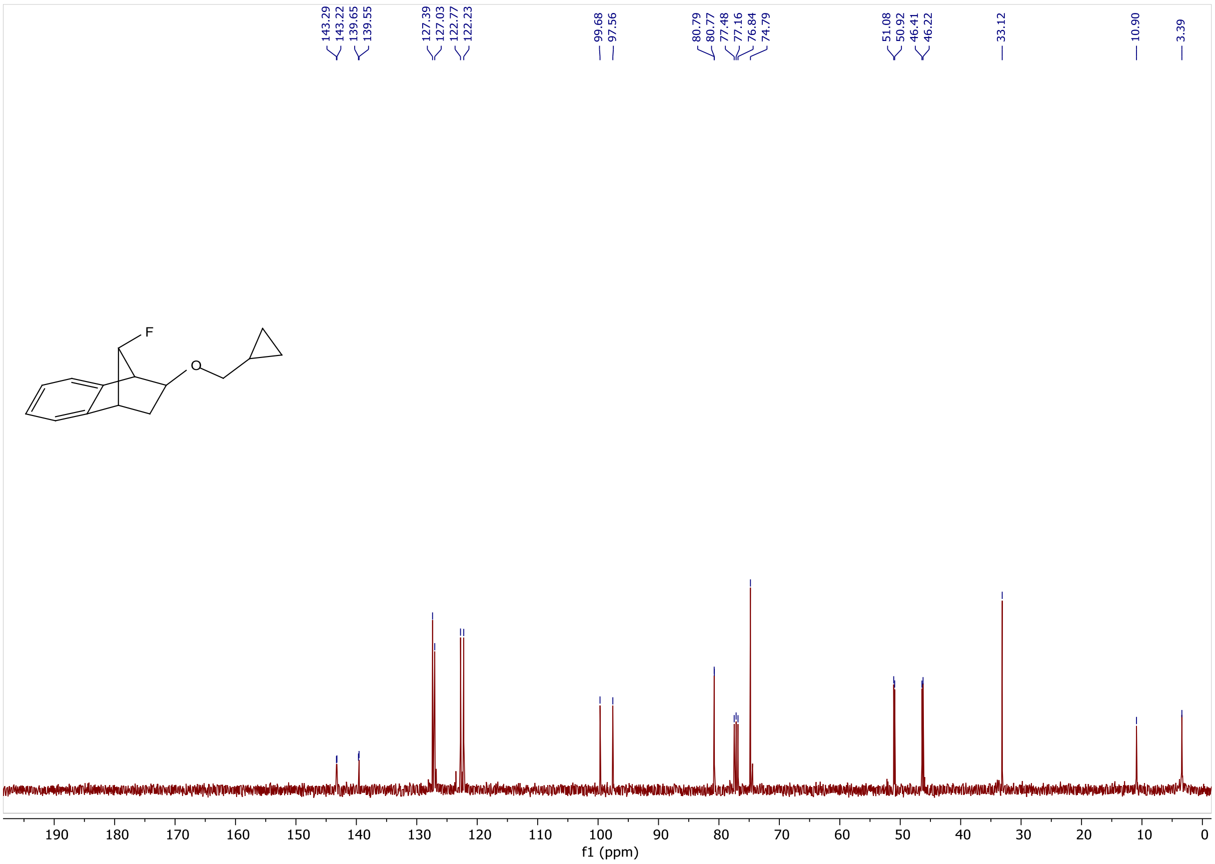

101 MHz ^{13}C-NMR spectrum of **3h** ($CDCl_3$)

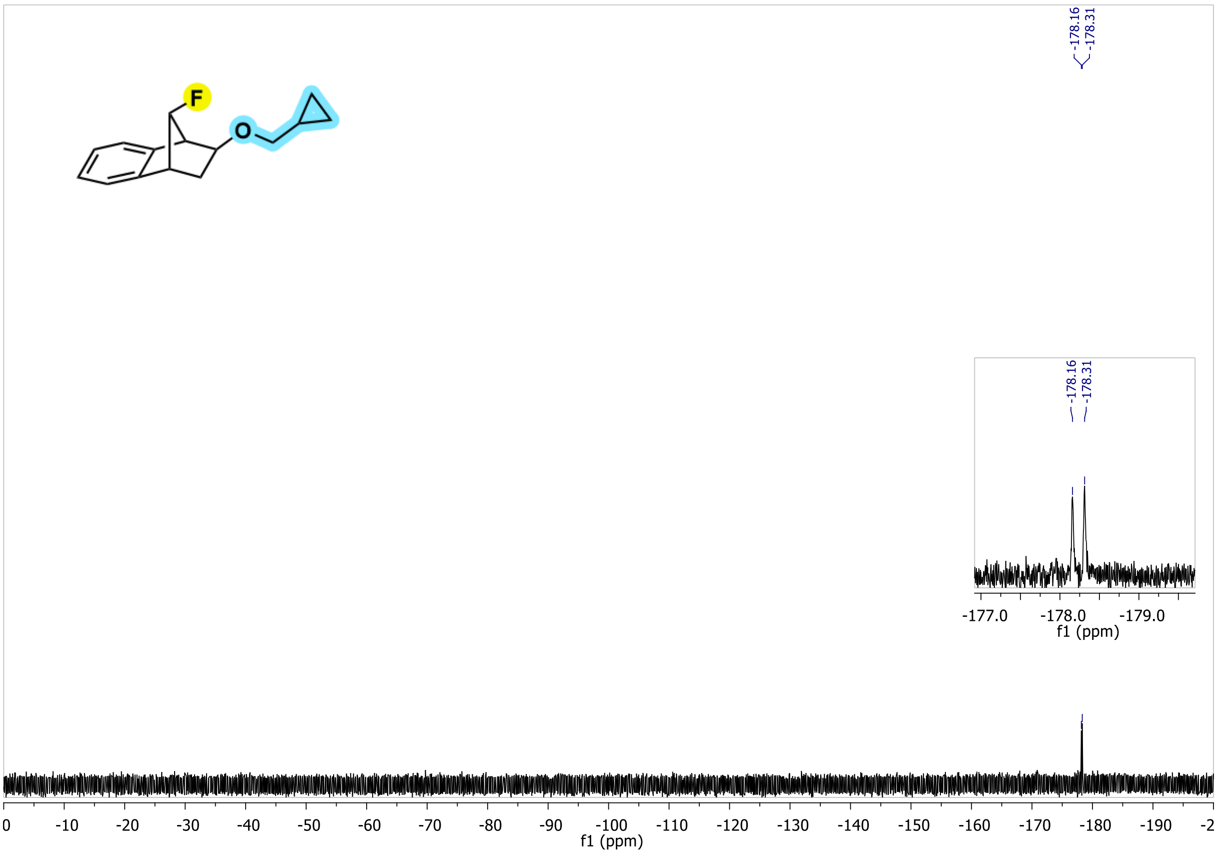

376 MHz ^{19}F-NMR spectrum of **3h** ($CDCl_3$)

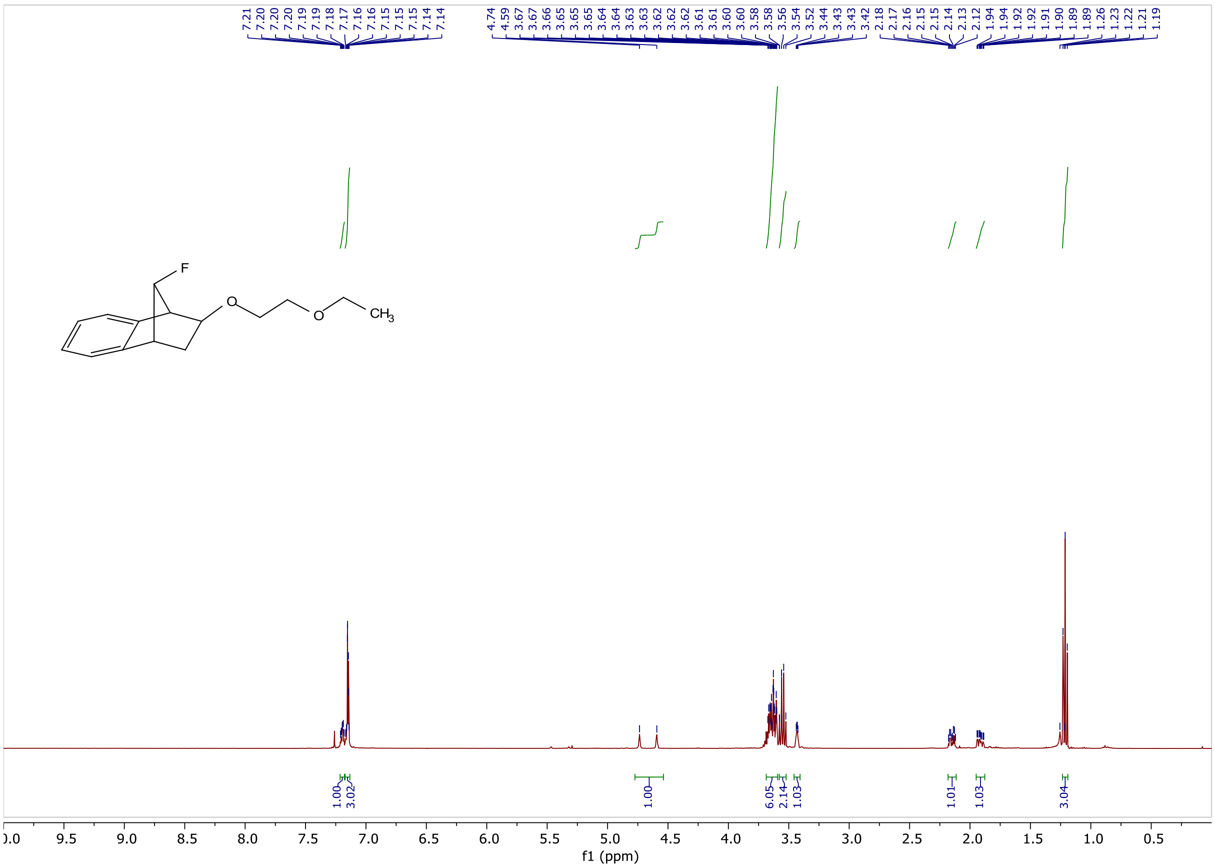

400 MHz ^{1}H-NMR spectrum of **3i** ($CDCl_3$)

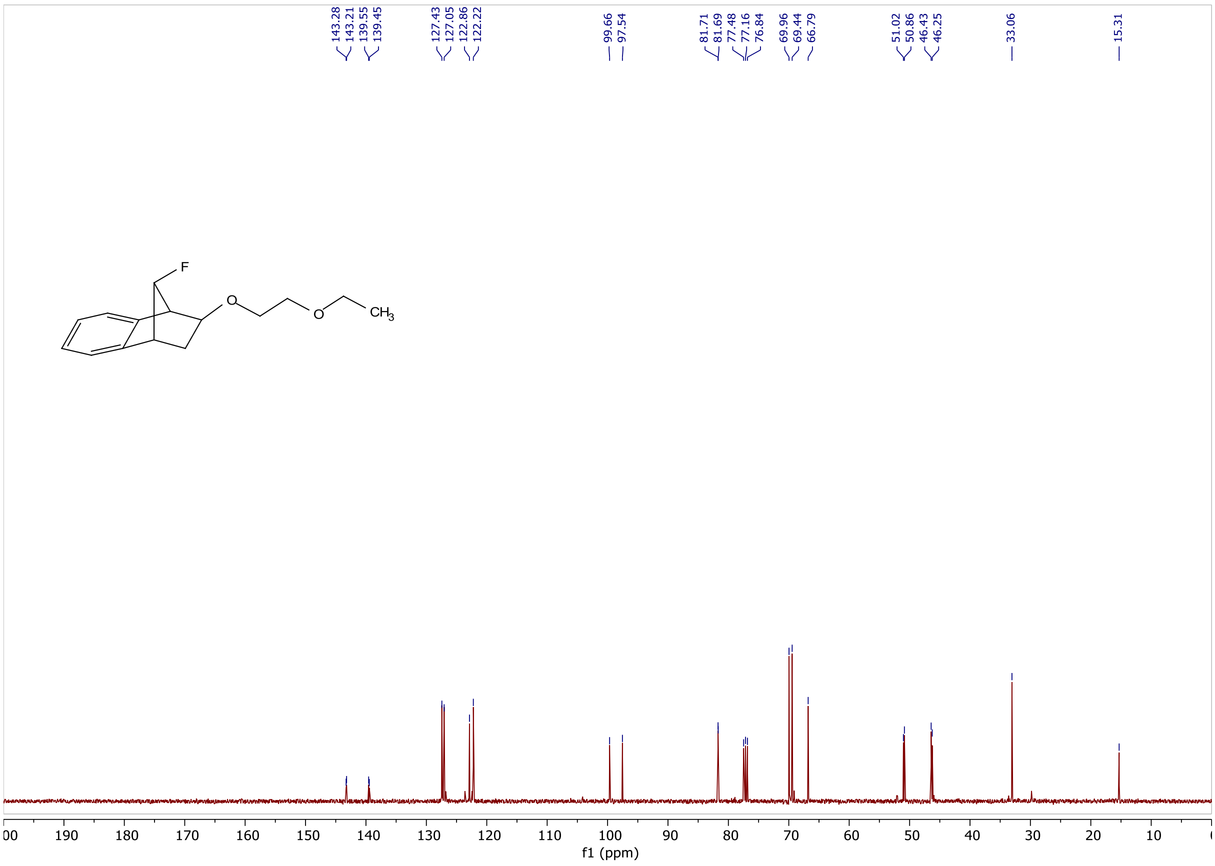

101 MHz ^{13}C-NMR spectrum of **3i** ($CDCl_3$)

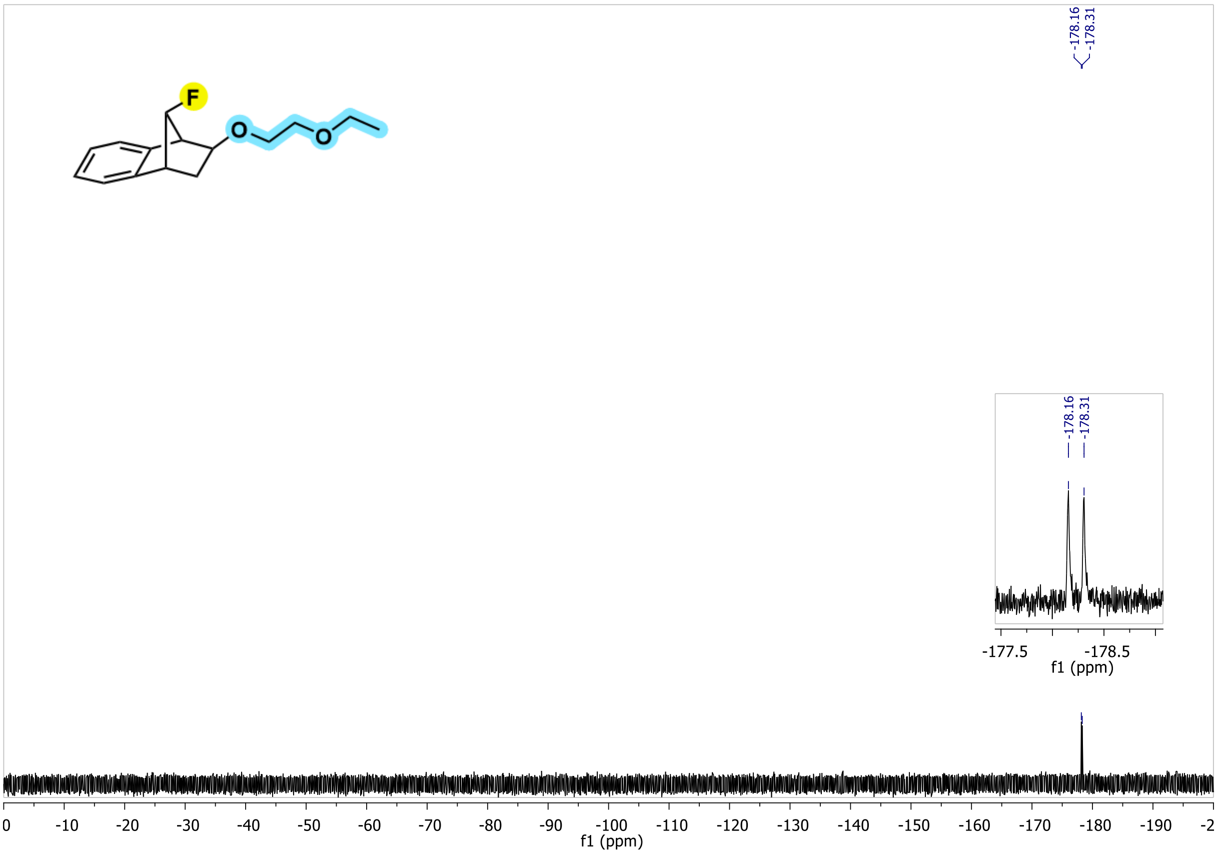

376 MHz ^{19}F-NMR spectrum of **3i** ($CDCl_3$)

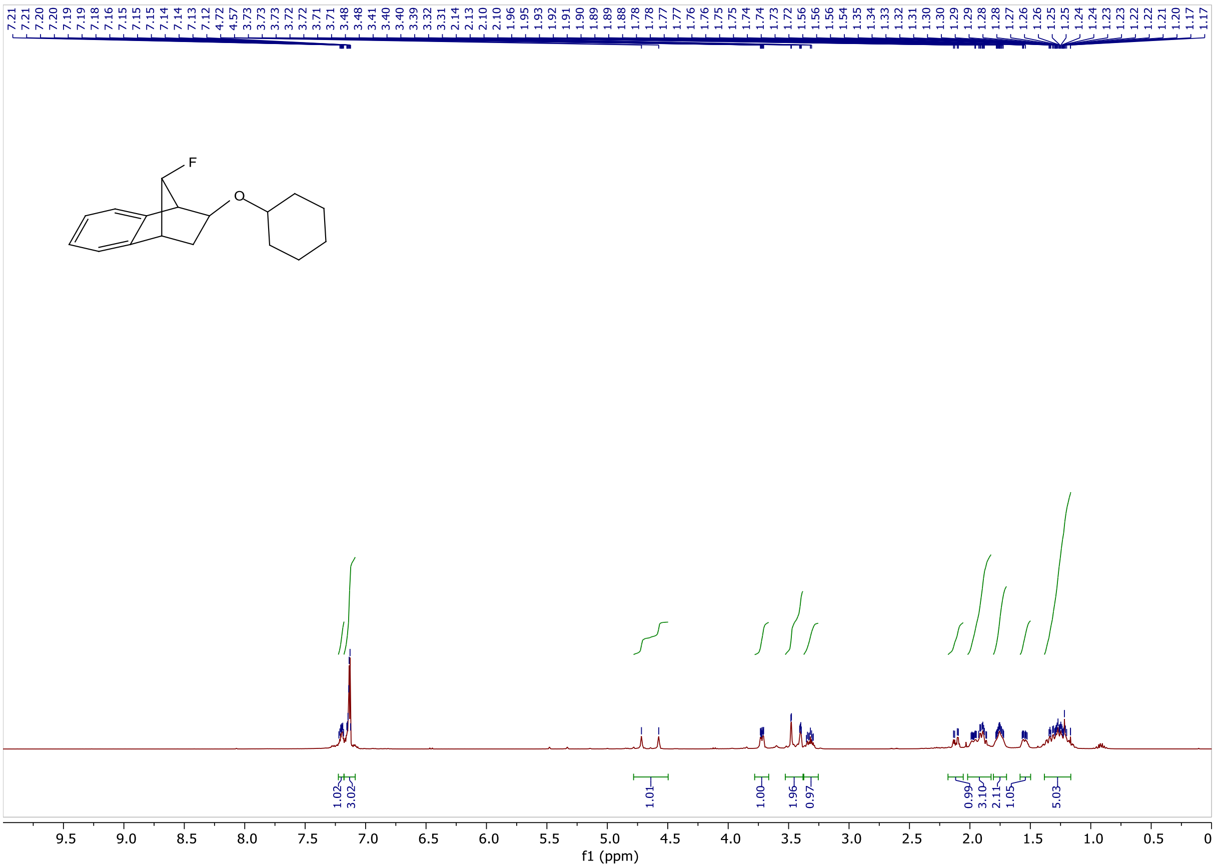

400 MHz ^{1}H-NMR spectrum of **3j** ($CDCl_3$)

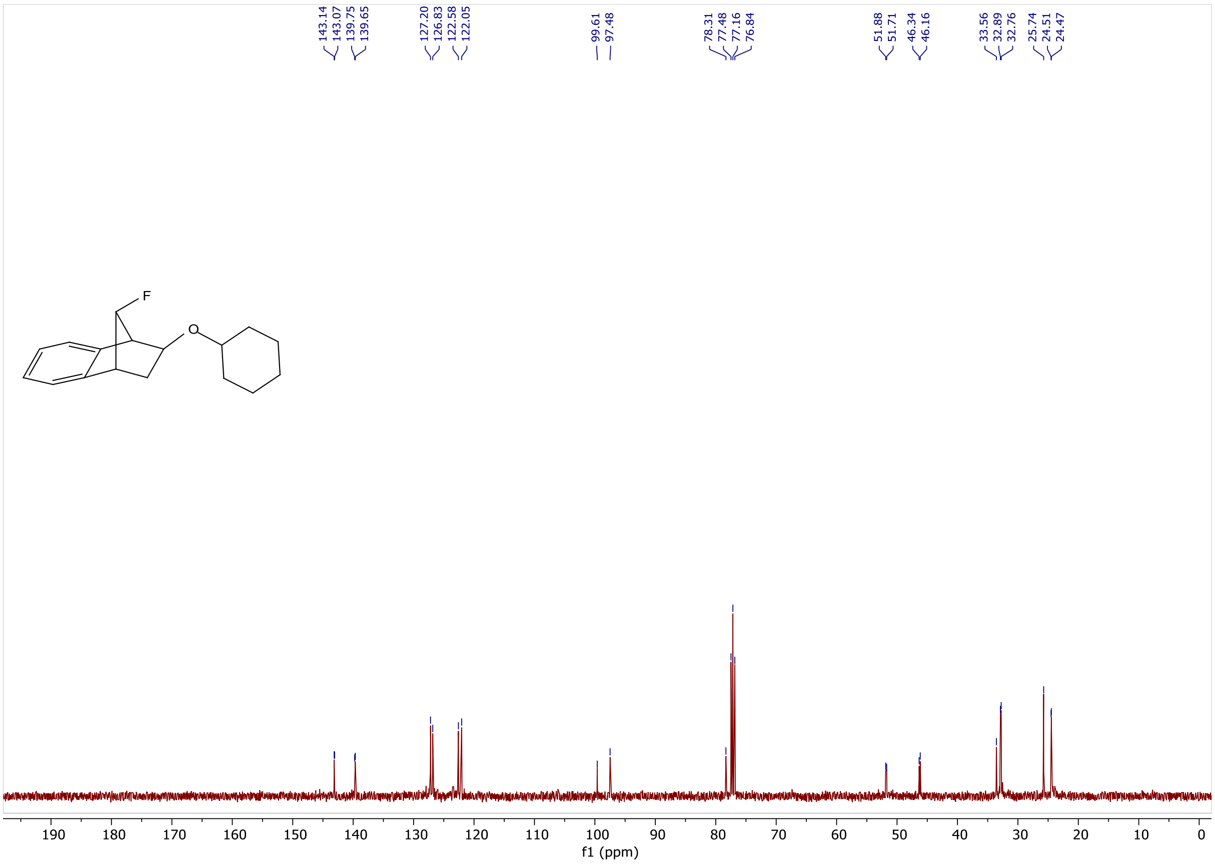

101 MHz ^{13}C-NMR spectrum of **3j** ($CDCl_3$)

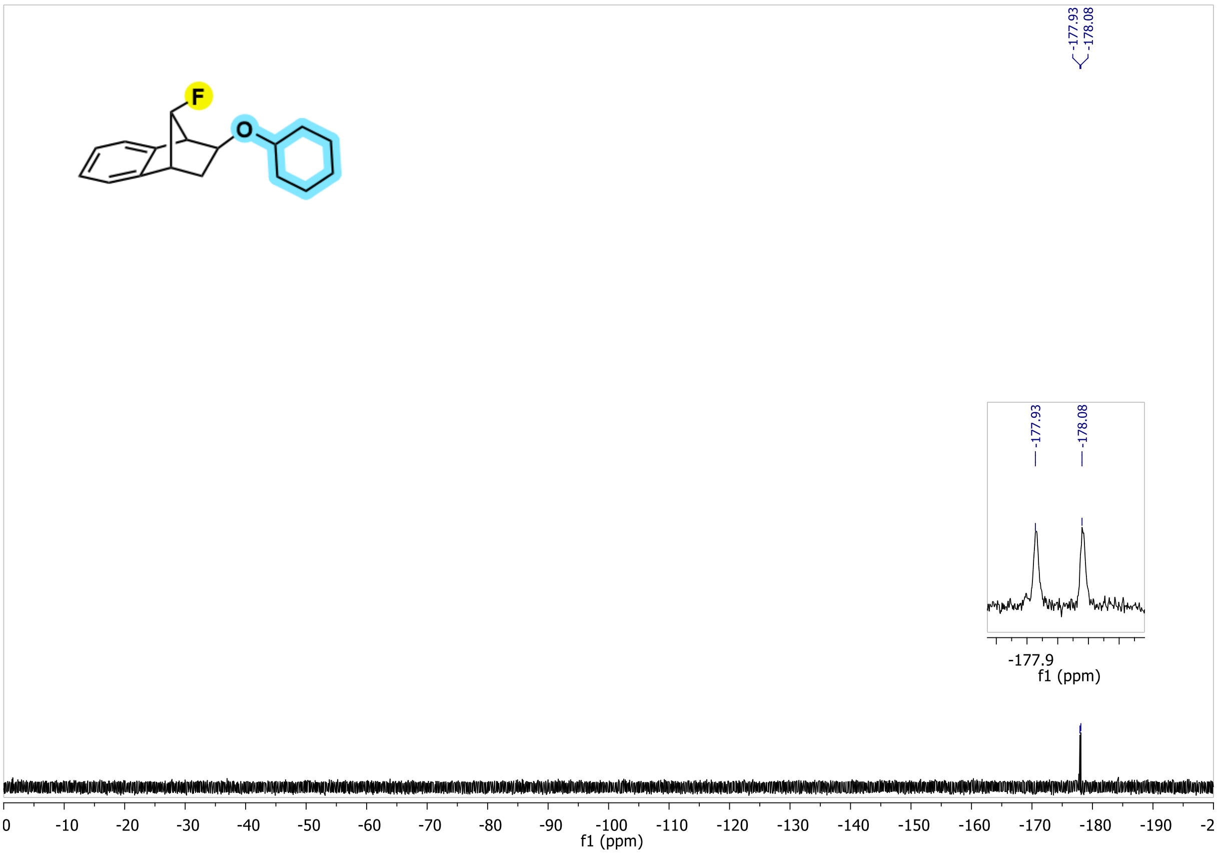

376 MHz ^{19}F-NMR spectrum of **3j** ($CDCl_3$)

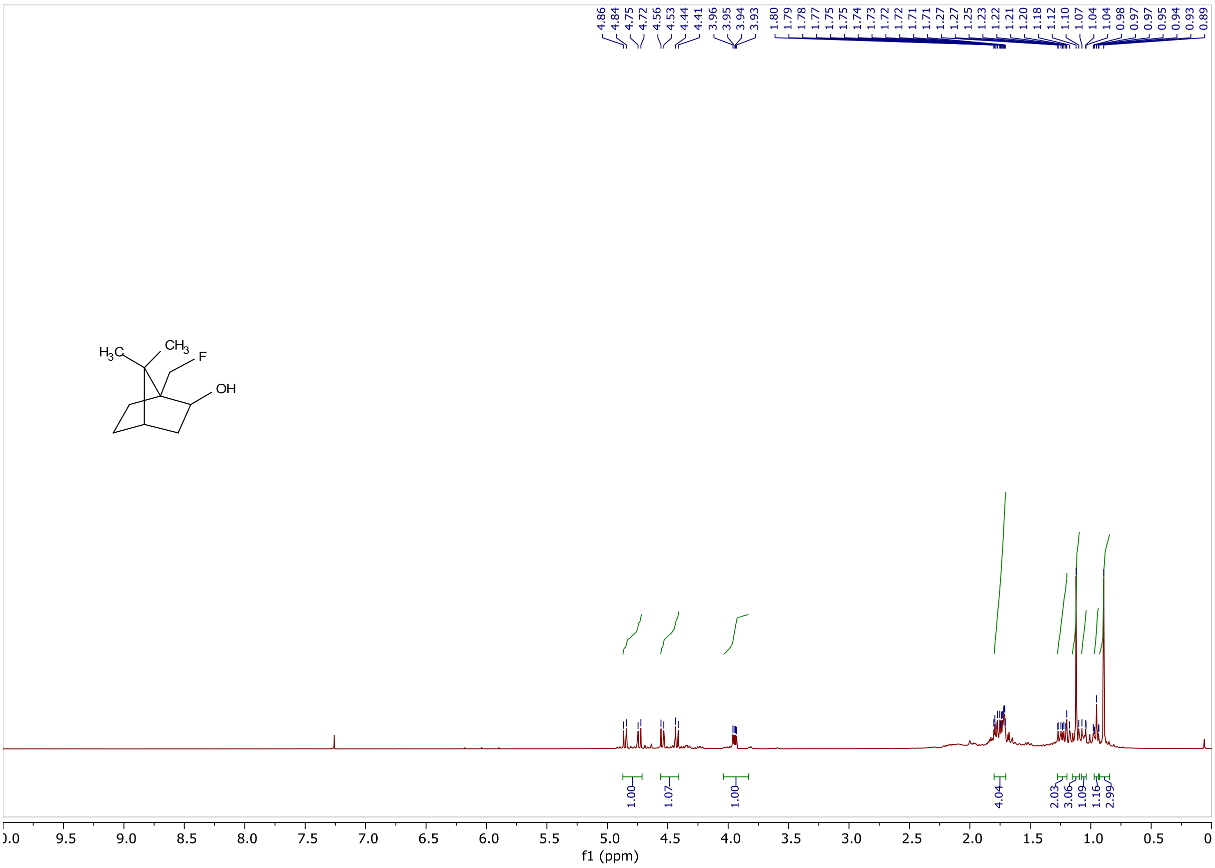

400 MHz ^{1}H-NMR spectrum of **4a** ($CDCl_3$)

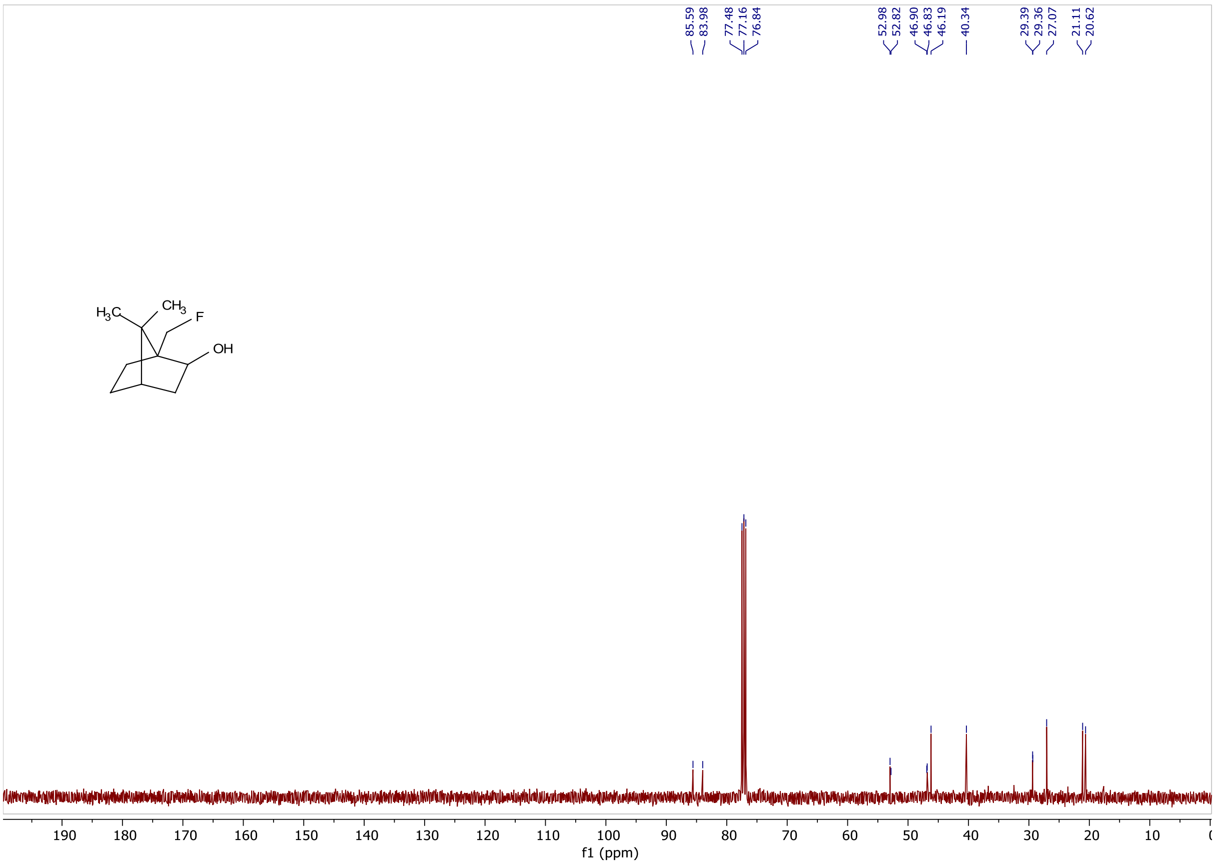

101 MHz ^{13}C-NMR spectrum of **4a** ($CDCl_3$)

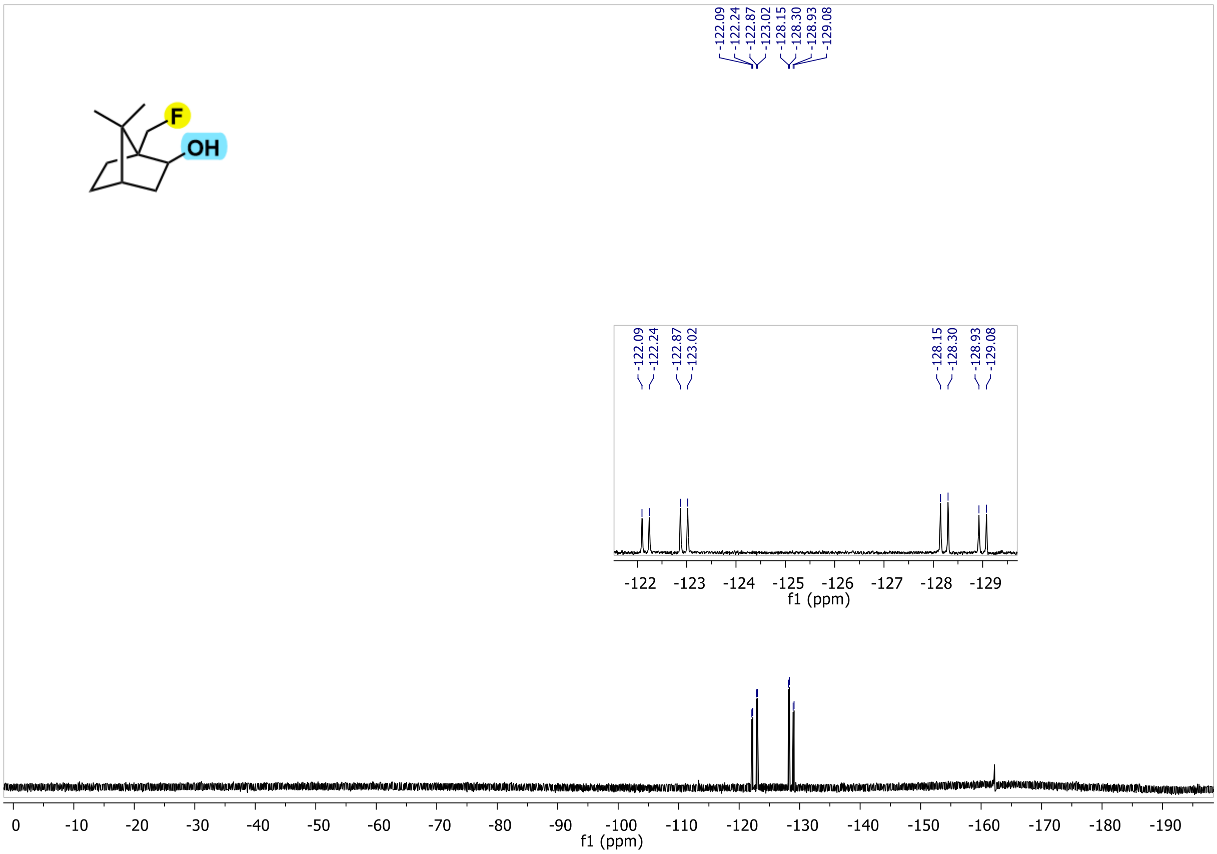

376 MHz ^{19}F-NMR spectrum of **4a** ($CDCl_3$)

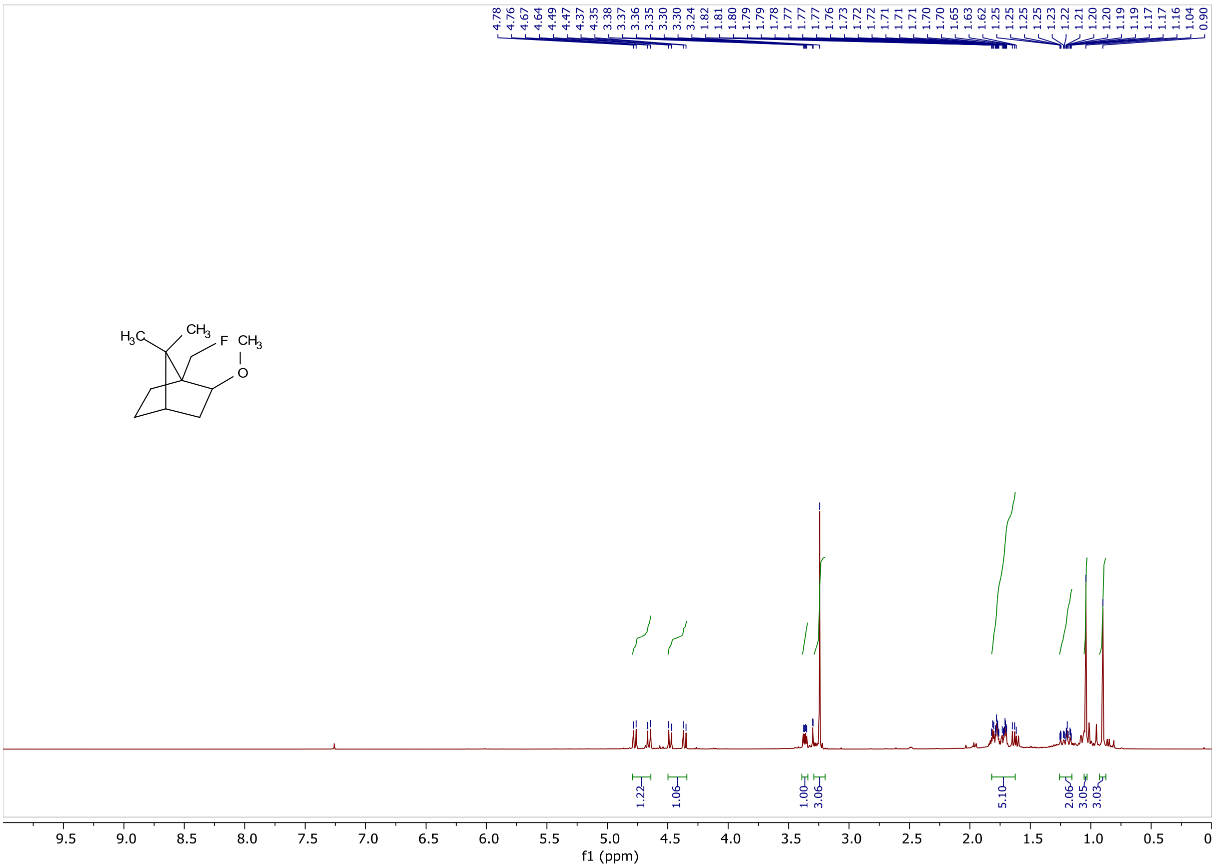

400 MHz ^{1}H-NMR spectrum of **4b** ($CDCl_3$)

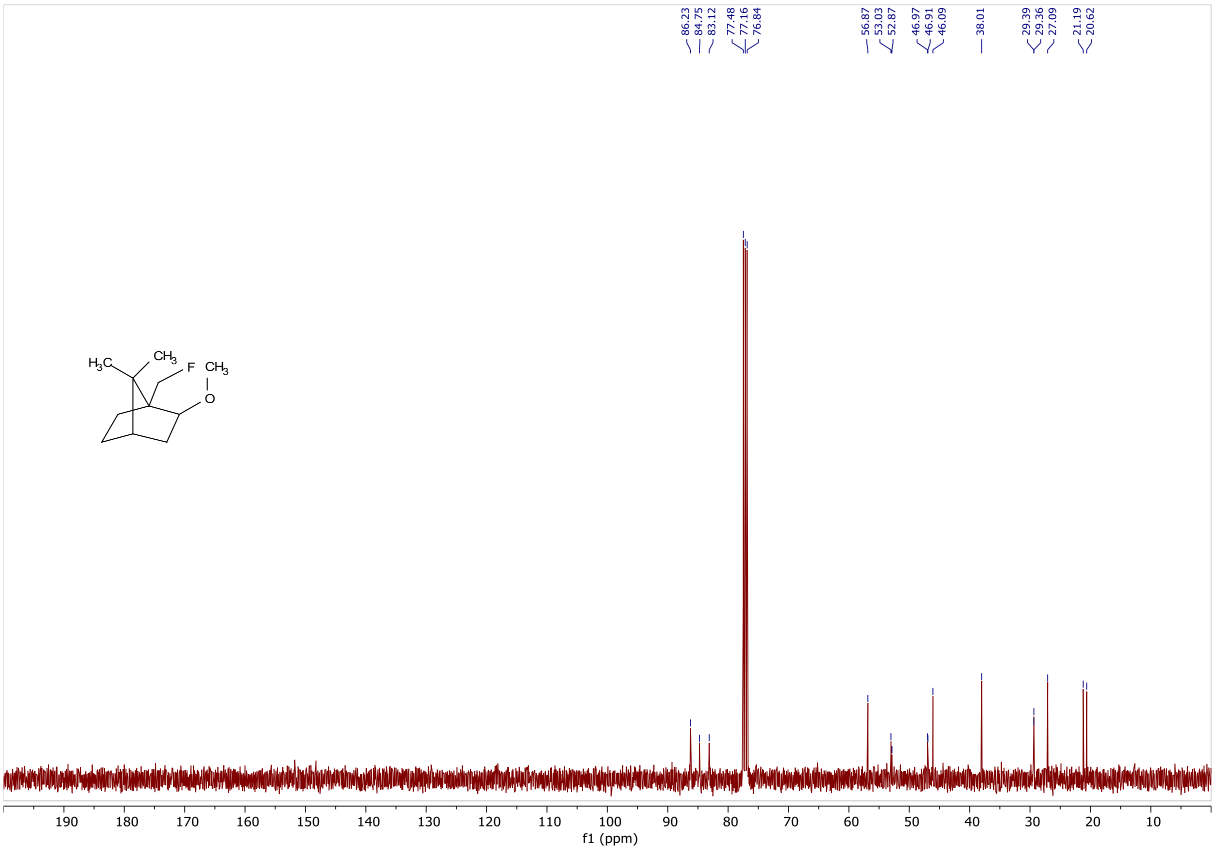

101 MHz ^{13}C-NMR spectrum of **4b** ($CDCl_3$)

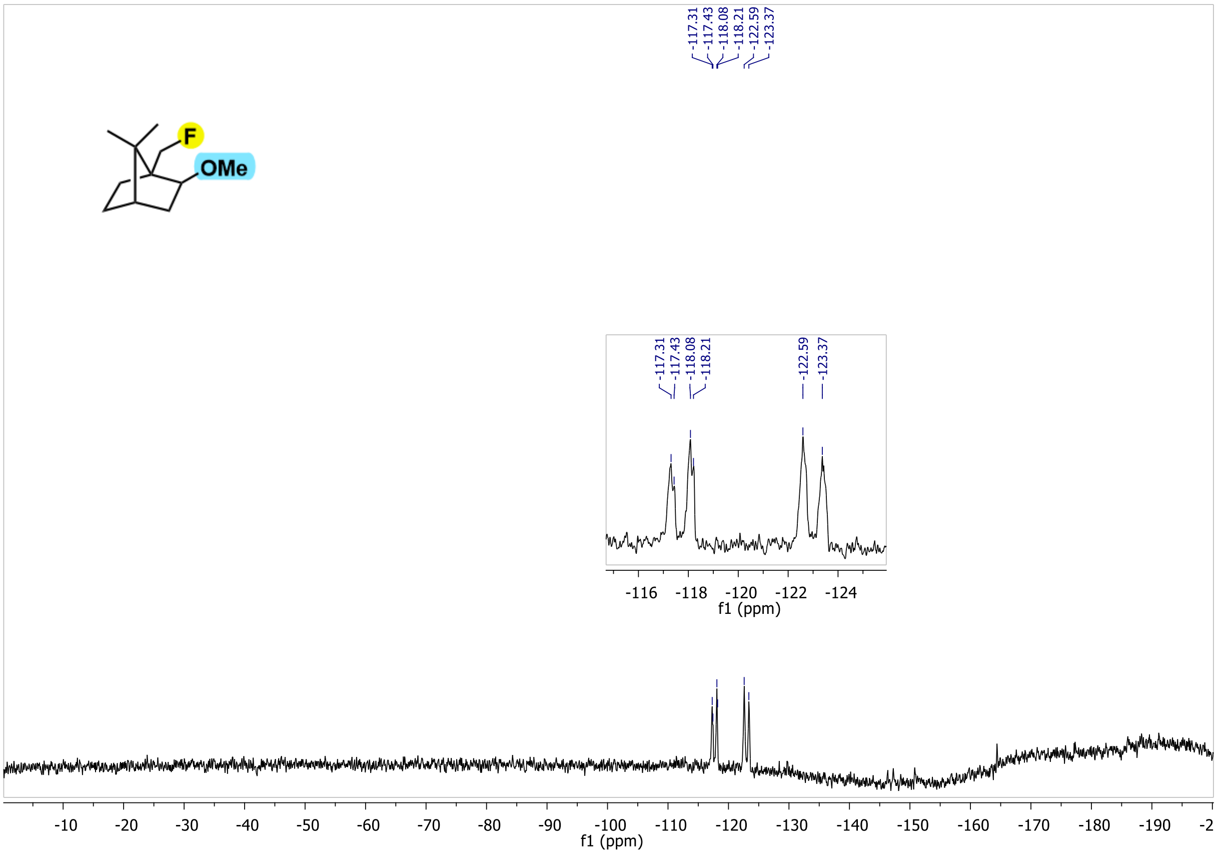

376 MHz ^{19}F-NMR spectrum of **4b** ($CDCl_3$)

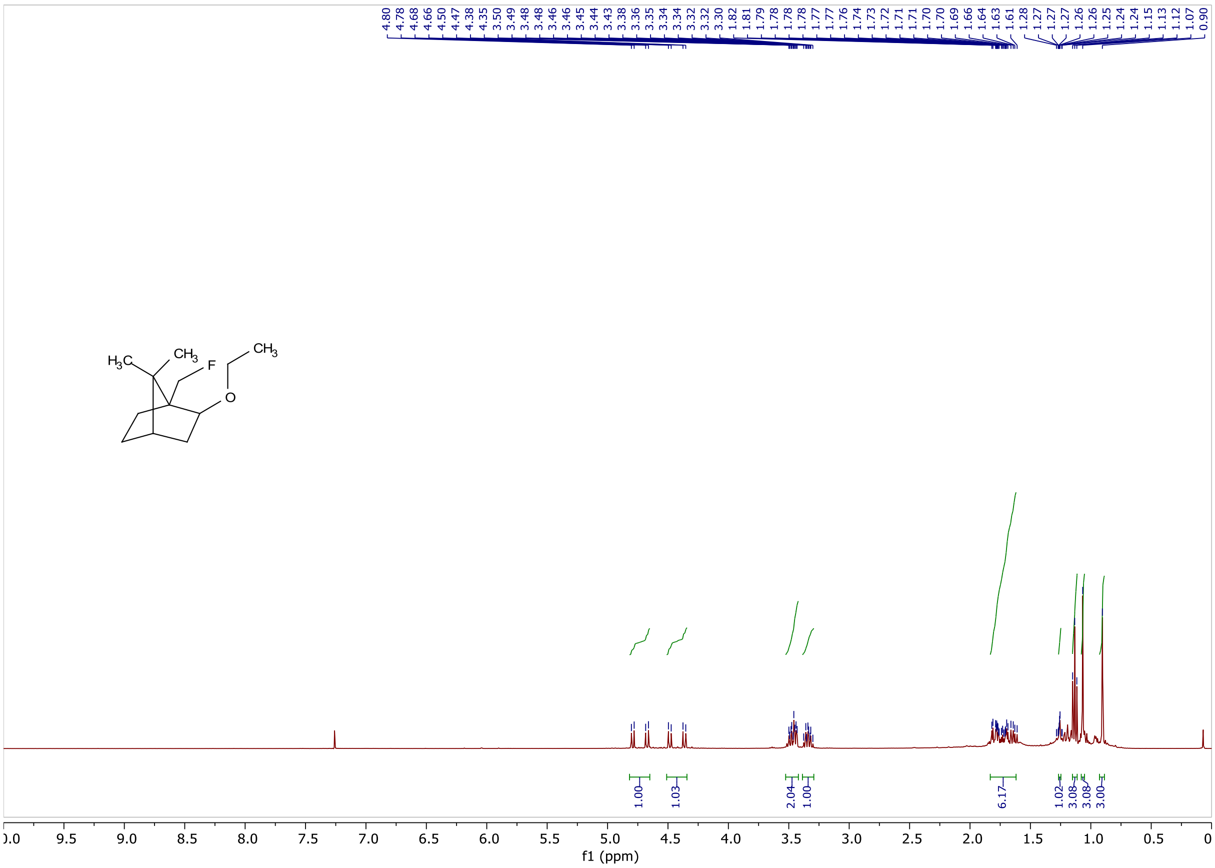

400 MHz ^{1}H-NMR) spectrum of **4c** ($CDCl_3$)

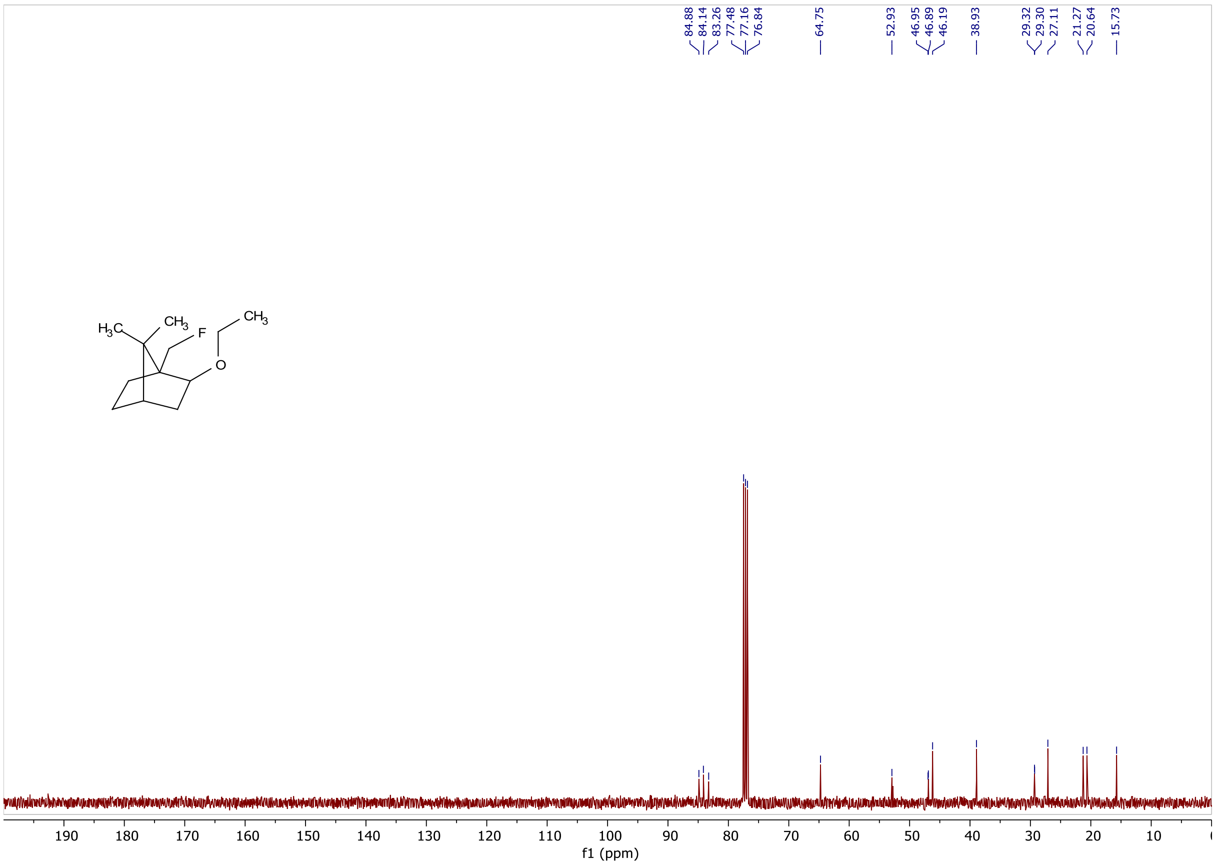

101 MHz ^{13}C-NMR spectrum of **4c** ($CDCl_3$)

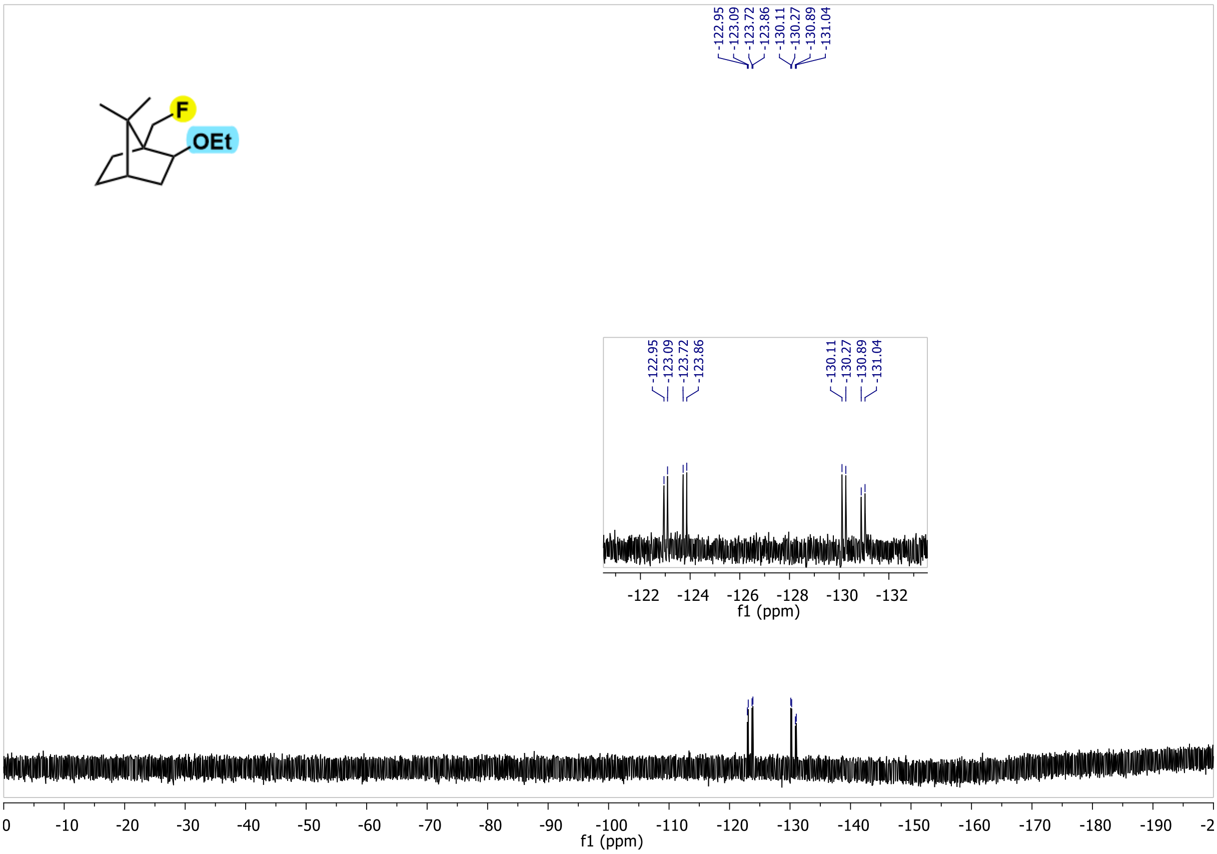

376 MHz ^{19}F-NMR spectrum of **4c** (CDCl$_3$)

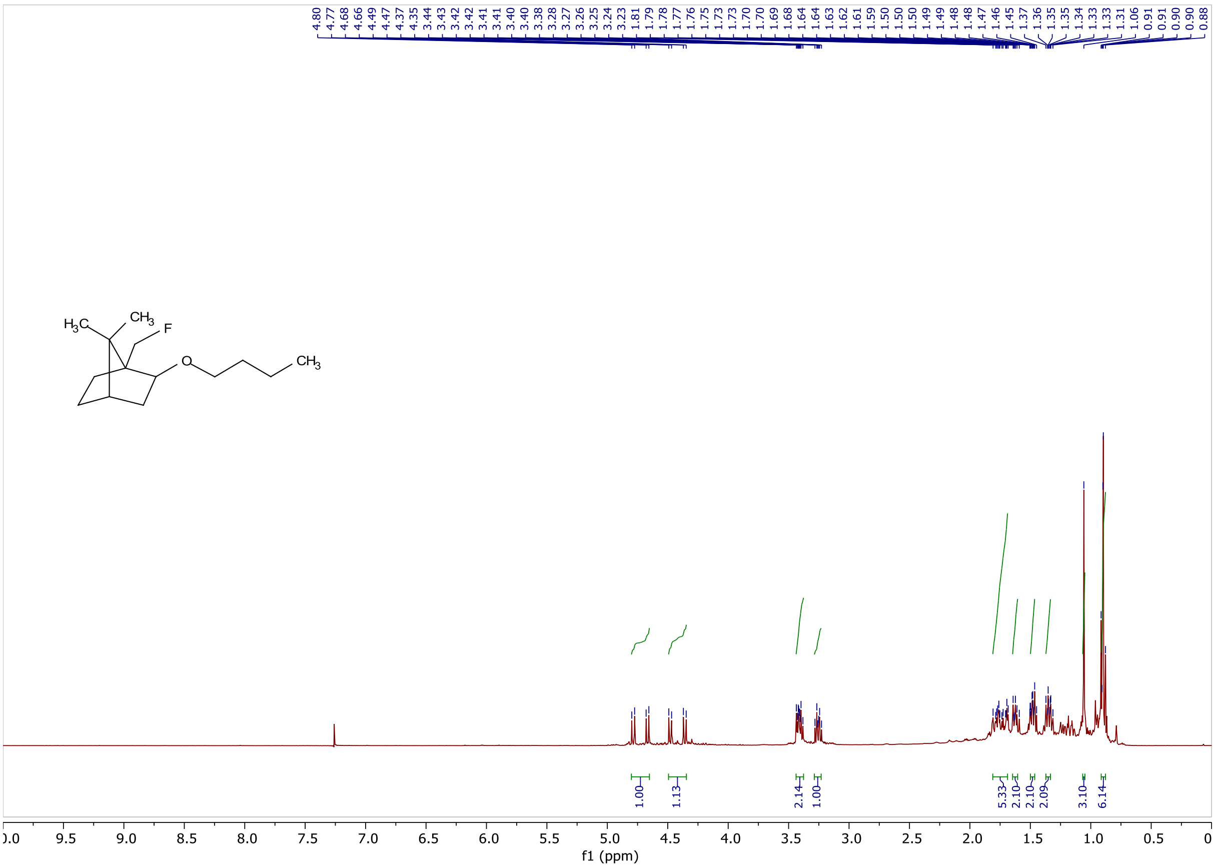

400 MHz ^{1}H-NMR spectrum of **4d** (CDCl$_3$)

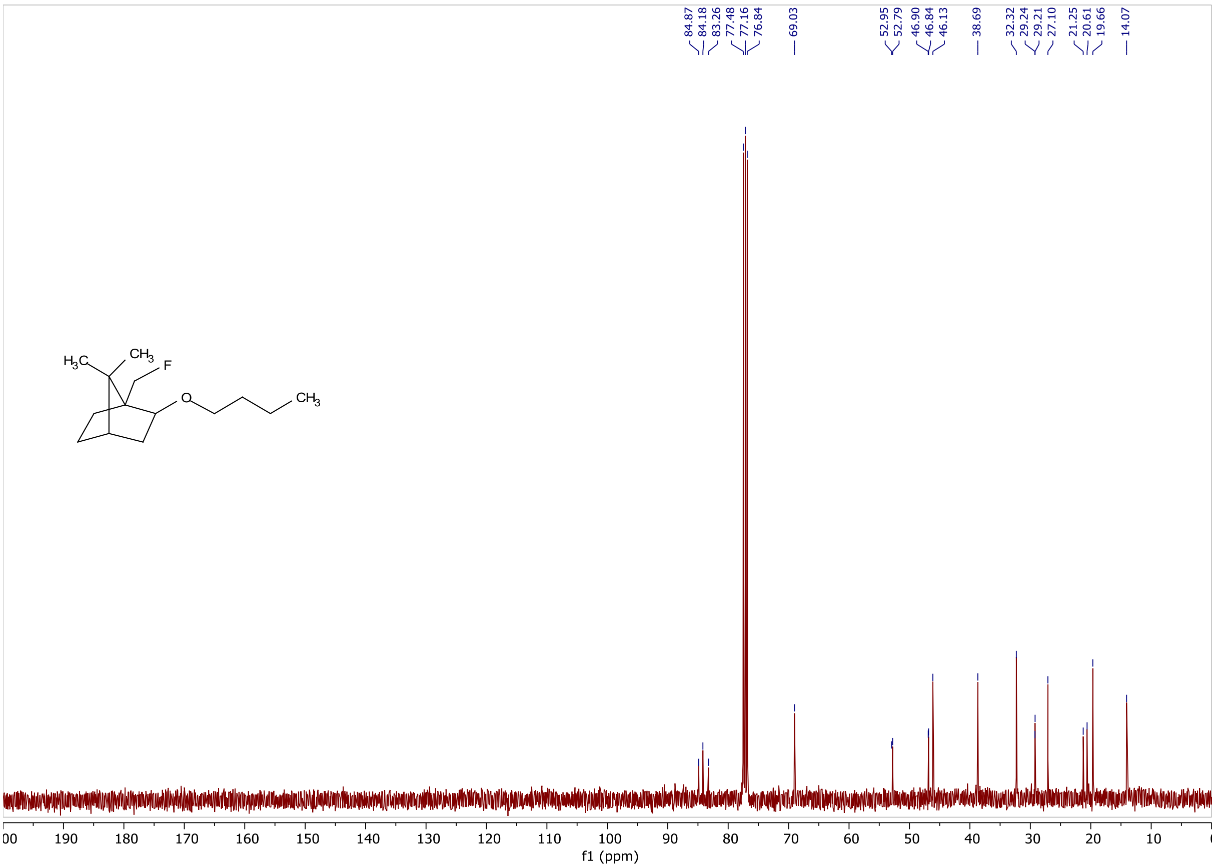

101 MHz ^{13}C-NMR spectrum of **4d** ($CDCl_3$)

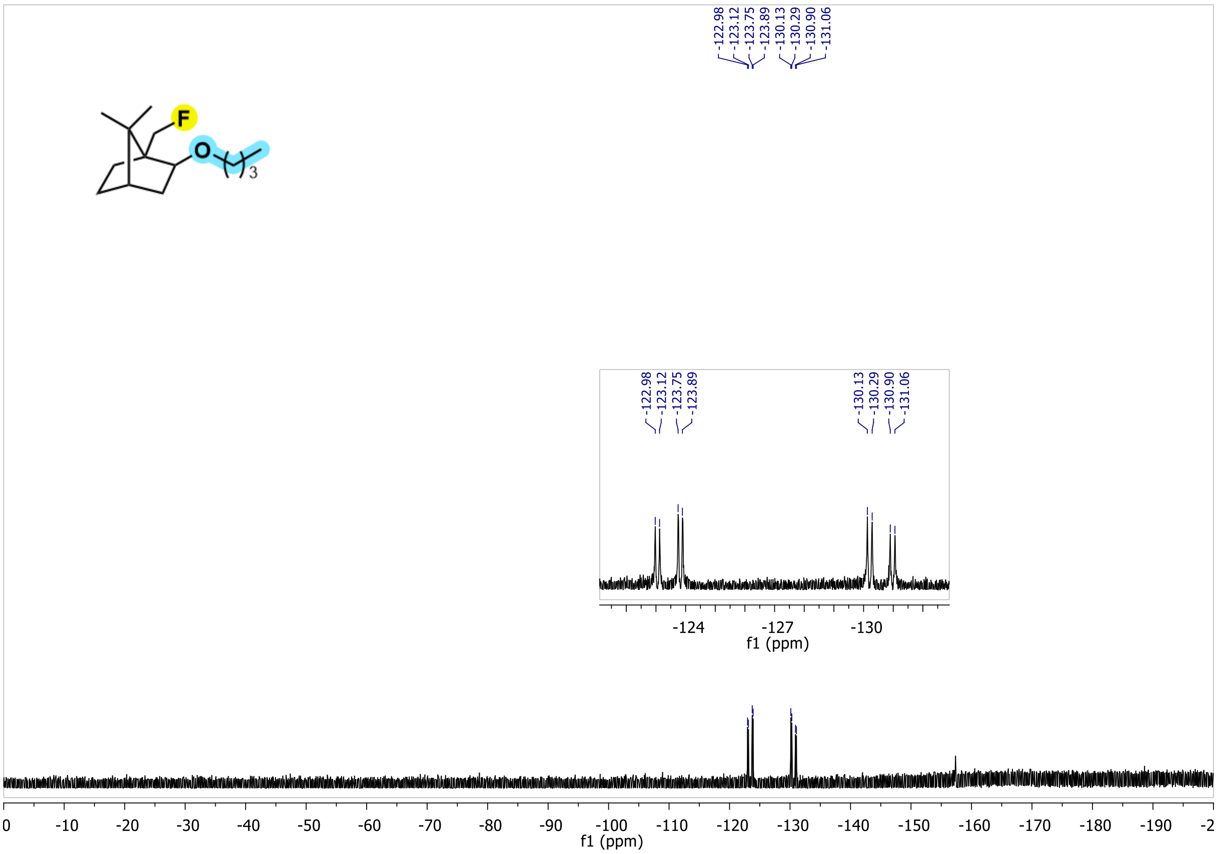

376 MHz ^{19}F-NMR spectrum of **4d** ($CDCl_3$)

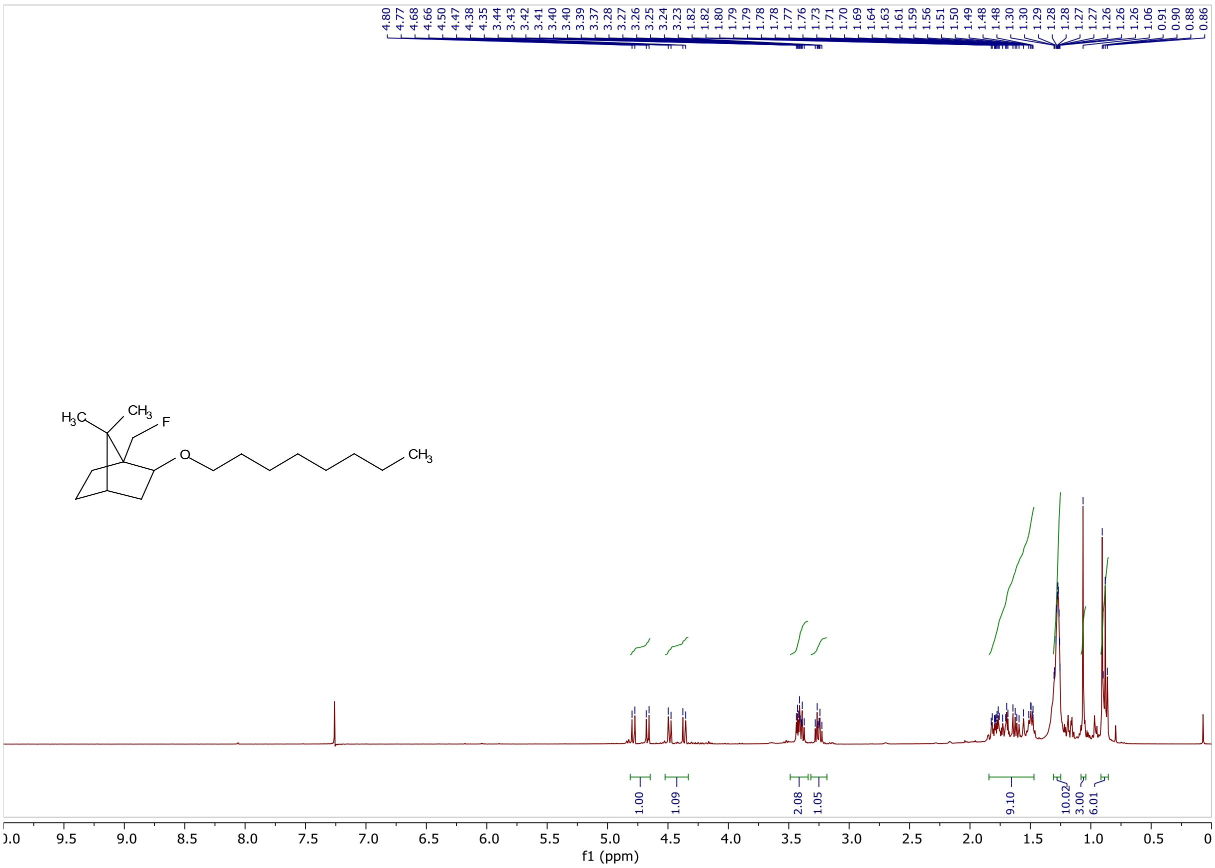

400 MHz ^{1}H-NMR spectrum of **4e** ($CDCl_3$)

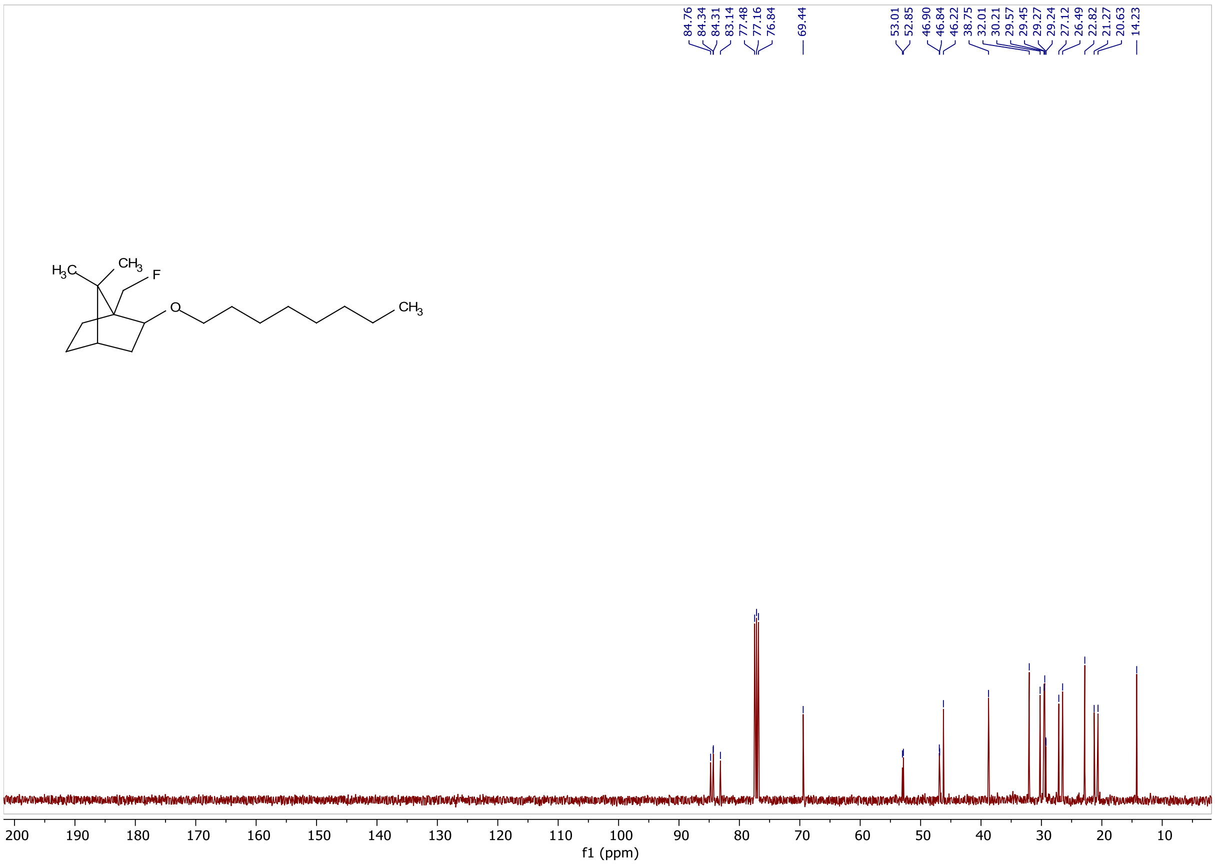

101 MHz ^{13}C-NMR spectrum of **4e** ($CDCl_3$)

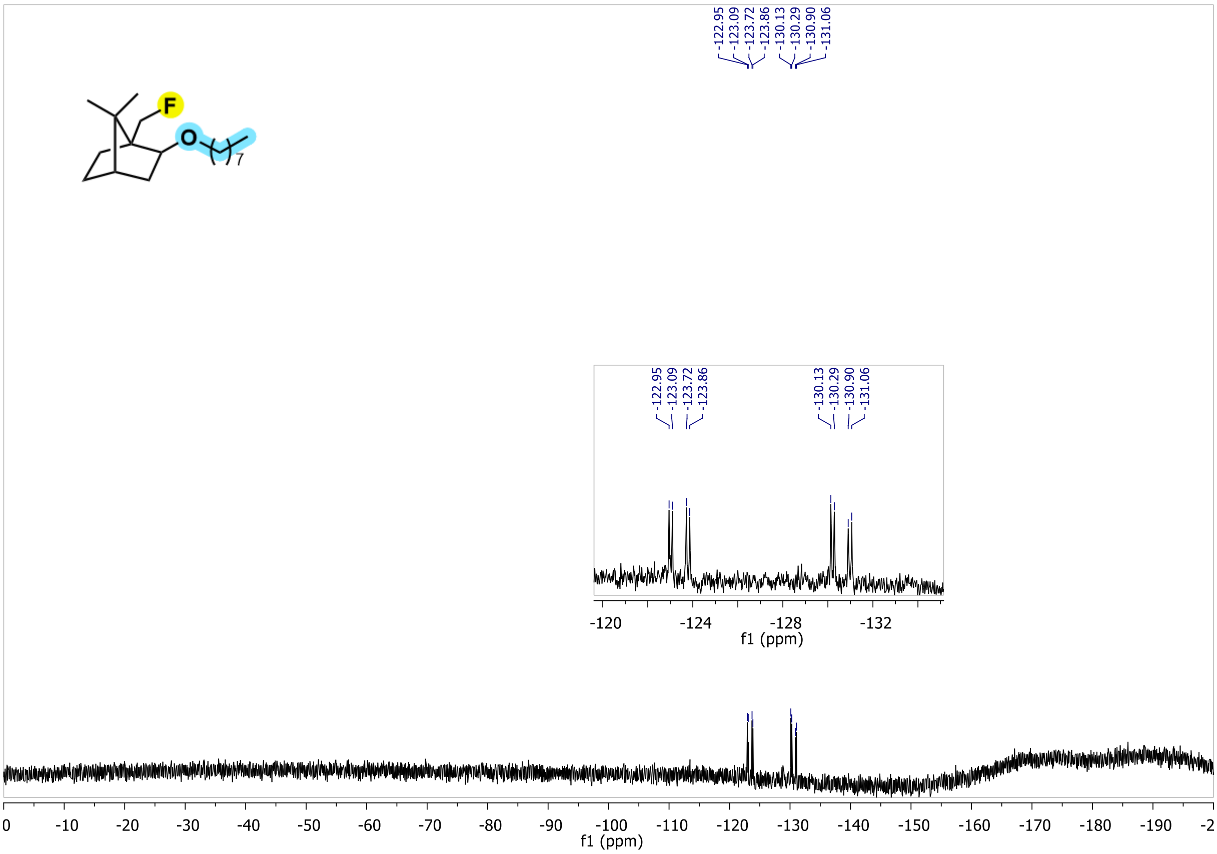

376 MHz ^{19}F-NMR spectrum of **4e** ($CDCl_3$)

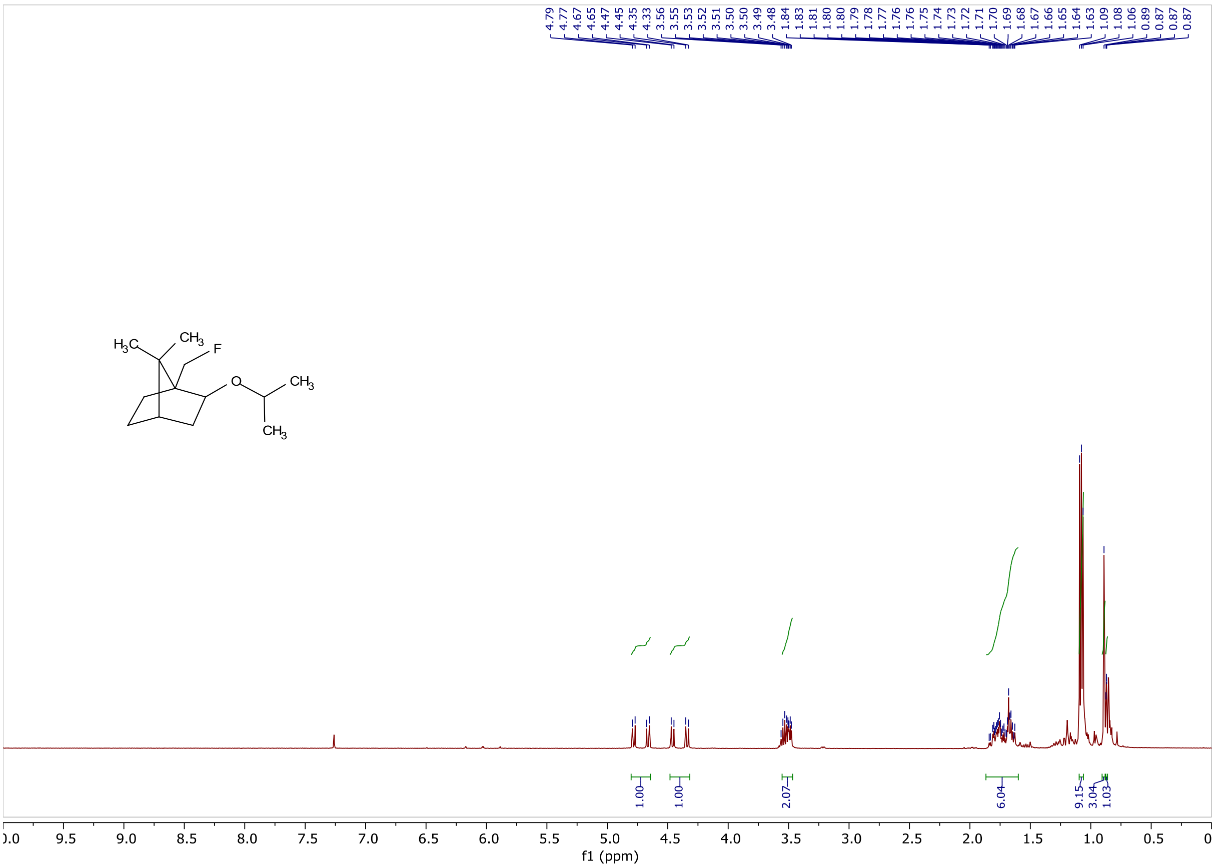

400 MHz ^{1}H-NMR spectrum of **4f** ($CDCl_3$)

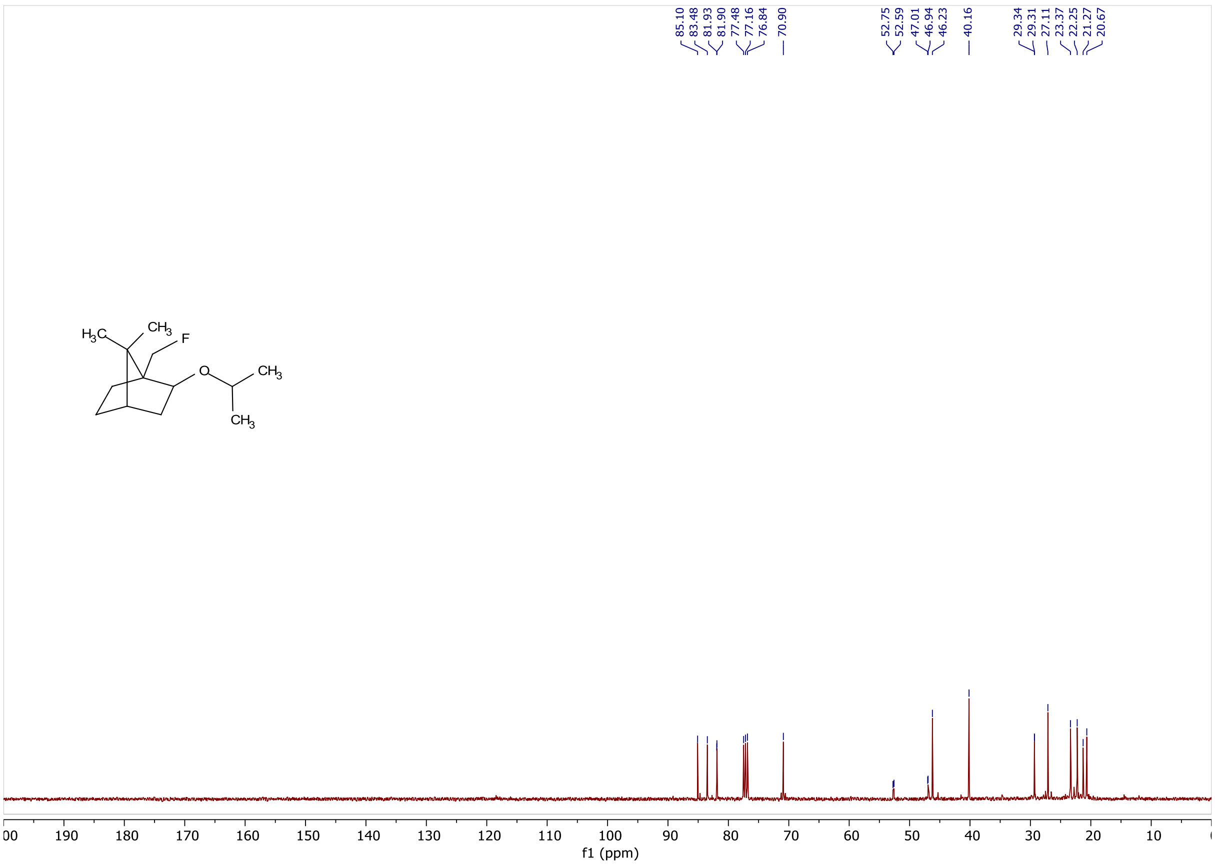

101 MHz ^{13}C-NMR spectrum of **4f** ($CDCl_3$)

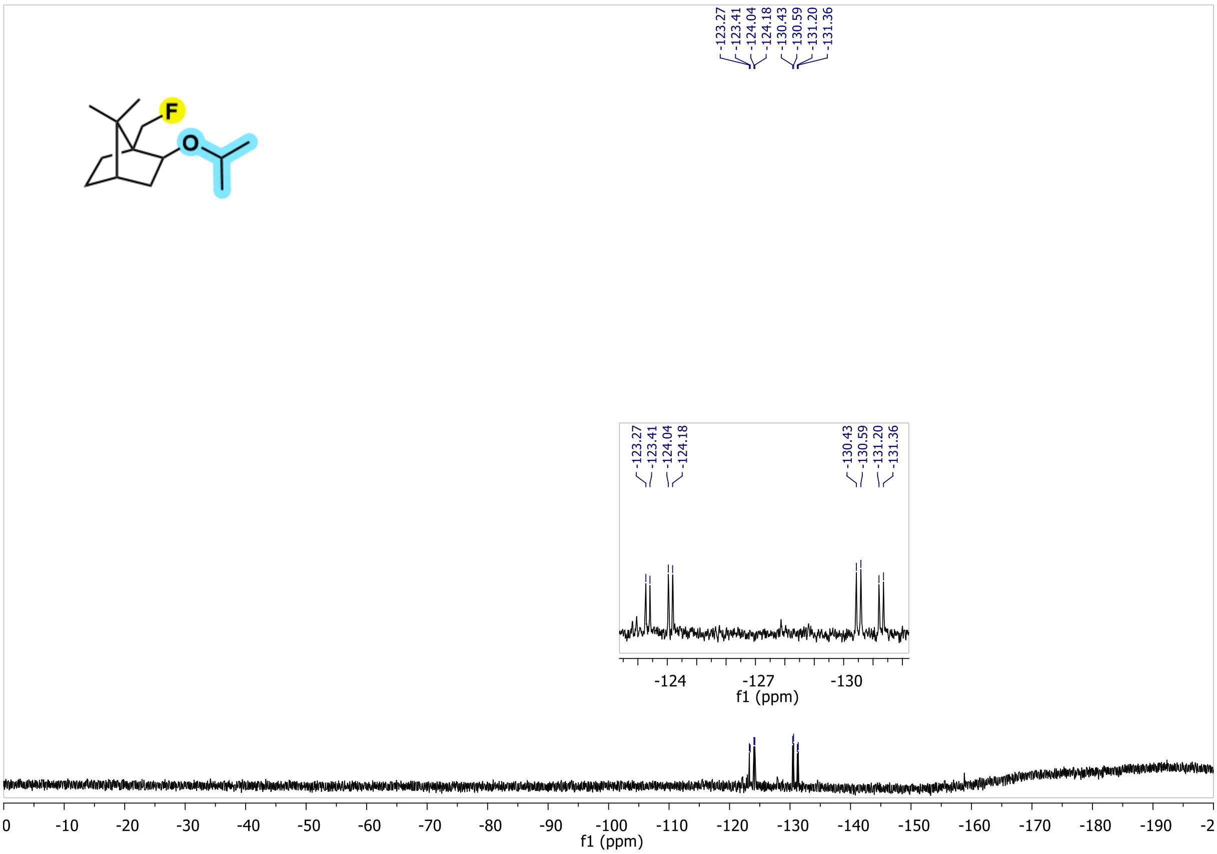

376 MHz ^{19}F-NMR spectrum of **4f** ($CDCl_3$)

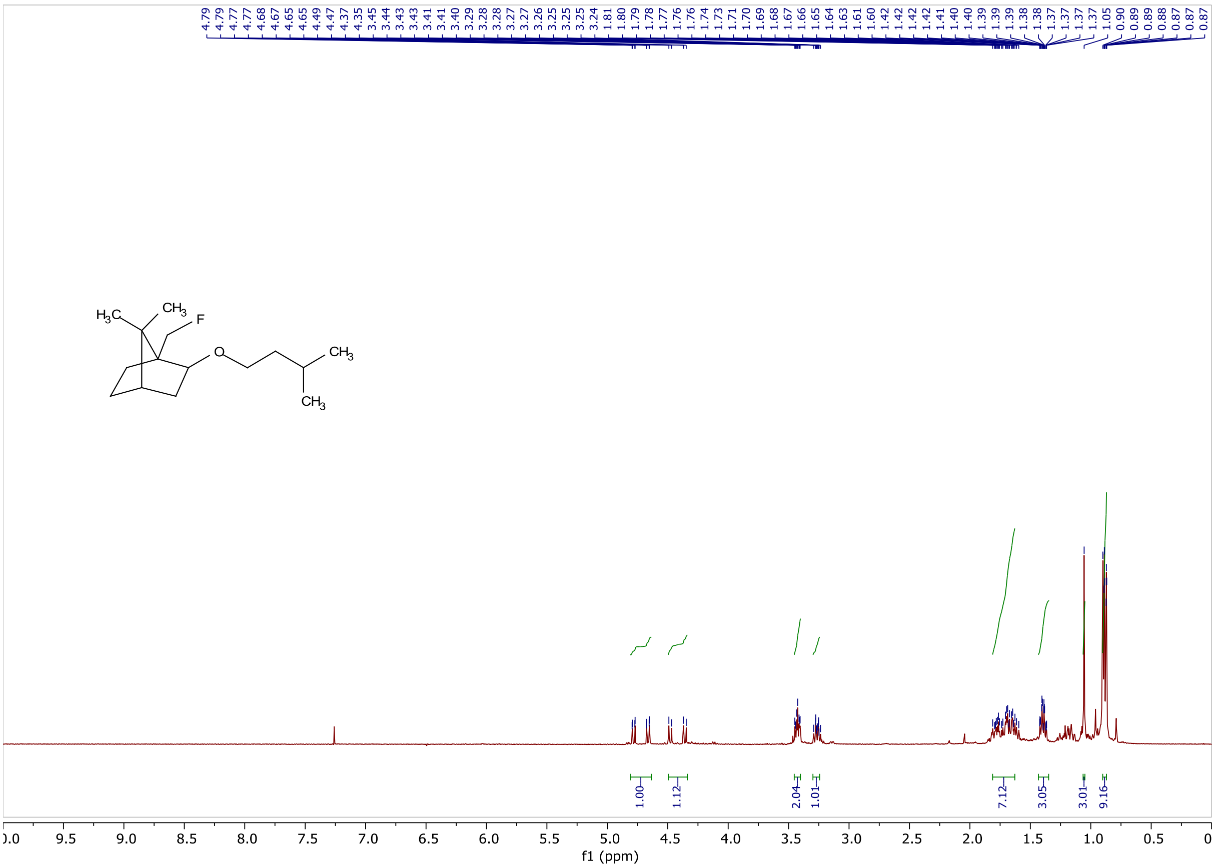

400 MHz ^{1}H-NMR spectrum of **4g** ($CDCl_3$)

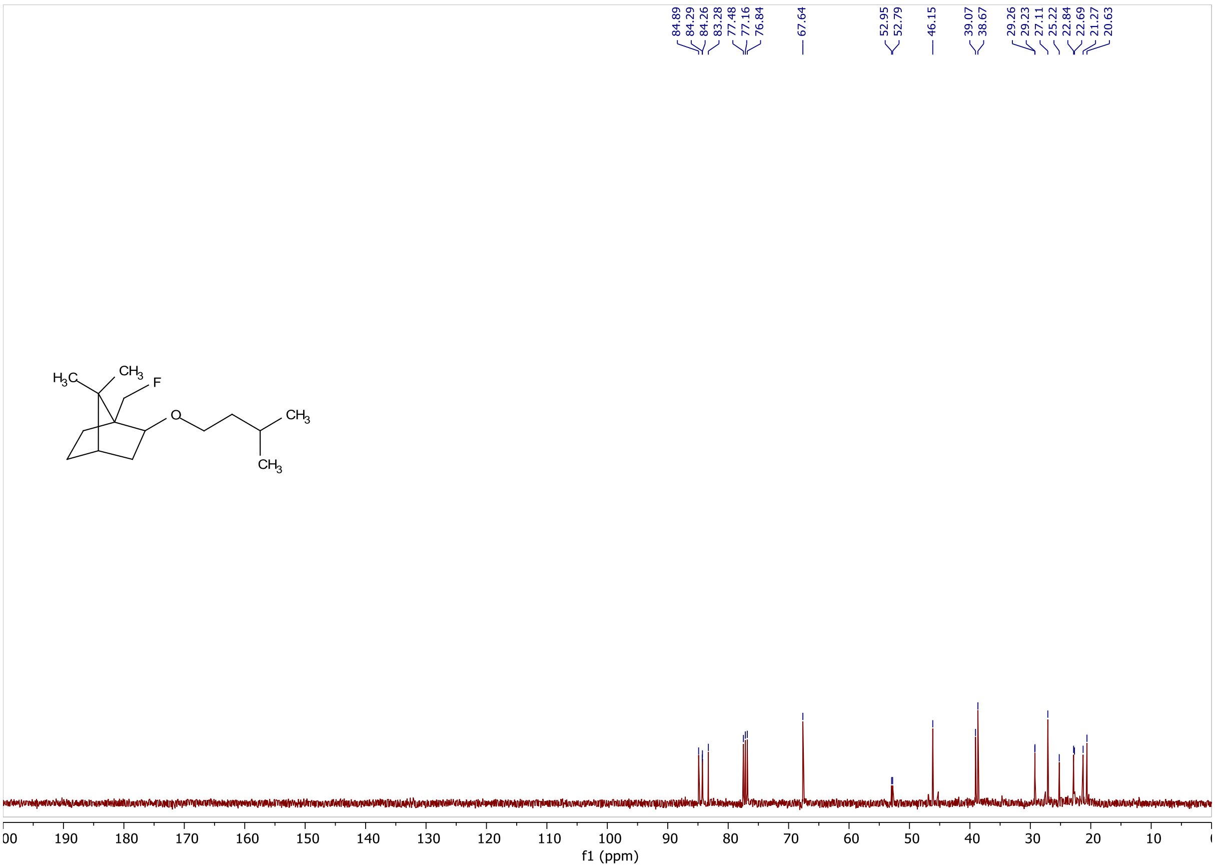

101 MHz ^{13}C-NMR spectrum of **4g** ($CDCl_3$)

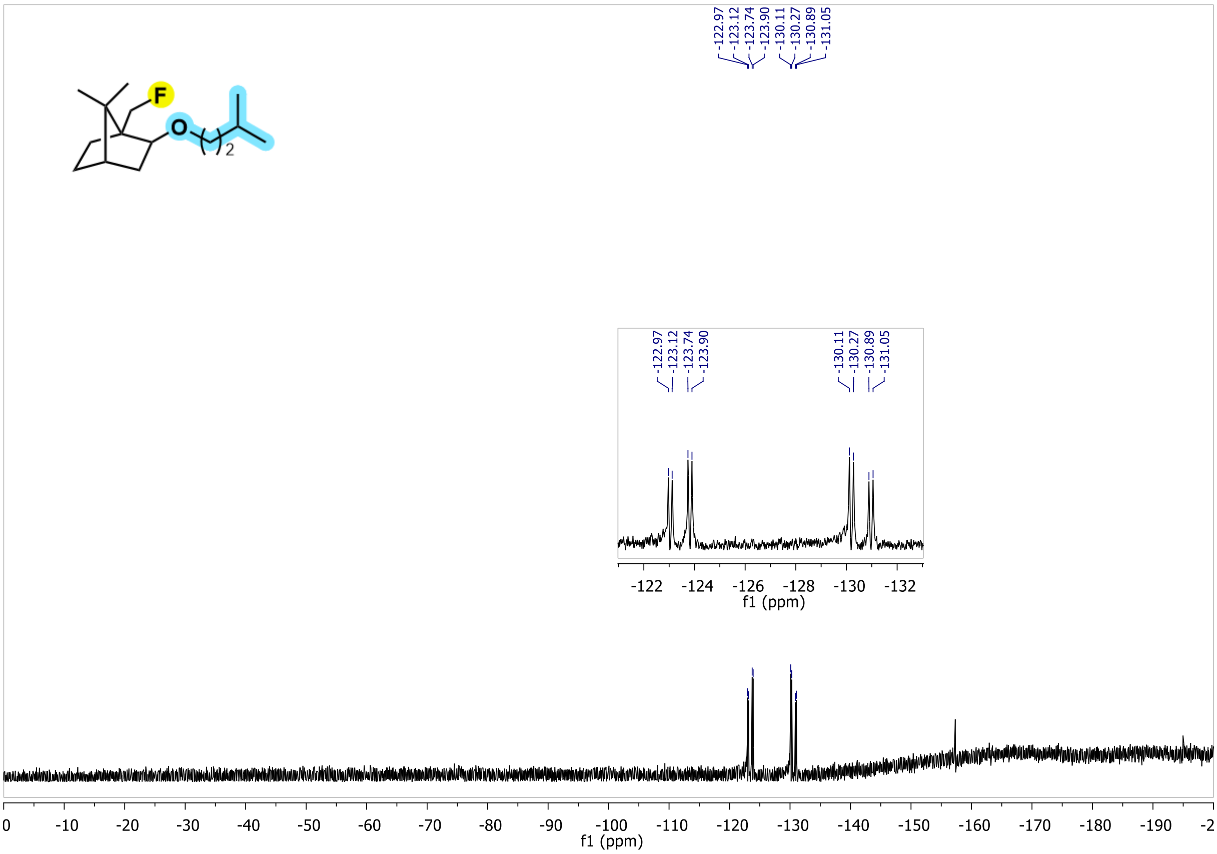

376 MHz ^{19}F-NMR spectrum of **4f** ($CDCl_3$)

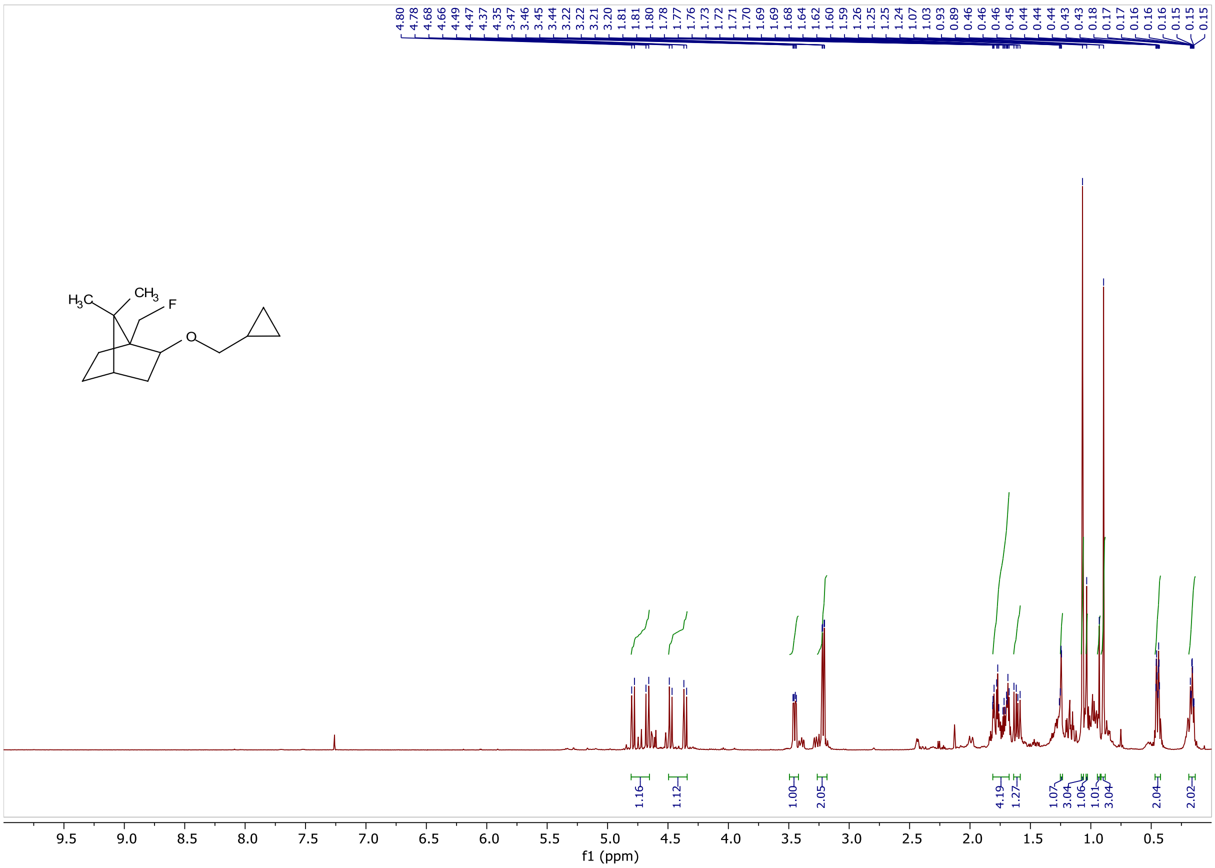

400 MHz ^{1}H-NMR spectrum of **4h** ($CDCl_3$)

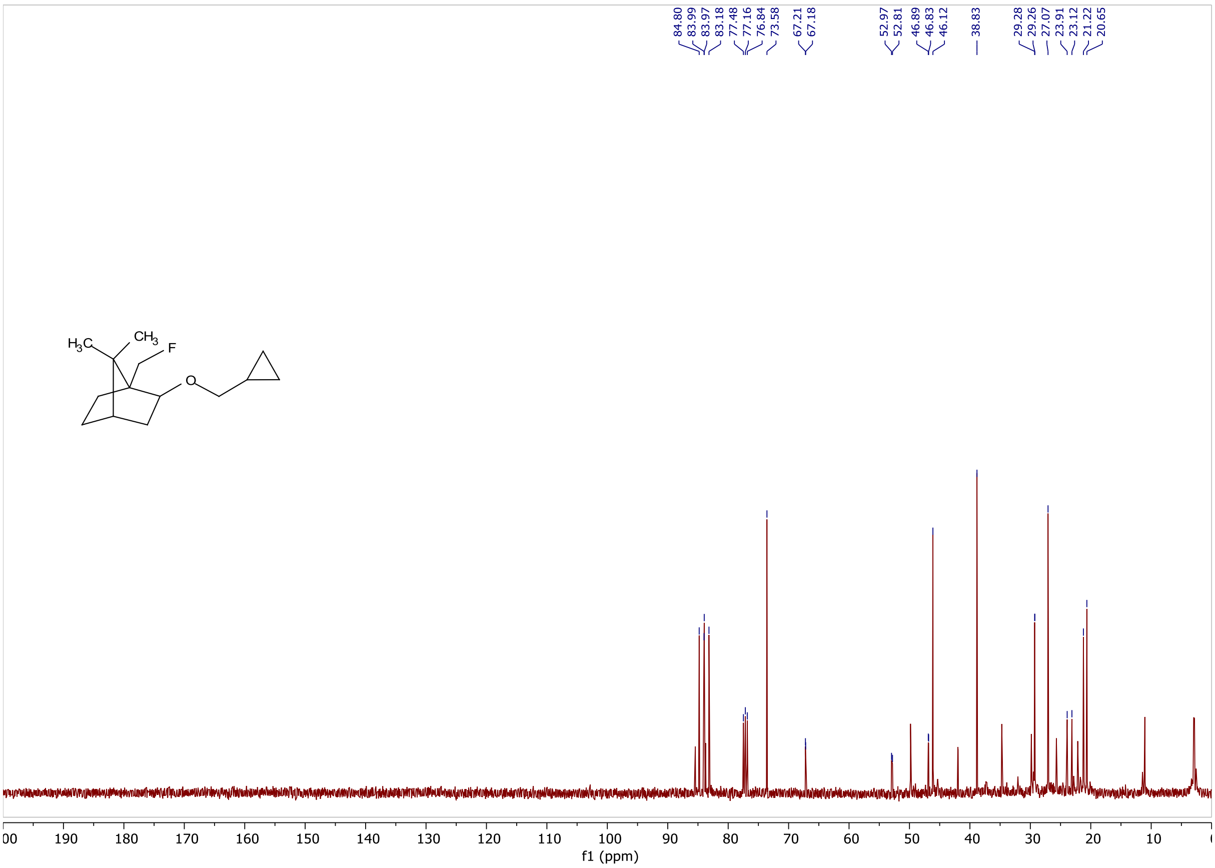

101 MHz ^{13}C-NMR spectrum of **4h** ($CDCl_3$)

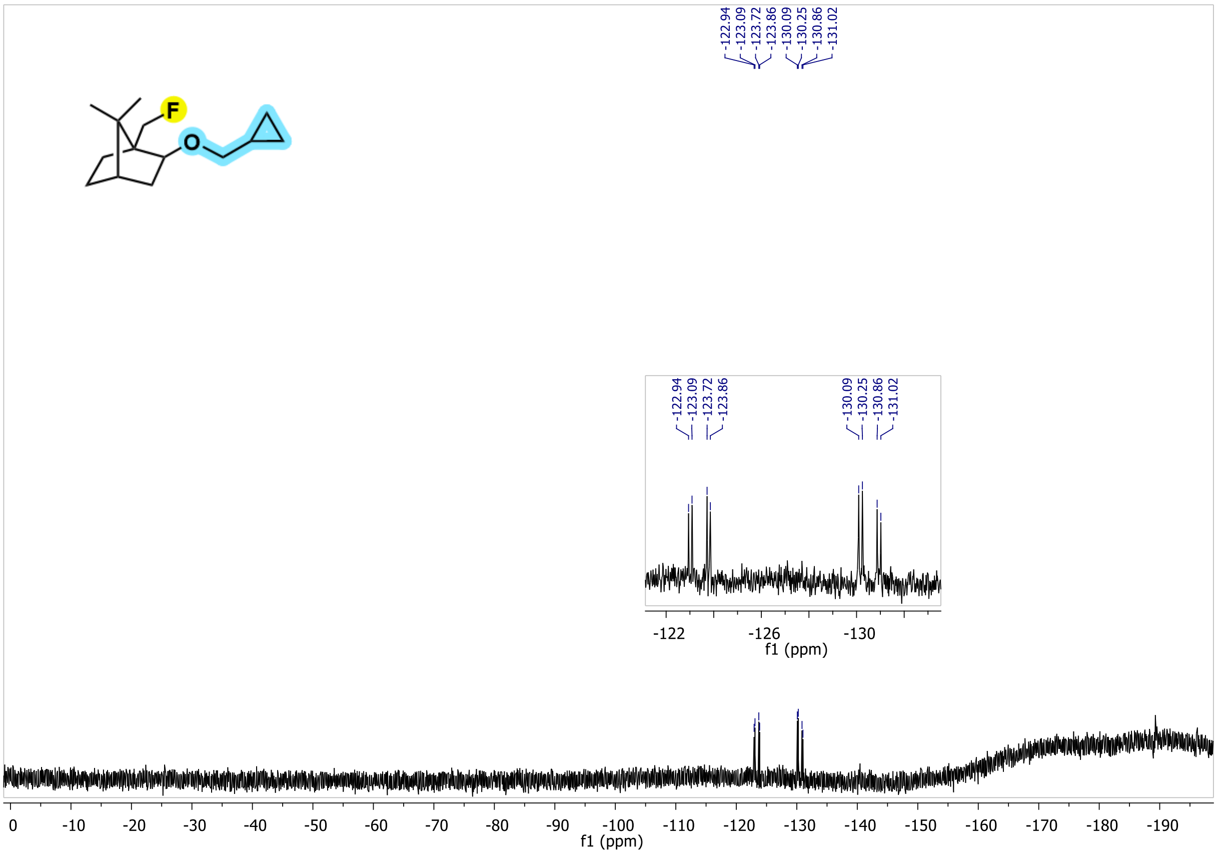

376 MHz ^{19}F-NMR spectrum of **4h** ($CDCl_3$)

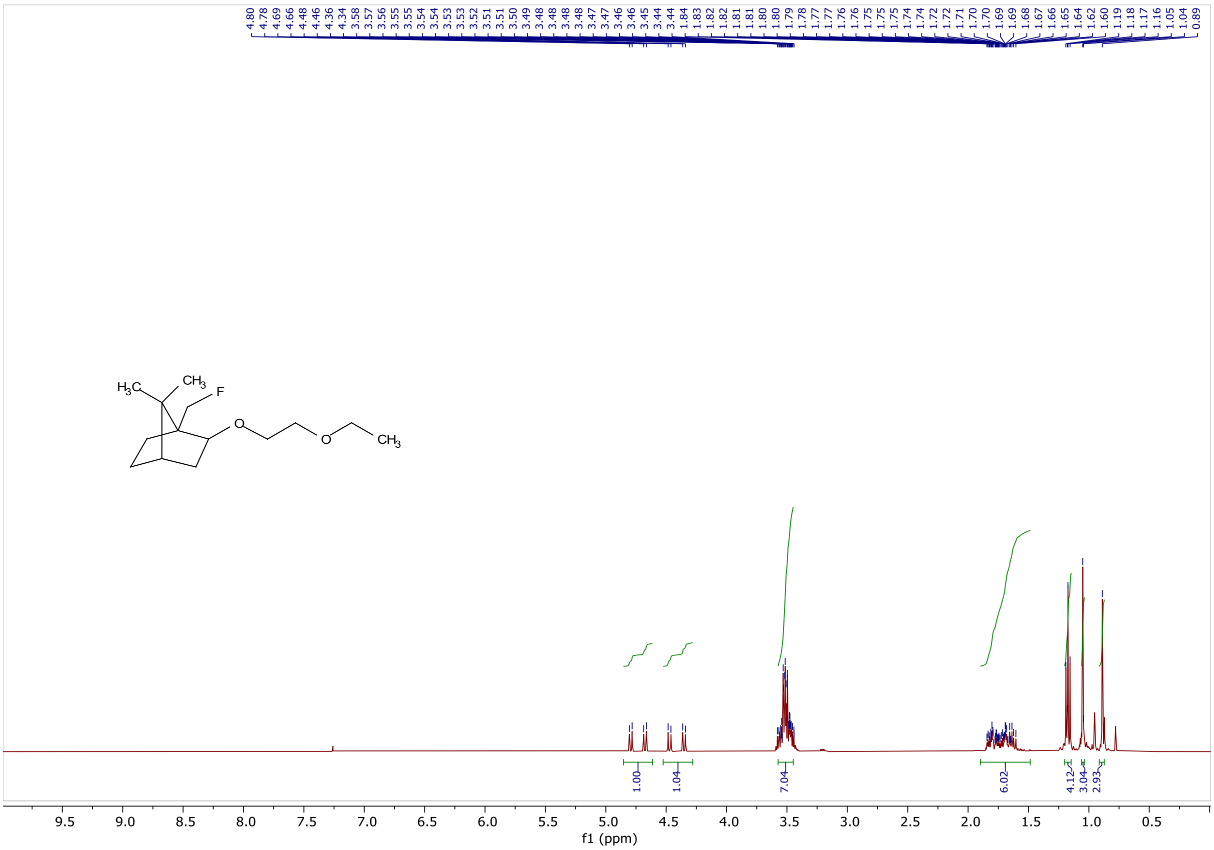

400 MHz ^{1}H-NMR spectrum of **4i** ($CDCl_3$)

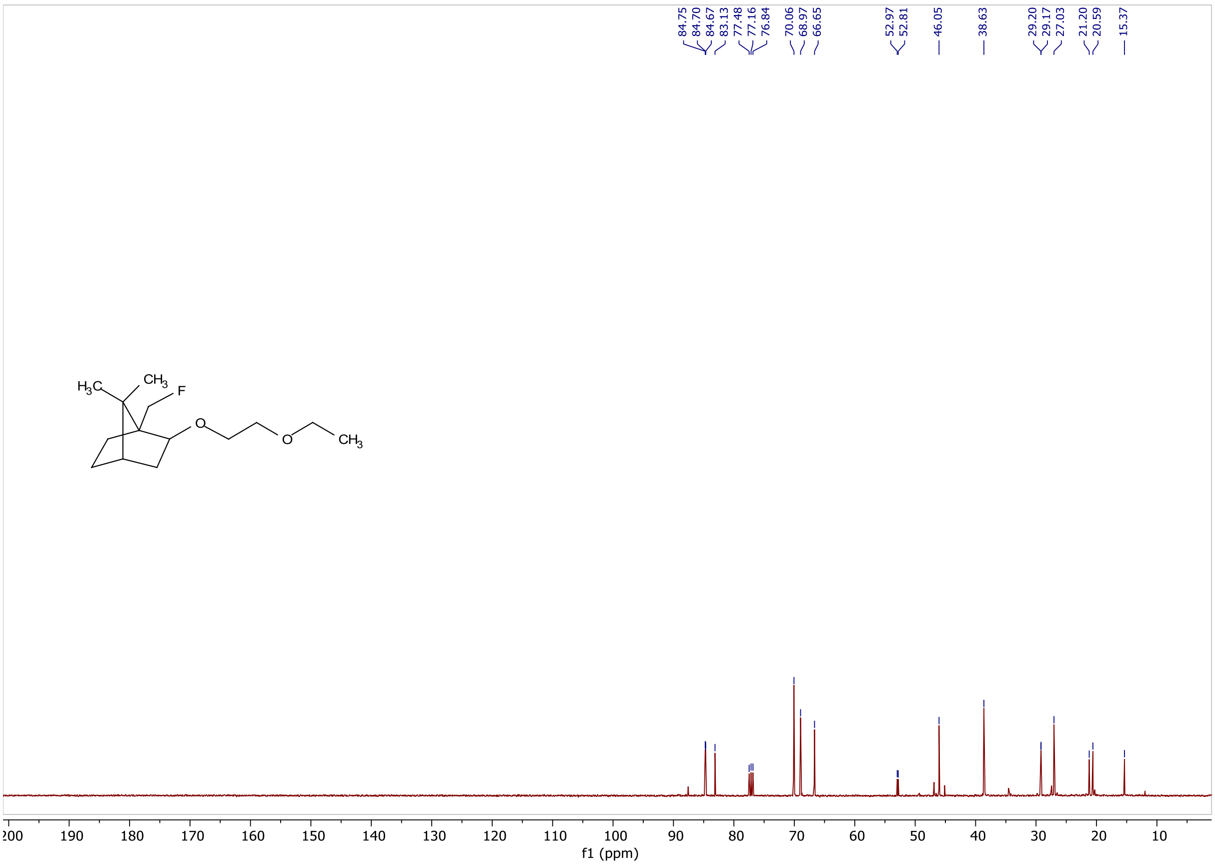

101 MHz ^{13}C-NMR spectrum of **4i** ($CDCl_3$)

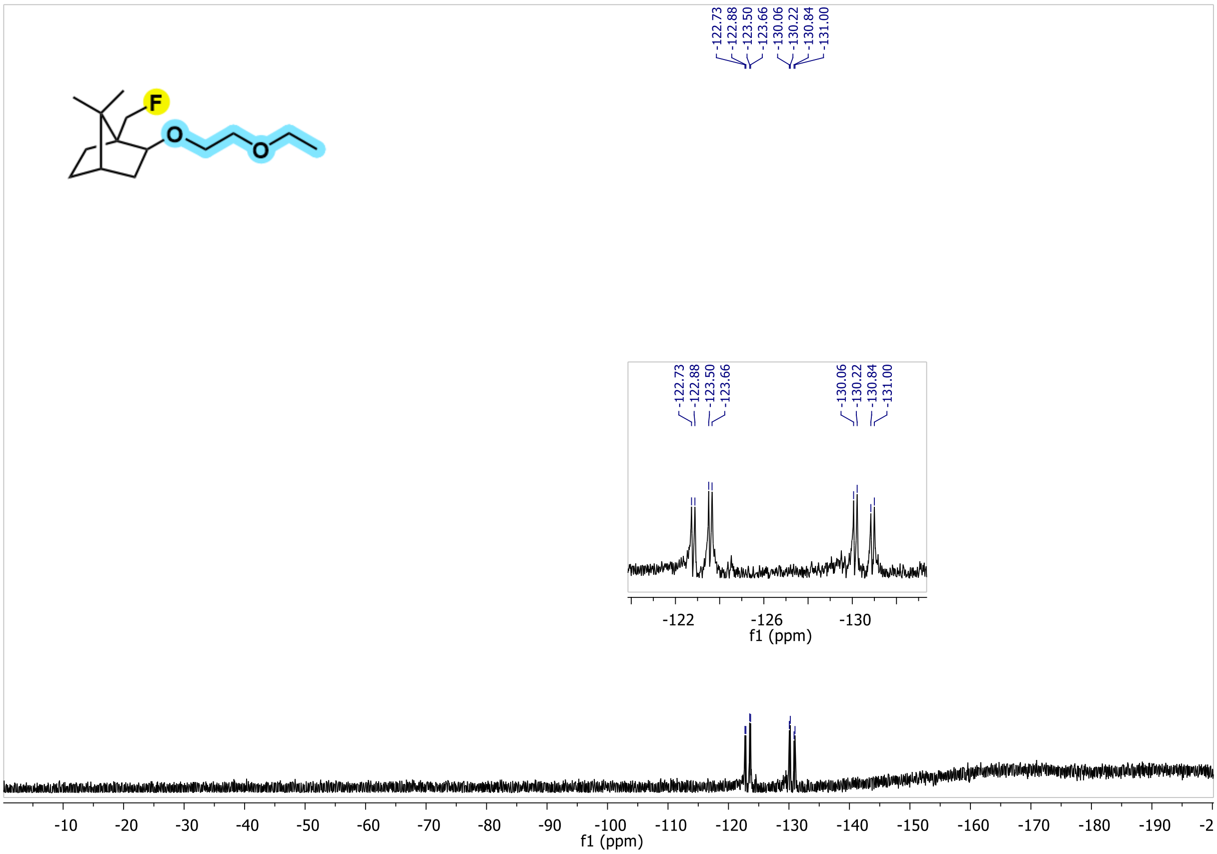

376 MHz ^{19}F-NMR spectrum of **4i** ($CDCl_3$)

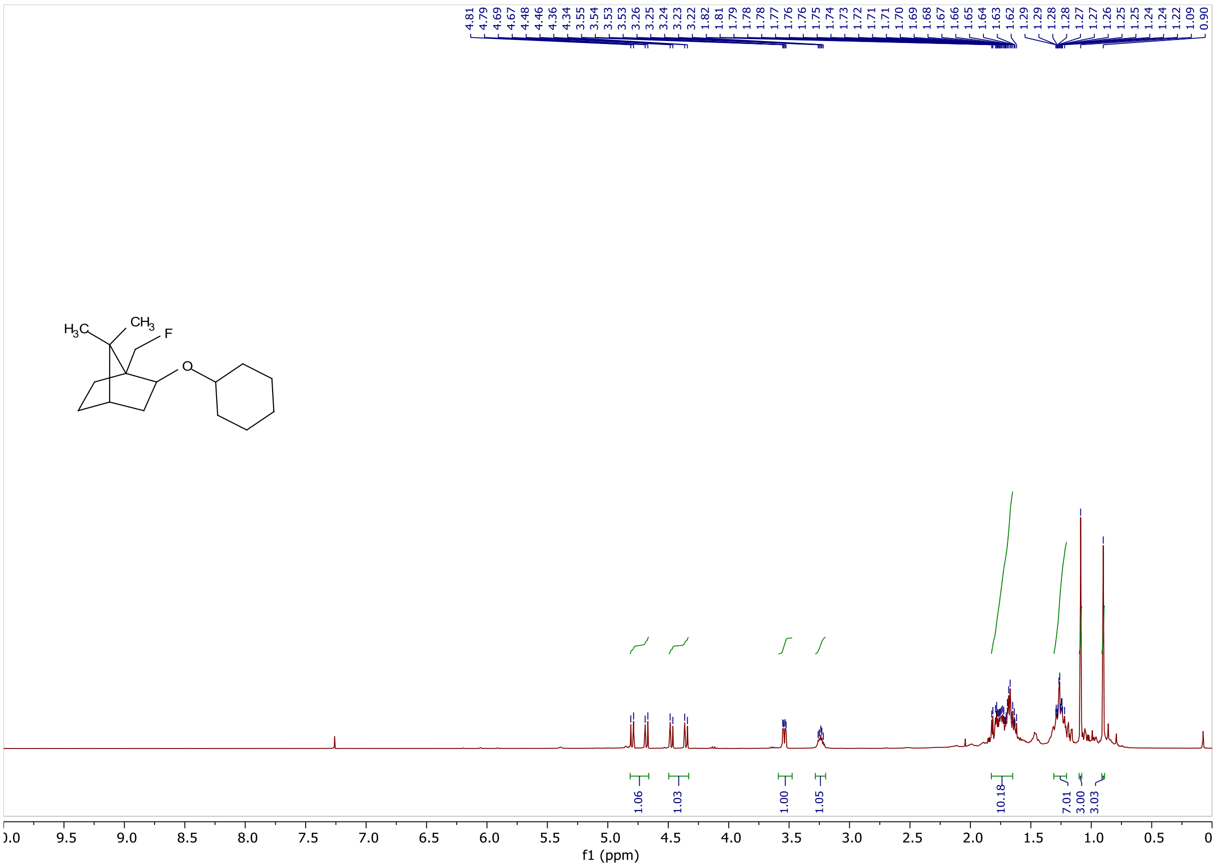

400 MHz ^{1}H-NMR spectrum of **4j** ($CDCl_3$)

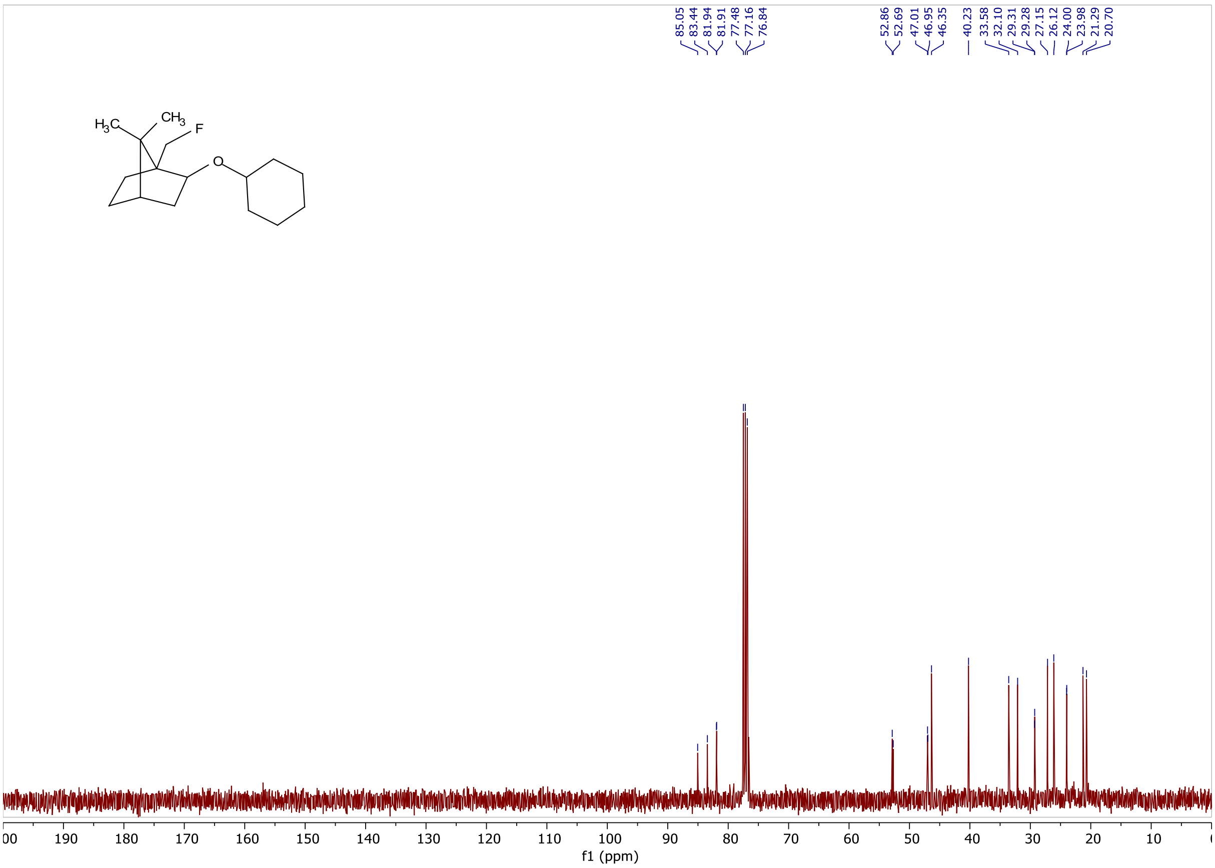

101 MHz ^{13}C-NMR spectrum of **4j** ($CDCl_3$)

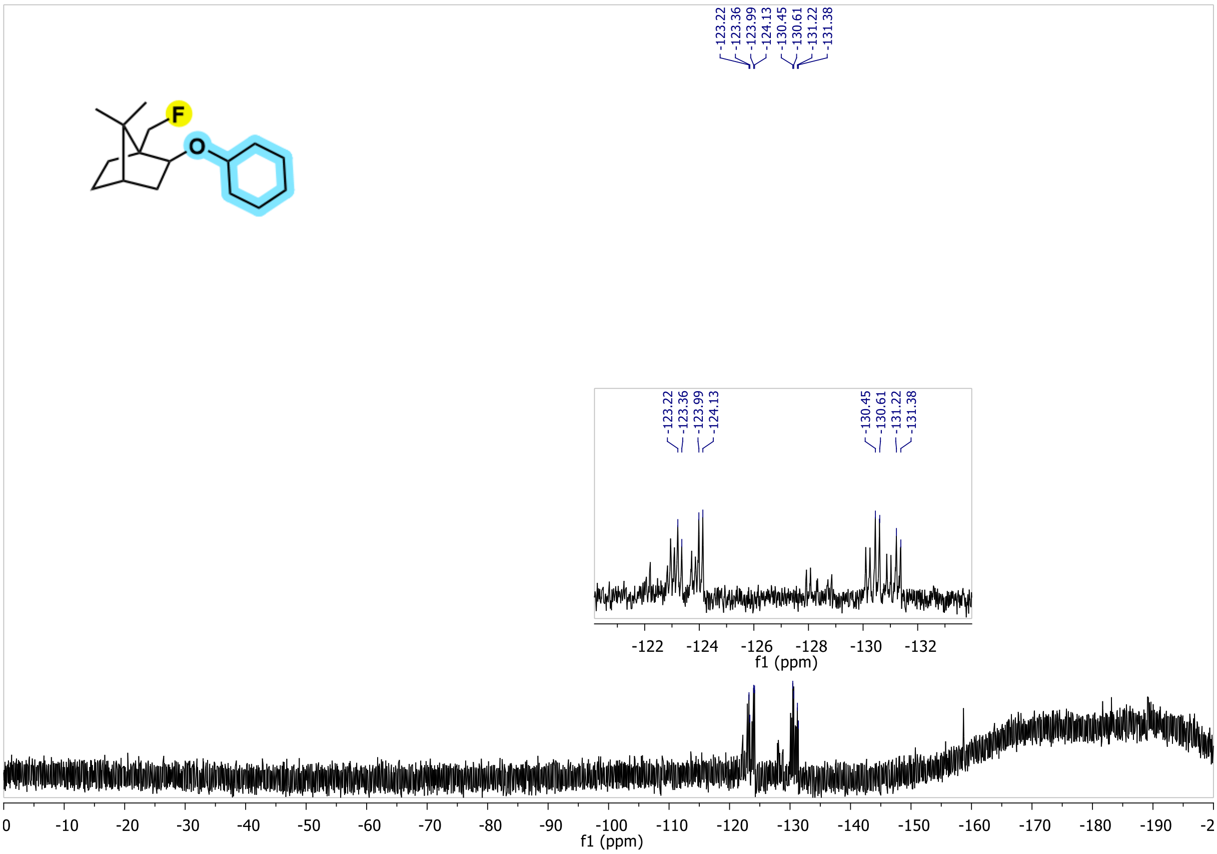

376 MHz ^{19}F-NMR spectrum of **4j** ($CDCl_3$)

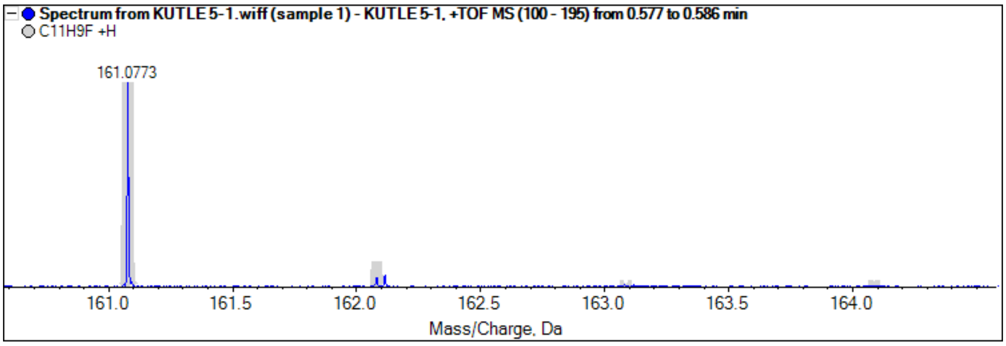

(TOF MS) spectrum of **3a**.

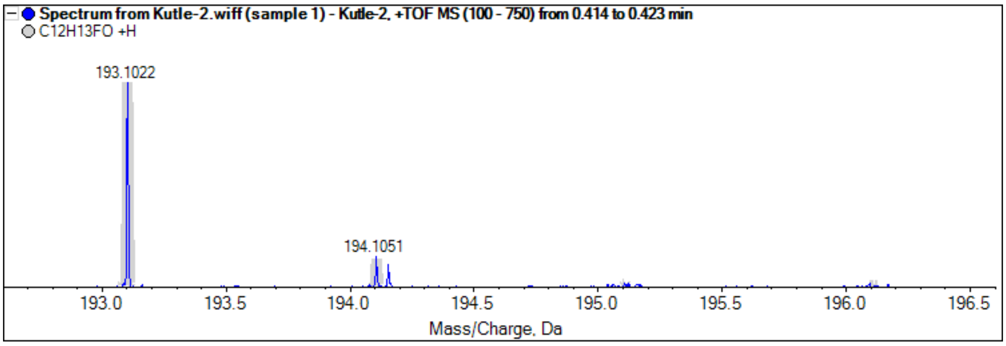

(TOF MS) spectrum of **3b**.

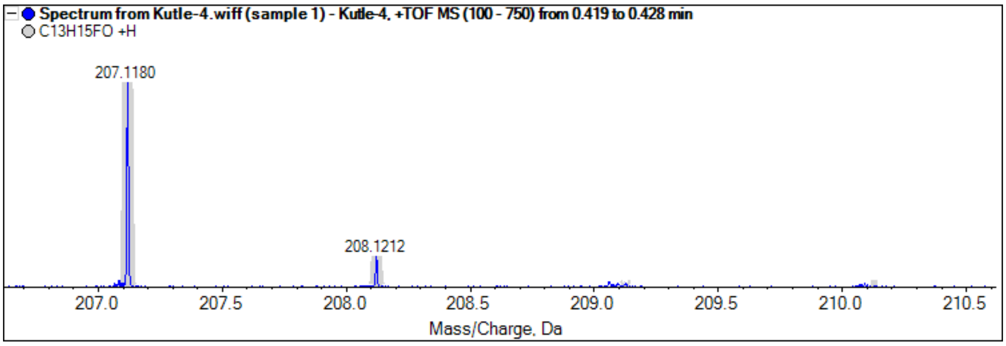

(TOF MS) spectrum of **3c**.

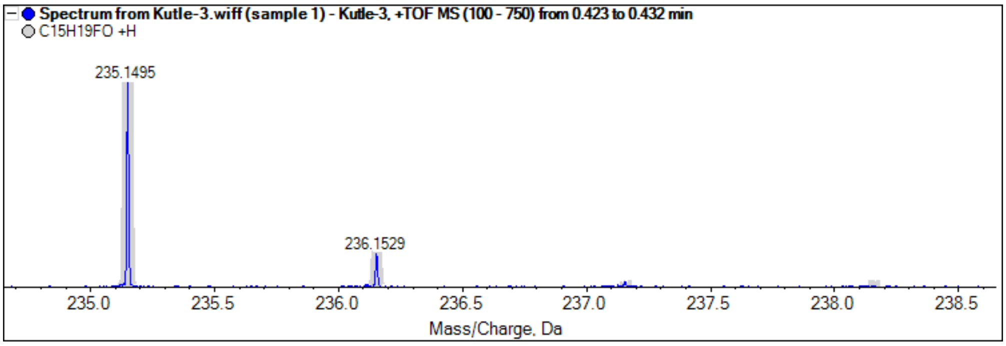

(TOF MS) spectrum of **3d**.

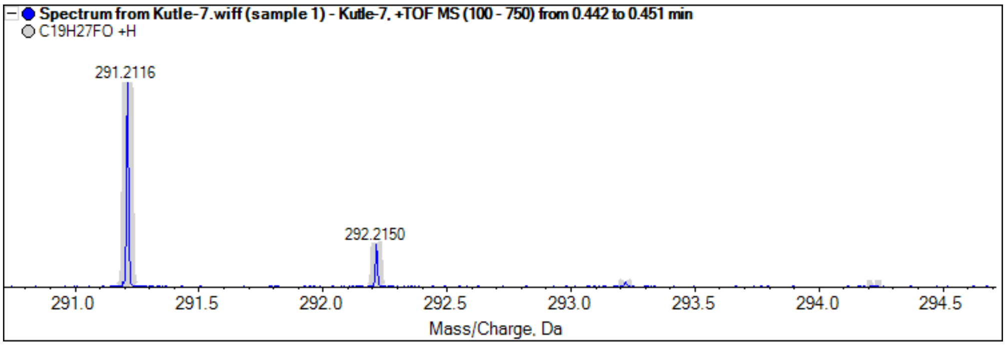

(TOF MS) spectrum of **3e**.

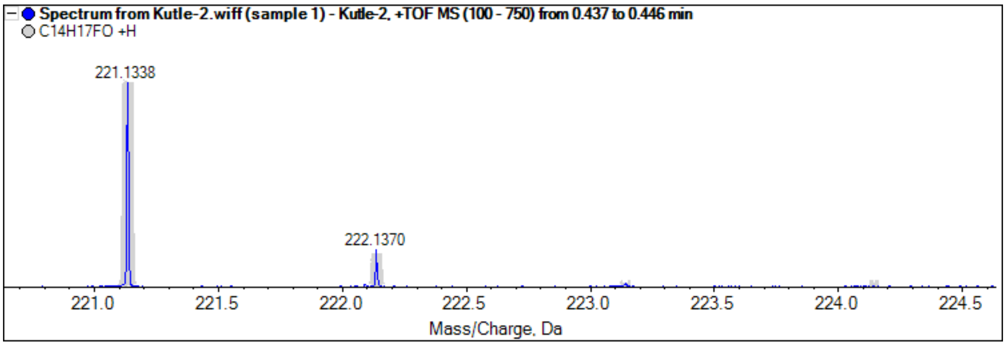

(TOF MS) spectrum of **3f**.

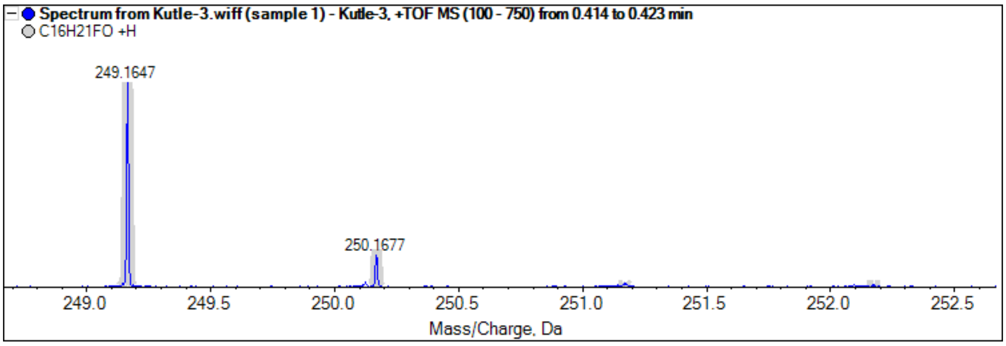

(TOF MS) spectrum of **3g**.

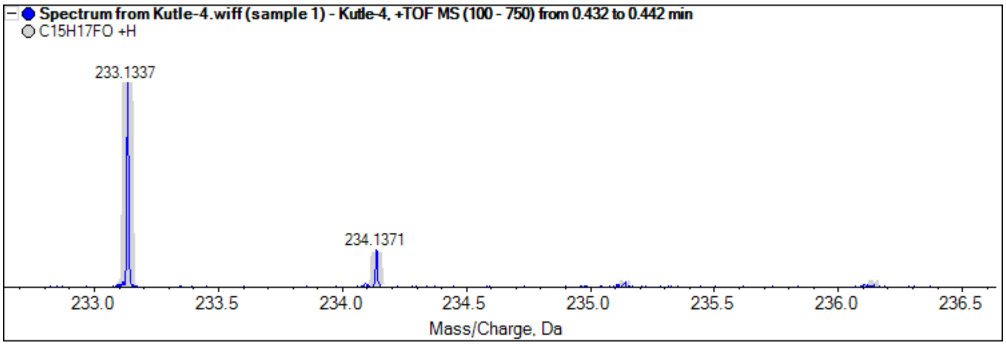

(TOF MS) spectrum of **3h**.

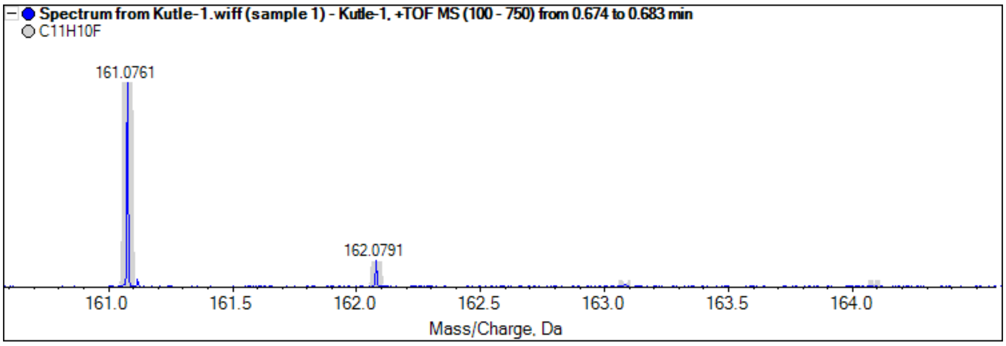

(TOF MS) spectrum of **3i**.

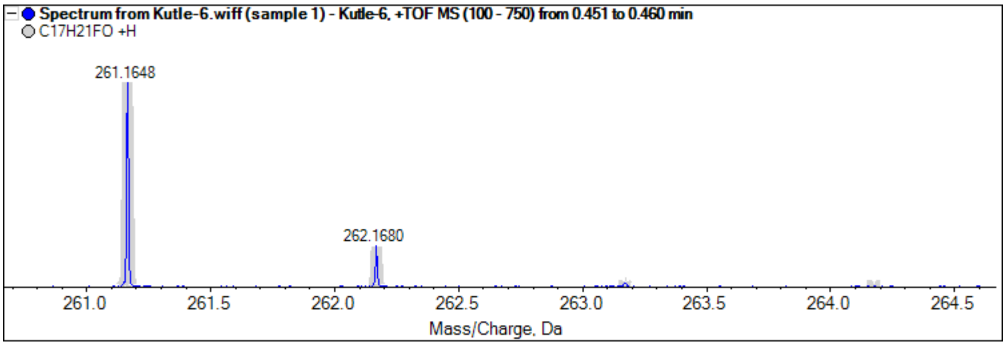

(TOF MS) spectrum of **3j**.

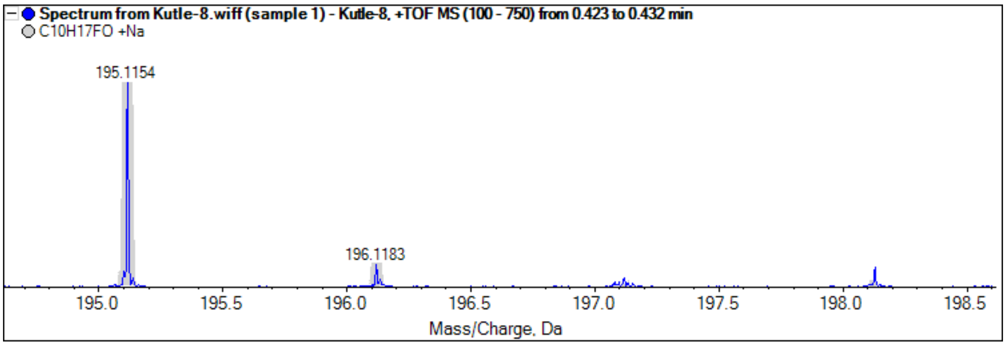

(TOF MS) spectrum of **4a**.

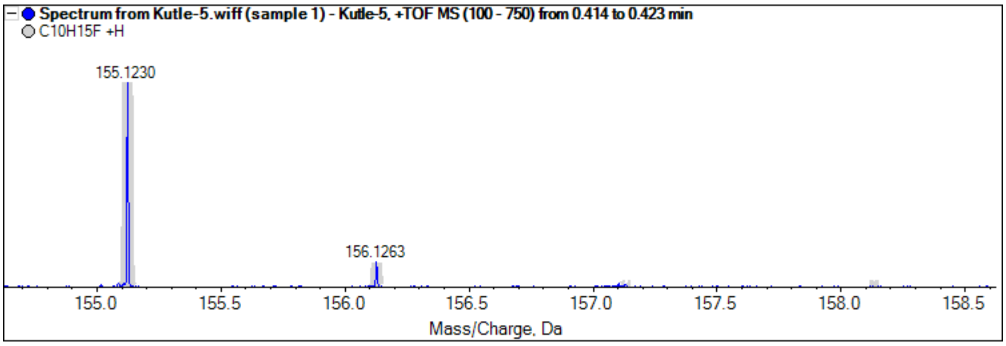

(TOF MS) spectrum of **4b**.

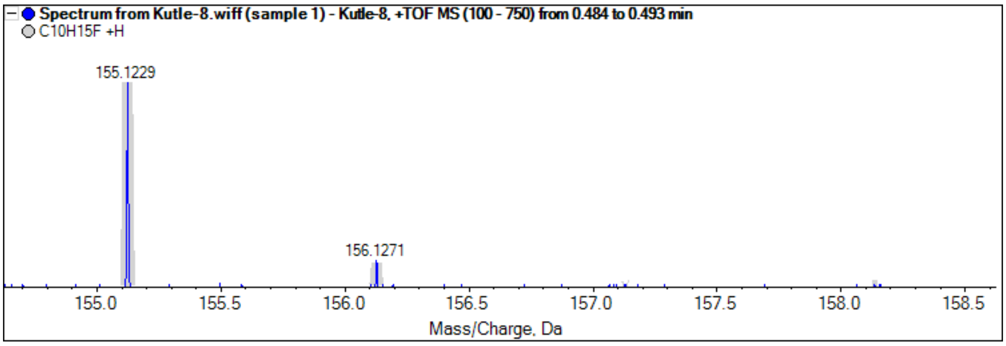

(TOF MS) spectrum of **4c**.

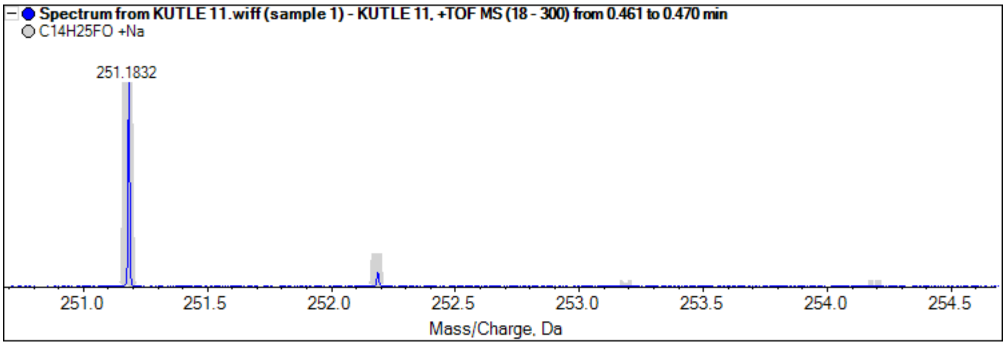

(TOF MS) spectrum of **4d**.

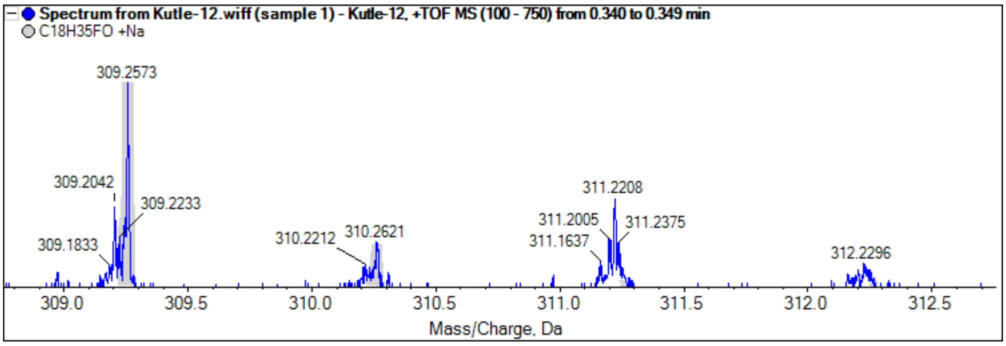

(TOF MS) spectrum of **4e**.

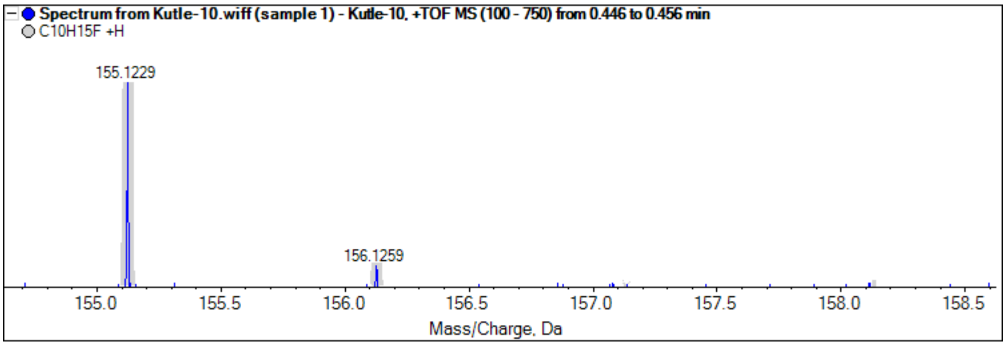

(TOF MS) spectrum of **4f**.

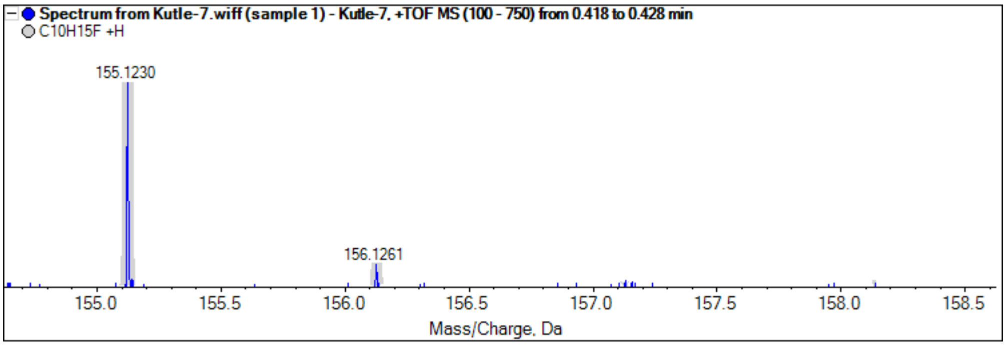

(TOF MS) spectrum of **4g**.

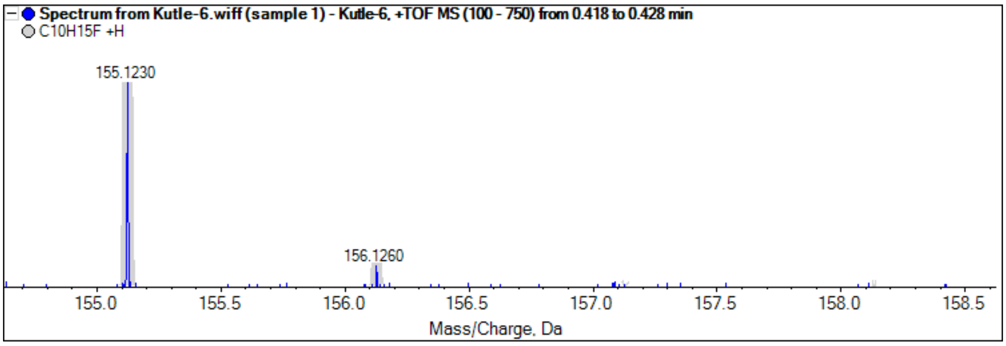

(TOF MS) spectrum of **4h**.

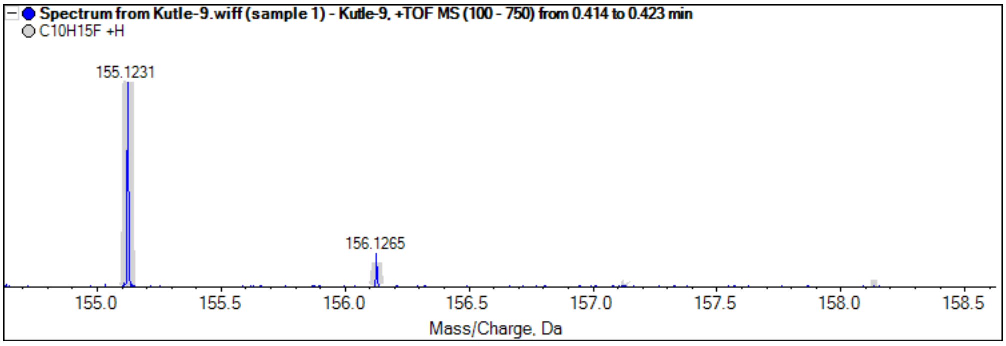

(TOF MS) spectrum of 4i.

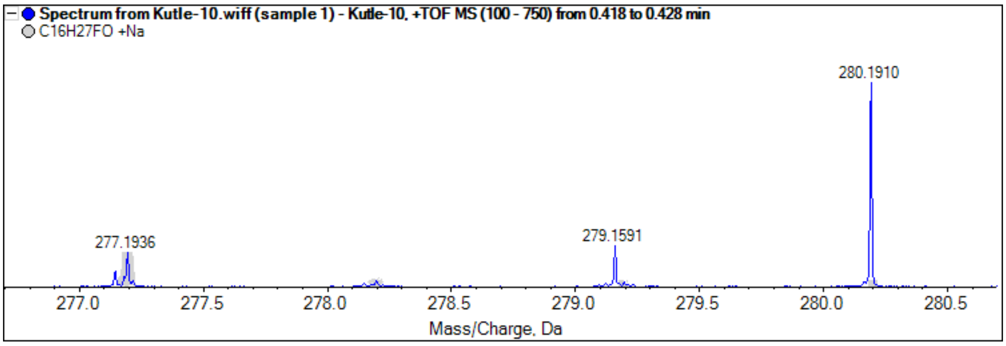

(TOF MS) spectrum of **4i**.
